# Nickel(II) and Palladium(II) Complexes with η^5^:κ^1^(*N*)-Coordinated Dicarbollide Ligands Containing Pendant Pyridine Group

**DOI:** 10.3390/ijms242015069

**Published:** 2023-10-11

**Authors:** Dmitriy K. Semyonov, Marina Yu. Stogniy, Sergey A. Anufriev, Sergey V. Timofeev, Kyrill Yu. Suponitsky, Igor B. Sivaev

**Affiliations:** 1 A.N. Nesmeyanov Institute of Organoelement Compounds, Russian Academy of Sciences, 28 Vavilov Str., 119991 Moscow, Russia; dksemyonov@mail.ru (D.K.S.); trueman476@mail.ru (S.A.A.); timofeev@ineos.ac.ru (S.V.T.); kirshik@yahoo.com (K.Y.S.); sivaev@ineos.ac.ru (I.B.S.); 2 M.V. Lomonosov Institute of Fine Chemical Technology, MIREA—Russian Technological University, 86 Vernadsky Av., 119571 Moscow, Russia; 3 Basic Department of Chemistry of Innovative Materials and Technologies, G.V. Plekhanov Russian University of Economics, 36 Stremyannyi Line, 117997 Moscow, Russia

**Keywords:** *nido*-carborane, pyridine, nickel, palladium, half-sandwich metal complexes, synthesis, structure

## Abstract

A series of *C*- and *B*-substituted *nido*-carborane derivatives with a pendant pyridyl group was prepared. The synthesized compounds were used as ligands in the complexation reactions with bis(triphenylphosphine)nickel(II) and palladium(II) chlorides to give six new metallacomplexes with unusual η^5^:κ^1^(*N*)-coordination of the metal center. The single crystal structures of 1-(NC_5_H_4_-2′-S)-1,2-C_2_B_10_H_11_, 1-(NC_5_H_4_-2′-CH_2_S)-1,2-C_2_B_10_H_11_, Cs [7-(NC_5_H_4_-2′-CH_2_S)-7,8-C_2_B_9_H_11_] *closo*- and *nido*-carboranes and 3-Ph_3_P-3-(4(7)-NC_5_H_4_-2′-S)-*closo*-3,1,2-NiC_2_B_9_H_10_ and 3-Ph_3_P-3-(4(7)-NC_5_H_4_-2′-CH_2_S)-*closo*-3,1,2-NiC_2_B_9_H_10_ metallacarboranes were determined using single crystal X-ray diffraction.

## 1. Introduction

*nido*-Carborane [*nido*-7,8-C_2_B_9_H_12_]^−^ is one of the most important members of the family of polyhedral boron hydrides, located at the junction of inorganic and organic chemistry. *nido*-Carborane (from Latin *nidus*, meaning “nest”) is formed upon the removal of the boron atom adjacent to the carbon atoms from the icosahedral *ortho*-carborane 1,2-C_2_B_10_H_12_, which has the *closo*-structure (a corruption of *clovo*, from Latin *clovis*, meaning “cage”), under the action of strong nucleophiles and has an open pentagonal face [1,2]. After the removal of *endo*-hydrogen from *nido*-carborane, the resulting dicarbollide anion [*nido*-1,2-C_2_B_9_H_11_]^2-^ can act as a ligand similar to a cyclopentadienyl one [3,4,5,6,7,8]. It is known that transition metal complexes with cyclopentadienyl ligands containing pendant donor groups are good catalysts for various organic reactions [9,10,11,12,13,14,15,16], and some of them are also promising luminescent materials [17,18]. One such pendant substituent is the pyridyl group [19,20]. In addition to the purely scientific interest associated with various types of coordination of such ligands [21,22,23,24], it was shown that titanium complexes with the cyclopentadienyl ligand containing the 2-picolyl substituent exhibit high catalytic activity during the ethylene polymerization reaction [25]. Therefore, the synthesis of *nido*-carboranes with a pendant pyridyl group and metallacarboranes based on them is of considerable interest.

To date, the synthesis of many *ortho*-carborane derivatives with a pyridyl group as a substituent has been described [26,27,28,29,30,31,32,33,34,35,36,37,38], most of which were obtained by the reaction of lithium derivatives of carboranes with pyridylaldehydes [33,34,35,36,37,38]. A number of transition metal complexes of various structures with pyridyl *ortho*-carborane ligands were synthesized as well [28,29,31,38,39,40,41,42,43,44,45,46,47,48]. However, only a few examples of *nido*-carborane derivatives with a pyridyl group have been described [39,49,50,51], and a few transition metal complexes (metallacarboranes) based on them [50]. This is in sharp contrast to *nido*-carborane derivatives with other pendant substituents, such as the Me_2_N group, based on which numerous metallacarboranes have been prepared [52,53,54,55,56,57], and it motivates our interest in research in this area.

**Figure 1:**
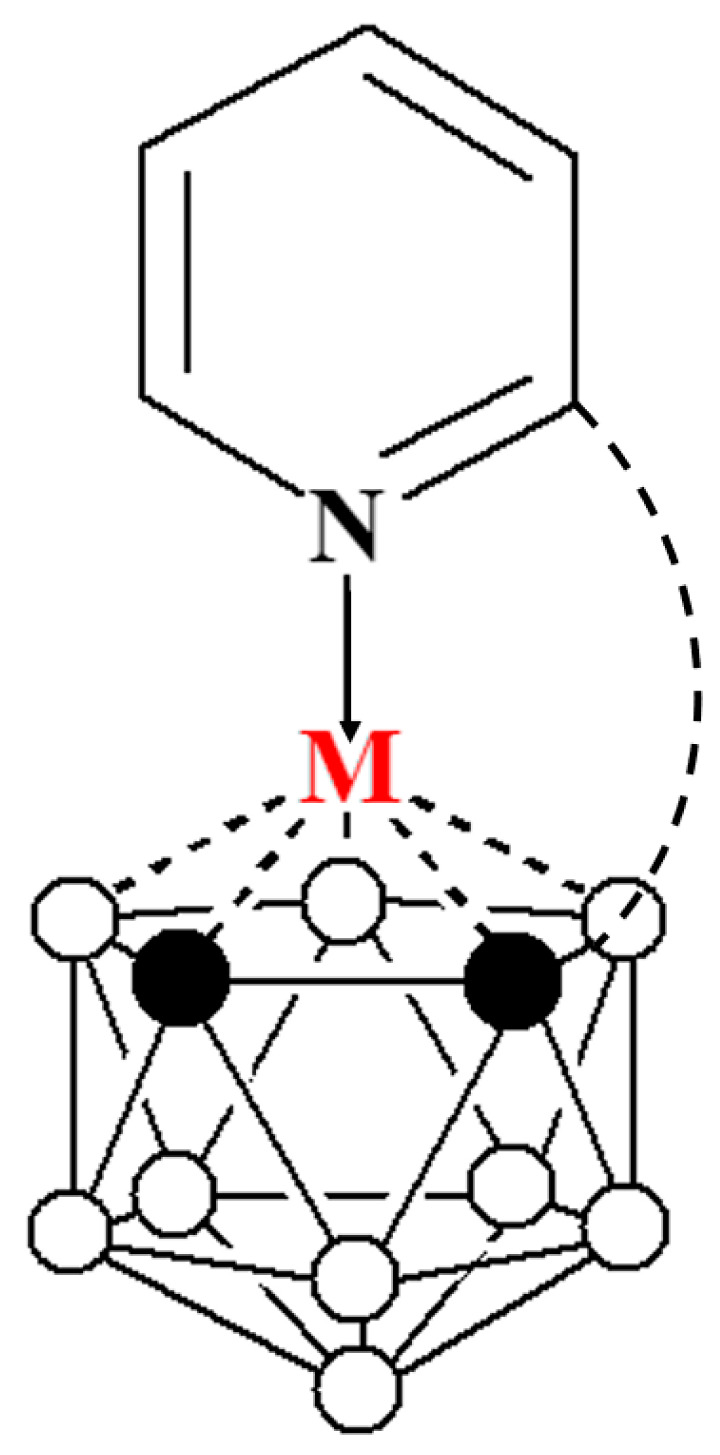
General view of metal complexes based on *nido*-carboranes with pendant pyridine group.

In this contribution we describe the synthesis of new *nido*-carborane derivatives with a pendant pyridine group and nickel(II) and palladium(II) complexes thereof (Figure 1).

## 2. Results and Discussion

### 2.1. Design of Dicarbollide Ligands with Pendant Pyridine Group: General Principles

There are two main points to consider when designing dicarbollide ligands with a pendant pyridyl group. The first of these is the presence and length of a spacer between the carborane cage and the pyridyl ring. This determines the size and stability of the metallocycle formed during coordination. Clearly, in the absence of such a spacer [49], the formation of only a strained four-membered metallocycle is possible, which is unfavorable for most *d*-metals. Indeed, in structurally characterized iridium complexes with pyridyl substituted *nido*-carborane [7-(NC_5_H_4_-2′)-7,8-C_2_B_9_H_11_]^−^, the latter is coordinated to the metal atom by the κ^2^-type through the nitrogen atom of the pyridyl group and the *BH* group of *nido*-carborane [58], rather than by the η^5^:κ^1^-type. At the same time, in the case of ligands with a monoatomic spacer between the carborane cage and the pyridyl ring, for example, [7-(NC_5_H_4_-2′-CH_2_)-7,8-C_2_B_9_H_11_]^−^, metal coordination occurs with the formation of a stable five-membered metallocycle according to the η^5^:κ^1^-type [50]. As for complexes based on *nido*-carborane with a diatomic spacer between the carborane cage and the pyridine heterocycle, to the best of our knowledge, there is only one such complex [3,3-(κ^2^(*N,O*)-NC_5_H_4_-2″-C(O)O)-3-(κ^1^(*N*)-1-NC_5_H_4_-2′-C(OH)H)-3,1,2-CoC_2_B_9_H_10_], which was accidentally obtained by leaving the *ortho*-carborane complex *trans*-[Co(κ^2^(*N,O*)-1-NC_5_H_4_-2′-C(O)H-1,2-C_2_B_10_H_11_)_2_] in an acetone solution for several days or weeks under air [40].

The second point is the position of the substitution in the *nido*-carborane basket. Unlike the cyclopentadienide ligand, in which all the carbon atoms in the five-membered ring are identical, the pentagonal face of the dicarbollide ligand is formed by two carbon atoms and three boron atoms. In this case, by introducing a substituent at the carbon atom or boron atom, it is possible to vary both the properties of the ligand itself and the properties of the metal complexes based on it. Firstly, this is due to the different electronic effects of the *nido*-carborane cage substituted at the boron and carbon atoms, which in the case of a short spacer can have a significant effect on the pendant donor group. Another less obvious point that can affect the stability and reactivity of metal complexes is the mutual orientation of the ligands. Due to the non-equivalence of atoms in the pentagonal face of the dicarbollide ligand, they interact differently with the metal atom, which leads to energetic inequality of different conformations due to the rotation of the dicarbollide ligand around the M–B(10) axis. In particular, for complexes of the *d*^8^ metal ions, such as Ni(II), Pd(II), and Pt(II), the preferred orientation is one in which the angle *θ* between the L–M–L plane and the B(8}–M–Center(C(1)–C(2)) planes is 90° [59,60,61,62]. Another feature is the displacement of the metal atom from the center of the pentagonal face of the ligand towards the boron atoms, the so-called “slippage” of the dicarbollide ligand, which is especially characteristic of nickel(II) bis(dicarbollide) complexes [63,64]. The introduction of substituents at the carbon atoms into the dicarbollide ligand can lead to a significant deviation of the angle *θ* from the ideal value due to steric repulsion between the ligands [65,66], and in extreme cases, this even results in the isomerization of the dicarbollide ligand [67,68]. It is clear that the position of attachment of the pendant donor group to the dicarbollide ligand will have a significant effect on the structure, and consequently, the properties of the resulting metal complexes [52,69,70].

### 2.2. Synthesis of nido-Carborane Derivatives with Pendant Pyridine Group

**Scheme 1:**
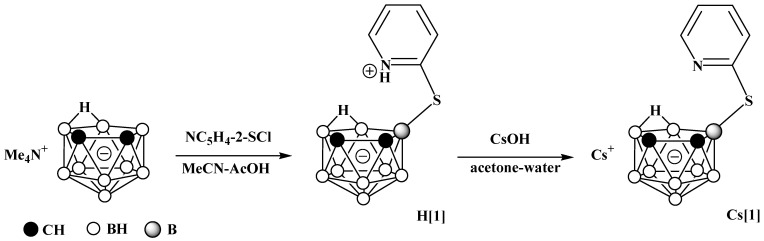
Synthesis of Cs[9-(NC_5_H_4_-2′-S)-7,8-C_2_B_9_H_11_] (**Cs[1]**).

To prepare metallacarboranes with a pendant pyridyl group attached to the boron atom of the dicarbollide ligand, we decided to use the 9-pyridylsulfenyl derivative of *nido*-carborane [9-(HNC_5_H_4_-2′-S)-7,8-C_2_B_9_H_11_] described in the literature [51]. First, via the reaction of the tetramethylammonium salt of *nido*-carborane with 2-pyridylsulfenyl chloride in a mixture of acetonitrile and acetic acid, the *N*-protonated derivative [9-(HNC_5_H_4_-2′-S)-7,8-C_2_B_9_H_11_] (**H[1]**) was obtained, which was then converted to the cesium salt Cs[9-(NC_5_H_4_-2′-S)-7,8-C_2_B_9_H_11_] (**Cs[1]**) via treatment with CsOH in aqueous acetone (Scheme 1).

The obtained carboranes were characterized using methods of ^1^H, ^13^C, and ^11^B NMR and IR spectroscopy and mass spectrometry (See Supplementary Materials, Figure S1–S70 and Table S1). The ^11^B NMR spectrum of **Cs[1]** in acetone-*d*_6_ contains a singlet at −3.1 ppm and seven doublets at −6.8, −15.6, −18.4, −22.5, −24.3, −30.6, and −37.8 ppm with an integral intensity ratio of 1:1:1:2:1:1:1:1, which is significantly different from the spectrum of the *N*-protonated form **H[1]** [51], indicating a rather strong interaction between the carborane cage and the pyridine ring. The ^1^H NMR spectrum of **Cs[1]**, in addition to the signals of the CH and BH groups of the *nido*-carborane cage, contains a set of signals of the pyridyl group, which appears in the form of two doublets at 8.22 and 7.67 ppm and two triplets at 7.49 and 6.87 ppm.

**Scheme 2:**
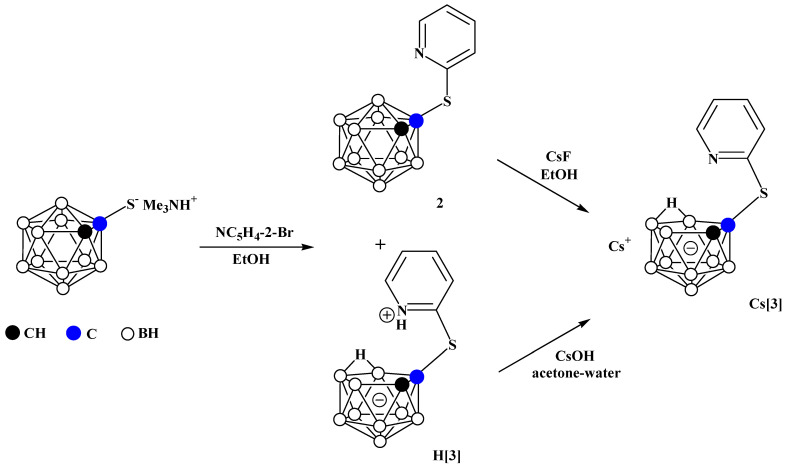
Synthesis of Cs[7-(NC_5_H_4_-2′-S)-7,8-C_2_B_9_H_11_] (**Cs[3]**).

To obtain a related *nido*-carborane with a pendant pyridyl group attached to the carbon atom, as a development of the known approach to the arylation and heteroarylation of 1-mercapto-*ortho*-carborane [71,72,73,74], we used the reaction of the trimethylammonium salt of 1-mercapto-*ortho*-carborane with 2-bromopyridine. The reaction in refluxing ethanol gave a mixture of the expected pyridyl derivative of *ortho*-carborane 1-(NC_5_H_4_-2′-S)-1,2-C_2_B_10_H_11_ (**2**) and its deboronation product as the N-protonated intramolecular salt [7-(HNC_5_H_4_-2′-S)-7,8-C_2_B_9_H_11_] (**H[3]**), which were separated using column chromatography on silica followed by the conversion of the latter to the cesium salt Cs[7-(NC_5_H_4_-2′-S)-7,8-C_2_B_9_H_11_] (**Cs[3]**) (Scheme 2). The cesium salt **Cs[3]** was also obtained via the deboronation of the corresponding *ortho*-carborane **2** with CsF in refluxing ethanol (Scheme 2).

In the ^1^H NMR spectrum of **Cs[3]**, the signals of the pyridyl group appear as a doublet at 8.30 ppm, a triplet at 7.70 ppm, a doublet at 7.24 ppm, and a triplet at 7.02 ppm, demonstrating a significant difference in the electronic effects of the *nido*-carborane cage substituted at the boron and carbon atoms.

**Figure 2:**
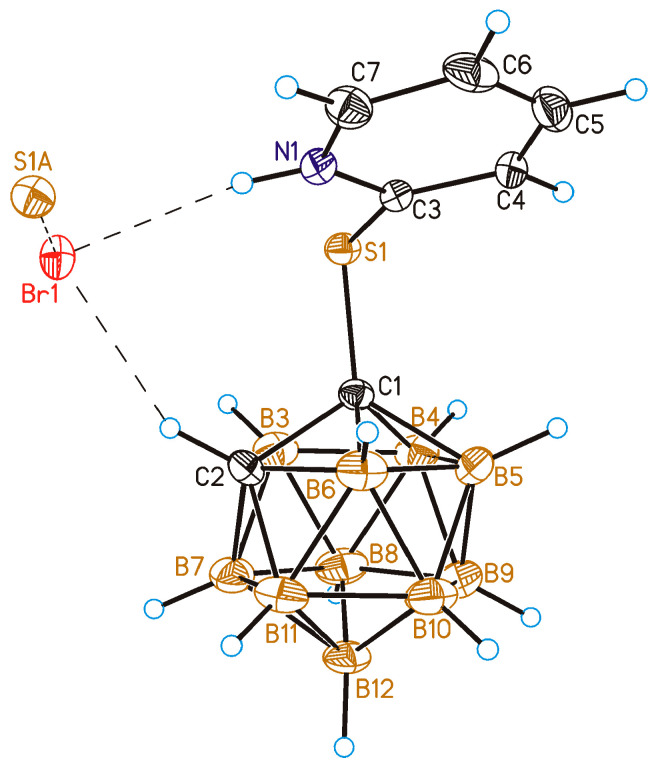
General view of 1-(NC_5_H_4_-2′-S)-1,2-C_2_B_10_H_11_·HBr showing numbering scheme. Thermal ellipsoids are given at 50% probability level. The closest contacts formed by the Br^−^ anion are shown by dashed lines.

The solid-state structure of 1-(NC_5_H_4_-2′-S)-1,2-C_2_B_10_H_11_·HBr (**2·HBr**) (see Supplementary Materials) was determined using single crystal X-ray diffraction (Figure 2).

**Scheme 3:**
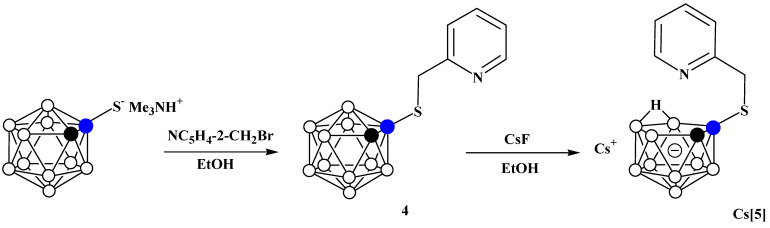
Synthesis of Cs[7-(NC_5_H_4_-2′-CH_2_S)-7,8-C_2_B_9_H_11_] (**Cs[5]**).

The reaction of the trimethylammonium salt of 1-mercapto-*ortho*-carborane with 2-bromomethyl pyridine followed by the deboronation of the resulting pyridine-containing *ortho*-carborane 1-(NC_5_H_4_-2′-CH_2_S)-1,2-C_2_B_10_H_11_ (**4**) was used to prepare the *nido*-carborane derivative with a longer spacer between the carborane cage and the pendant pyridyl group Cs[7-(NC_5_H_4_-2′-CH_2_S)-7,8-C_2_B_9_H_11_] (**Cs[5]**) (Scheme 3). Previously, this approach was used for the synthesis of various alkylsulfenyl derivatives of *ortho*- and *nido*-carboranes including those containing various functional groups [75,76,77,78,79].

The obtained carboranes were characterized using methods of ^1^H, ^13^C, and ^11^B NMR and IR spectroscopy and mass spectrometry (See Supplementary Materials). In the ^1^H NMR spectrum of **4** in acetone-*d*_6_, the signal of the methylene group appears as a singlet at 4.42 ppm, whereas in the spectrum of **Cs[5]**, the signals of the methylene group appear as two doublets at 4.13 and 3.89 ppm (^2^*J*_HH_ = 12.8 Hz) due to chirality of the *C*-monosubstituted *nido*-carborane cage that causes protons to become diastereotopic and magnetically inequivalent.

**Figure 3:**
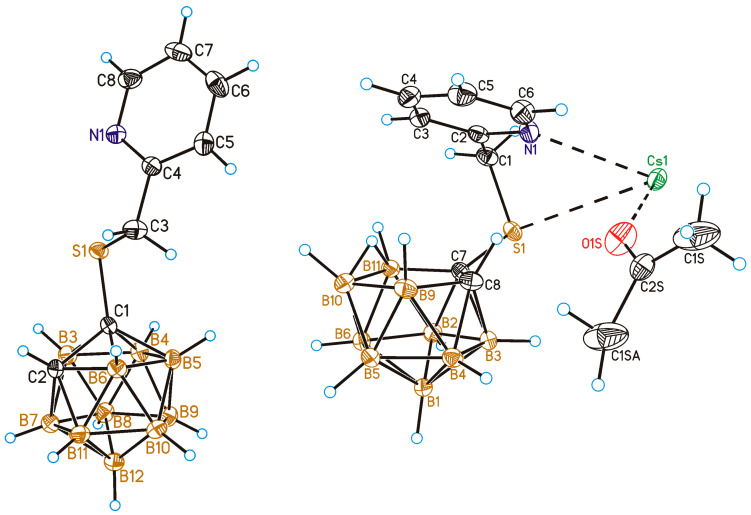
General view of 1-(NC_5_H_4_-2′-CH_2_S)-1,2-C_2_B_10_H_11_ (**4**) (**left**) and Cs[7-(NC_5_H_4_-2′-CH_2_S)-7,8-C_2_B_9_H_11_]·0.5Me_2_CO (**Cs[5]·0.5Me_2_CO**) (**right**) showing numbering scheme. Thermal ellipsoids are given at 50% probability level. The closest contacts formed by the Cs^+^ cation are shown by dashed lines.

The solid-state structures of 1-(NC_5_H_4_-2′-CH_2_S)-1,2-C_2_B_10_H_11_ (**4**) and Cs[7-(NC_5_H_4_-2′-CH_2_S)-7,8-C_2_B_9_H_11_]·0.5Me_2_CO (**Cs[5]·0.5Me_2_CO**) (see Supplementary Materials) were determined using single crystal X-ray diffraction (Figure 3).

### 2.3. Synthesis of Nickela- and Platinacarboranes with Pendant Chelating Pyridine Group

**Scheme 4:**
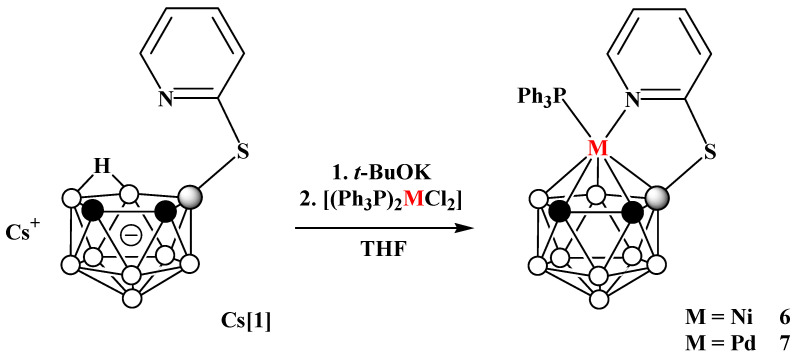
Synthesis of metallacarboranes 3-Ph_3_P-3-(4(7)-NC_5_H_4_-2′-S)-*closo*-3,1,2-NiC_2_B_9_H_10_ (**6**) and 3-Ph_3_P-3-(4(7)-NC_5_H_4_-2′-S)-*closo*-3,1,2-NiC_2_B_9_H_10_ (**7**).

The deprotonation of **Cs[1]** with *t*-BuOK in dry THF followed by the addition of triphenylphosphine complexes of nickel(II) or palladium(II) [(Ph_3_P)_2_MCl_2_] (M=Ni, Pd) results in the corresponding metallacarboranes 3-Ph_3_P-3-(4(7)-NC_5_H_4_-2′-S)-*closo*-3,1,2-MC_2_B_9_H_10_ (M=Ni (**6**), Pd (**7**)), which were isolated in moderate yields after column chromatography on silica (Scheme 4).

**Figure 4:**
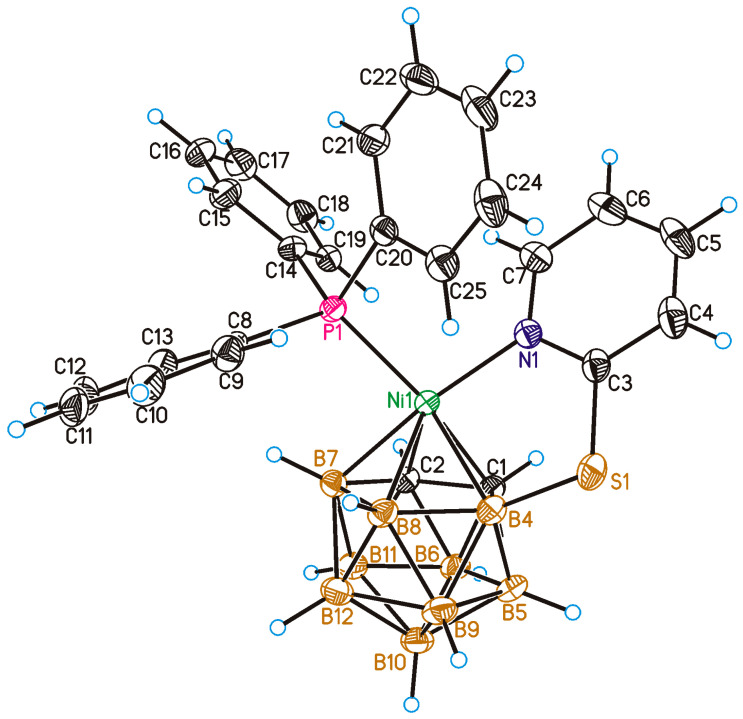
General view of 3-Ph_3_P-3-(4(7)-NC_5_H_4_-2′-S)-*closo*-3,1,2-NiC_2_B_9_H_10_ (**6**) showing numbering scheme. Thermal ellipsoids are given at 50% probability level.

The obtained metallacarboranes were characterized using methods of ^1^H, ^13^C, ^11^B, and ^31^P NMR spectroscopy, as well as IR and UV spectroscopy and mass spectrometry. The solid-state structure of 3-Ph_3_P-3-(4(7)-NC_5_H_4_-2′-S)-*closo*-3,1,2-NiC_2_B_9_H_10_ (**6**) was determined using single crystal X-ray diffraction (see Supplementary Materials). A general view of the nickelacarborane molecule is given in Figure 4.

The orientation of the σ-donor ligands (the pendant pyridine and triphenylphosphine) with respect to the dicarbollide ligand significantly deviates from the ideal orientation with the *θ* angle between the N(1)-Ni(1)-P(1) plane and the B(8)-Ni(1)-Center-(C(1)-C(2)) plane being ~ 60°, and the pyridyl group is rotated around the B(9)-S(1) bond toward the carbon atoms of the dicarbollide ligand (Figure 3). No noticeable “slippage” of the dicarbollide ligand was found.

**Scheme 5:**
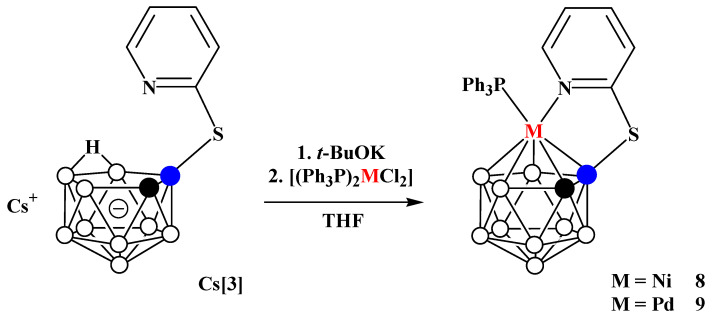
Synthesis of metallacarboranes 3-Ph_3_P-3-(1(2)-NC_5_H_4_-2′-S)-*closo*-3,1,2-NiC_2_B_9_H_10_ (**8**) and 3-Ph_3_P-3-(1(2)-NC_5_H_4_-2′-S)-*closo*-3,1,2-NiC_2_B_9_H_10_ (**9**).

In a similar way, metallacarboranes 3-Ph_3_P-3-(1(2)-NC_5_H_4_-2′-S)-*closo*-3,1,2-NiC_2_B_9_H_10_ (**8**) and 3-Ph_3_P-3-(1(2)-NC_5_H_4_-2′-S)-*closo*-3,1,2-PdC_2_B_9_H_10_ (**9**) were prepared starting from the *C*-substituted *nido*-carborane **Cs[3]** (Scheme 5). The obtained metallacarboranes were characterized using methods of ^1^H, ^13^C, ^11^B, and ^31^P NMR spectroscopy, as well as IR and UV spectroscopy and mass spectrometry. Unfortunately, we were unable to obtain crystals suitable for X-ray diffraction studies of single crystals.

**Scheme 6:**
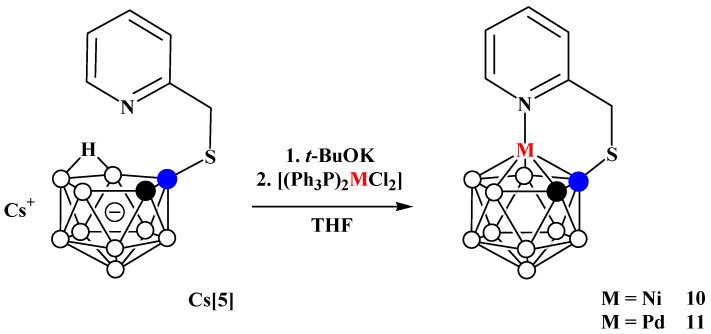
Synthesis of metallacarboranes 3-Ph_3_P-3-(1(2)-NC_5_H_4_-2′-CH_2_S)-*closo*-3,1,2-NiC_2_B_9_H_10_ (**10**) and 3-Ph_3_P-3-(1(2)-NC_5_H_4_-2′-CH_2_S)-*closo*-3,1,2-NiC_2_B_9_H_10_ (**11**).

Metallacarboranes with a more flexible spacer between the dicarbollide ligand and the pyridyl group 3-Ph_3_P-3-(1(2)-NC_5_H_4_-2′-CH_2_S)-*closo*-3,1,2-NiC_2_B_9_H_10_ (**10**) and 3-Ph_3_P-3-(1(2)-NC_5_H_4_-2′-CH_2_S)-*closo*-3,1,2-PdC_2_B_9_H_10_ (**11**) were synthesized in a similar way starting from the *C*-substituted *nido*-carborane **Cs[5]** (Scheme 6).

**Figure 5:**
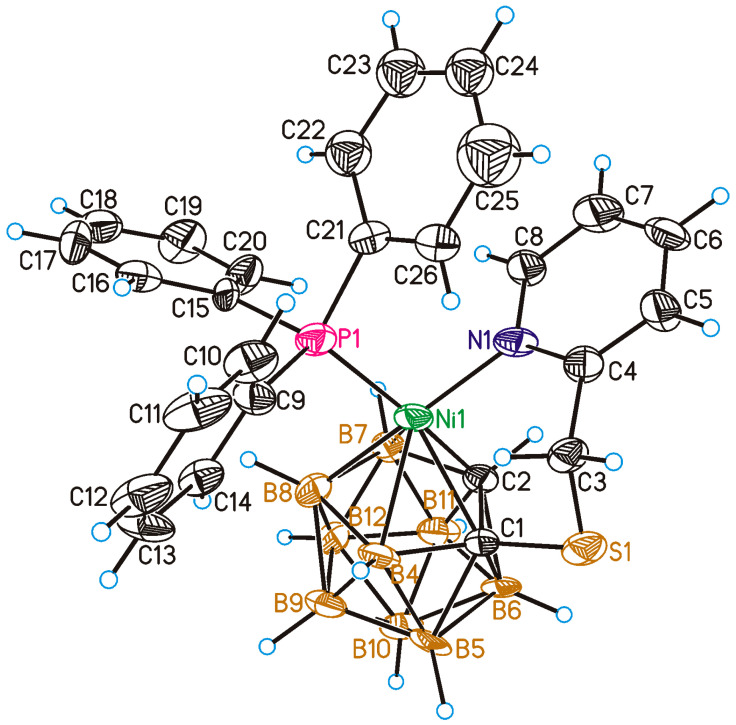
General view of 3-Ph_3_P-3-(4(7)-NC_5_H_4_-2′-CH_2_S)-*closo*-3,1,2-NiC_2_B_9_H_10_ (**10**) showing numbering scheme. Thermal ellipsoids are given at 50% probability level.

The obtained metallacarboranes were characterized using methods of ^1^H, ^13^C, ^11^B, and ^31^P NMR spectroscopy, as well as IR and UV spectroscopy and mass spectrometry. The solid-state structure of 3-Ph_3_P-3-(4(7)-NC_5_H_4_-2′-CH_2_S)-*closo*-3,1,2-NiC_2_B_9_H_10_ (**10**) was determined using single crystal X-ray diffraction (see Supplementary Materials). A general view of the nickelacarborane molecule is given in Figure 5.

The orientation of the σ-donor ligands (the pendant pyridine and triphenylphosphine) with respect to the dicarbollide ligand strongly deviates from the ideal orientation with the *θ* angle between the N(1)-Ni(1)-P(1) plane and the B(8)-Ni(1)-Center-(C(1)-C(2)) plane being ~16°. The six-membered ring Ni(1)-C(1)-S(1)-C(3)-C(4)-N(1) adopts a highly distorted boat conformation with a nickel atom and a methylene group located at the bow and stern (Figure 4). No noticeable “slippage” of the dicarbollide ligand was found.

## 3. Methods and Materials

### 3.1. Materials and Methods

The trimethylammonium salt of 1-mercapto-*ortho*-carborane [78], bis(triphenylphosphine)nickel(II) chloride [(Ph_3_P)_2_NiCl_2_] [80] and bis(triphenylphosphine)palladium(II) chloride [(Ph_3_P)_2_PdCl_2_] [81] were synthesized according to the literature described methods. 2-Bromopyridine and 2-(bromomethyl)-pyridine hydrobromide were purchased from Acros Organics and used without purification. Tetrahydrofuran was dried using standard procedure [82]. The reaction progress was monitored using thin-layer chromatography (Merck F254 silica gel on aluminum plates) and visualized using 0.5% PdCl_2_ in 1% HCl in aq. MeOH (1:10). Acros Organics silica gel (0.060–0.200 mm) was used for column chromatography. The NMR spectra at 400.1 MHz (^1^H), 128.4 MHz (^11^B), 100.0 MHz (^13^C), and 162 MHz (^31^P) were recorded with a Varian Inova 400 spectrometer. The residual signal of the NMR solvent relative to Me_4_Si was taken as an internal reference for ^1^H and ^13^C NMR spectra. ^11^B NMR spectra were referenced using BF_3_.Et_2_O as an external standard. ^31^P NMR spectra are given relative to 85% H_3_PO_4_ as an external standard. Infrared spectra were recorded on an FSM-2201 (INFRASPEC, Saint Petersburg, Russia) instrument. UV/Vis spectra were recorded with SF-2000 spectrophotometer (OKB SPECTR LLC, Saint Petersburg, Russia) using 1 cm cuvettes. High resolution mass spectra (HRMS) were measured on a Bruker micrOTOF II instrument using electrospray ionization (ESI).

### 3.2. Synthesis of Cs[9-(NC_5_H_4_-2′-S)-7,8-C_2_B_9_H_11_] (**Cs[1]**)

Compound **Cs[1]** was prepared from described in the literature *N*-protonated *[9-(HNC_5_H_4_-2′-S)-7,8-C_2_B_9_H_11_]* (**H[1]**) (0.30 g, 1.23 mmol) [51] by dissolving it in acetone (7 mL) and reprecipitated with aqueous CsOH (0.28 g, 1.85 mmol in 40 mL of water) to give 0.44 g (95% yield) of cesium salt of **5**. ^1^H NMR (acetone-*d*_6_, ppm): *δ* 8.22 (1H, d, *J* = 4.7 Hz, *H*_py_), 7.67 (1H, d, *J* = 8.1 Hz, *H*_py_), 7.49 (1H, t, *J* = 7.5 Hz, *H*_py_), 6.87 (1H, m, *H*_py_), 2.29 (1H, br s, C*H*_carb_), 1.86 (1H, br s, C*H*_carb_), 2.6 ÷ (−0.1) (8H, br m, B*H*), −2.38 (1H, br m, B*H*B). ^11^B NMR (acetone-*d*_6_, ppm): *δ* −3.1 (1B, s), −6.8 (1B, d, *J* = 138 Hz), −15.6 (1B, *J* = 138 Hz), −18.4 (2B, d, *J* = 160 Hz), −22.5 (1B, d, *J* = 151 Hz), −24.3 (1B, d, *J* = 139 Hz), −30.6 (1B, d, *J* = 137 Hz), −37.8 (1B, d, *J* = 141 Hz).

### 3.3. Synthesis of 1-(NC_5_H_4_-2′-S)-1,2-C_2_B_10_H_11_ (2), [7-(NC_5_H_5_-2′-S)-7,8-C2B9H11] (H[3]) and Cs[7-(NC5H4-2′-S)-7,8-C2B9H11] (**Cs[3]**)

2-Bromopyridine (0.24 mL, 2.55 mmol) was added to a solution of the trimethylammonium salt of 1-mercapto-*closo*-carborane (0.60 g, 2.55 mmol) in ethanol (30 mL) and the reaction mixture was refluxed for about 5 h, cooled to ambient temperature and evaporated to dryness in vacuum. The obtained product was isolated using column gradient elution chromatography on silica with CH_2_Cl_2_:acetone mixture (from 19:1 to 1:1) as an eluent to give 0.17 g (26% yield) of white crystalline product **2** and 0.17 g (27% yield) of white crystalline **H[3]**. Compound **H[3]** was dissolved in acetone (5 mL) and reprecipitated with aqueous CsOH (0.15 g, 1.00 mmol in 30 mL of water) to give 0.26 g (99% yield) of cesium salt of **Cs[3]**.

The cesium salt **Cs[2]** was also obtained by refluxing **2** (0.14 g, 0.55 mmol) in ethanol (10 mL) with cesium fluoride (0.17 g, 1.11 mmol) for 12 h. The precipitate formed was filtered off and the filtrate was evaporated under reduced pressure. The residue was dissolved in acetone (10 mL) and unreacted CsF was filtered off. The filtrate was evaporated *in vacuo* to give a white solid of **Cs[3]** (0.20 g, 97% yield).

Spectral data for **2**. ^1^H NMR (acetone-*d*_6_, ppm): *δ* 8.70 (1H, d, *J* = 6.3 Hz, *H*(6)_py_), 7.95 (1H, t, *J* = 7.4 Hz, *H*(4)_py_), 7.72 (1H, d, *J* = 7.6 Hz, *H*(3)_py_), 7.56 (1H, t, *J* = 6.2 Hz, *H*(5)_py_), 5.12 (1H, br s, C*H*_carb_), 3.0 ÷ 1.2 (10H, br m, B*H*). ^13^C NMR (acetone-*d*_6_, ppm): *δ* 152.1 (*C*(2)_py_), 151.1 (*C*(6)_py_), 138.5 (*C*(4)_py_), 130.9 (*C*(3)_py_), 125.4 (*C*(5)_py_), 74.1 (*C*_carb_S), 65.5 (*C*_carb_H). ^11^B NMR (acetone-*d*_6_, ppm): *δ* −1.90 (1B, d, *J* = 143 Hz), −5.15 (1B, d, *J* = 147 Hz), −9.7 (4B, *J* = 160 Hz), −11.6 (2B, d, *J* = 158 Hz), −12.4 (2B, d, *J* = 161 Hz). IR (film, cm^−1^): 3012 (ν_C-H_), 2976 (ν_C-H_), 2933 (ν_C-H_), 2603 (br, ν_B-H_), 2580 (br, ν_B-H_), 1439, 1423, 1386, 1289. ESI HRMS: *m*/*z* for C_7_H_15_B_10_SN, calcd. 254.2005 [M + H]^+^, obsd. 254.2008 [M + H]^+^.

Spectral data for **H[3]**. ^1^H NMR (acetone-*d*_6_, ppm): *δ* 8.44 (1H, t, *J* = 4.5 Hz, *H*_py_), 8.01 (1H, m, *H*_py_), 7.45 (1H, m, *H*_py_), 7.27 (1H, m, *H*_py_), 2.15 (1H, br s, C*H*_carb_), 2.9 ÷ (−0.2) (8H, br m, B*H*), −2.54 (1H, br m, B*H*B). ^11^B NMR (acetone-*d*_6_, ppm): *δ* −8.1 (1B, d, *J* = 144 Hz), −9.7 (1B, d, *J* = 137 Hz), −13.6 (1B, *J* = 162 Hz), −15.9 (1B, d, *J* = 144 Hz), −17.0 (1B, d, *J* = 133 Hz), −18.0 (1B, d, *J* = 142 Hz), −21.5 (1B, d, *J* = 153 Hz), −32.0 (1B, dd, *J* = 131, *J* = 31 Hz), −36.1 (1B, d, *J* = 141 Hz).

Spectral data for **Cs[3]**. ^1^H NMR (acetone-*d*_6_, ppm): *δ* 8.30 (1H, d, *J* = 6.2 Hz, *H*(6)_py_), 7.70 (1H, t, *J* = 7.7 Hz, *H*(4)_py_), 7.24 (1H, d, *J* = 7.8 Hz, *H*(3)_py_), 7.02 (1H, t, *J* = 7.3 Hz, *H*(5)_py_), 2.10 (1H, br s, C*H*_carb_), 3.2 ÷ 0.3 (8H, br m, B*H*), −2.42 (1H, br m, B*H*B). ^13^C NMR (acetone-*d*_6_, ppm): *δ* 166.0 (*C*(2)_py_), 148.6 (*C*(6)_py_), 136.5 (*C*(4)_py_), 121.4 (*C*(3)_py_), 118.9 (*C*(5)_py_), 52.6 (*C*_carb_S), 50.4 (*C*_carb_H). ^11^B NMR (acetone-*d*_6_, ppm): *δ* −8.1 (1B, d, *J* = 145 Hz, B(11)), −9.7 (1B, d, *J* = 141 Hz, B(9)), −13.6 (1B, *J* = 158 Hz, B(3)), −16.0 (1B, d, *J* = 132 Hz, B(5)), −17.0 (1B, d, *J* = 138 Hz, B(6)), −17.9 (1B, d, *J* = 130 Hz, B(4)), −21.5 (1B, d, *J* = 149 Hz, B(2)), −32.0 (1B, dd, *J* = 130, *J* = 40 Hz, B(10)), −36.1 (1B, d, *J* = 140 Hz, B(1)). IR (film, cm^−1^): 3010 (ν_C-H_), 2973 (ν_C-H_), 2929 (ν_C-H_), 2350 (br, ν_B-H_), 1441, 1354, 1295. ESI HRMS: *m*/*z* for C_7_H_15_B_9_SN, calcd. 243.1810 [M]^−^, obsd. 243.1815 [M]^−^.

### 3.4. Synthesis of 1-(NC_5_H_4_-2′-CH_2_S)-1,2-C_2_B_10_H_11_ *(**4**)*

2-(Bromomethyl)pyridine hydrobromide (0.54 g, 2.12 mmol) was added to a solution of the trimethylammonium salt of 1-mercapto-*closo*-carborane (0.50 g, 2.12 mmol) in ethanol (20 mL) and the reaction mixture was refluxed for about 5 h, cooled to ambient temperature and evaporated to dryness in vacuum. The obtained product was isolated using column gradient elution chromatography on silica with CH_2_Cl_2_:acetone mixture (from 19:1 to 1:1) as an eluent to give 0.21 g (37% yield) of white crystalline product **3**. ^1^H NMR (acetone-*d*_6_, ppm): *δ* 8.52 (1H, d, *J* = 4.8 Hz, *H*(6)_py_), 7.77 (1H, m, *H*(4)_py_), 7.47 (1H, d, *J* = 7.9 Hz, *H*(3)_py_), 7.30 (1H, m, *H*(5)_py_), 4.93 (1H, br s, C*H*_carb_), 4.42 (2H, s, C*H*_2_), 3.3 ÷ 1.4 (10H, br m, B*H*). ^13^C NMR (acetone-d_6_, ppm): *δ* 155.1 (*C*(2)_py_), 149.7 (*C*(6)_py_), 137.5 (*C*(4)_py_), 123.8 (*C*(3)_py_), 123.2 (*C*(5)_py_), 75.4 (*C*_carb_S), 68.7 (*C*_carb_H), 43.3 (*C*H_2_). ^11^B NMR (acetone-*d*_6_, ppm): *δ* −2.1 (1B, d, *J* = 148 Hz), −5.4 (1B, d, *J* 148 = Hz), −9.4 (4B, *J* = 154 Hz), −11.9 (2B, d, *J* = 181 Hz), −12.4 (2B, d, *J* = 159 Hz). IR (film, cm^−1^): 3071 (ν_C-H_), 3019 (ν_C-H_), 2933 (ν_C-H_), 2600 (br, ν_B-H_), 1594, 1574, 1475, 1439. ESI HRMS: *m*/*z* for C_8_H_17_B_10_SN, calcd. 268.2162 [M + H]^+^, obsd. 268.2155 [M + H]^+^.

### 3.5. Synthesis of Cs[7-(NC_5_H_4_-2′-CH_2_S)-7,8-C_2_B_9_H_11_] (**Cs[5]**)

Compound **4** (0.19 g, 0.71 mmol) was dissolved in ethanol (20 mL) and cesium fluoride (0.22 g, 1.42 mmol) was added. The solution was refluxed for about 20 h. The precipitate formed was filtered off and the filtrate was evaporated under reduced pressure. The residue was dissolved in acetone (15 mL) and unreacted CsF was filtered off. The filtrate was evaporated *in vacuo* to give a white solid of **4** (0.27 g, 98% yield). ^1^H NMR (acetone-*d*_6_, ppm): *δ* 8.46 (1H, d, *J* = 4.2 Hz, *H*(6)_py_), 7.68 (1H, t, *J* = 7.6 Hz, *H*(4)_py_), 7.40 (1H, d, *J* = 7.8 Hz, *H*(3)_py_), 7.17 (1H, m, *H*(5)_py_), 4.13 (1H, d, *J* = 12.8 Hz, C*H*_2_), 3.89 (1H, d, *J* = 12.8 Hz, C*H*_2_), 1.63 (1H, br s, C*H*_carb_), 3.2 ÷ 0.2 (8H, br m, B*H*), −2.57 (1H, br m, B*H*B). ^13^C NMR (acetone-*d*_6_, ppm): *δ* 159.9 (*C*(2)_py_), 149.0 (*C*(6)_py_), 136.2 (*C*(4)_py_), 123.4 (*C*(3)_py_), 121.6 (*C*(5)_py_), 52.3 (*C*_carb_H), 43.2 (*C*H_2_). ^11^B NMR (acetone-d_6_, ppm): *δ* −9.3 (1B, d, *J* = 137 Hz), −10.4 (1B, d, *J* = 135 Hz), −14.9 (1B, *J* = 159 Hz), −17.2 (3B, d, *J* = 139 Hz), −22.1 (1B, d, *J* = 148 Hz), −32.9 (1B, dd, *J* = 124, *J* = 32 Hz), −36.8 (1B, d, *J* = 138 Hz). IR (film, cm^−1^): 3059 (ν_C-H_), 3011 (ν_C-H_), 2972 (ν_C-H_), 2928 (ν_C-H_), 2528 (br, ν_B-H_), 1597, 1570, 1479, 1439, 1253. ESI HRMS: *m*/*z* for C_7_H_15_B_9_SN, calcd. 257.1962 [M]^−^, obsd. 257.1967 [M]^−^.

### 3.6. General Procedure for Synthesis of Metallacarboranes **6**–**11**

To solution of *nido*-carborane derivative **2**, **4** or **5** in anhydrous THF under argon atmosphere, the 3-fold excess of potassium *tert*-butoxide was added. The reaction mixture was stirred at ambient temperature for about 10 min and the 1.1-fold excess of phosphine complexes [(Ph_3_P)_2_MCl_2_] (M=Ni, Pd) was added to one portion. Immediately, a dark brown solution was observed. The resulting mixture was stirred at ambient temperature for about 30 min and the solvent was evaporated to dryness under vacuum conditions. The target complex was isolated using column chromatography on silica using CH_2_Cl_2_ as eluent. If necessary, an additional column chromatography on silica using hexane as eluent was performed to purify the complex from uncoordinated triphenylphosphine.

*3-Ph_3_P-3-(4(7)-NC_5_H_4_-2′-S)-closo-3,1,2-NiC_2_B_9_H_10_* (**6**). The synthesis was carried out using **Cs[1]** (0.15 g, 0.40 mmol), *t*-BuOK (0.13 g, 1.20 mmol), and [(PPh_3_)_2_NiCl_2_] (0.29 g, 0.44 mmol) in THF (15 mL) to give brown solid of **6** (0.08 g, 31% yield). ^1^H NMR (CD_2_Cl_2_, ppm): *δ* 7.79 (7H, m, P*Ph*_3_ + *H*_py_), 7.50 ÷ 7.36 (9H, br m, P*Ph*_3_), 7.21 (1H, d, *J* = 8.5 Hz, *H*_py_), 7.03 (1H, t, *J* = 7.7 Hz, *H*_py_), 6.03 (1H, t, *J* = 6.7 Hz, *H*_py_), 2.77 (1H, br s, C*H*_carb_), 2.44 (1H, br s, C*H*_carb_), 2.6 ÷ 0.1 (8H, br m, B*H*). ^13^C NMR (CD_2_Cl_2_, ppm): *δ* 152.6 (*C*_py_), 135.0 (*C*_py_), 134.23 (d, *J* = 9.7 Hz, Ph), 130.8 (Ph), 128.62 (d, *J* = 9.2 Hz, Ph), 125.0 (*C*_py_), 117.2 (*C*_py_), 33.0 (*C*_carb_H), 31.3 (*C*_carb_H). ^11^B NMR (CD_2_Cl_2_, ppm): *δ* −1.0 (1B, d, *J* = 119 Hz), −8.0 (2B, s + d, *J* = 127 Hz), −10.8 (2B, d, *J* = 124 Hz), −16.4 (1B, d, *J* = 144 Hz), −19.8 (1B, d, *J* = 150 Hz), −23.4 (2B, d, *J* = 138 Hz). ^31^P NMR (CD_2_Cl_2_, ppm): *δ* 31.2 (PPh_3_). IR (film, cm^−1^): 3039 (ν_C-H_), 2992 (ν_C-H_), 2940 (ν_C-H_), 2877 (ν_C-H_), 2556 (br, ν_B-H_), 1471, 1455, 1438, 1392, 1364, 1226, 1194. ESI HRMS: *m*/*z* for C_25_H_29_B_9_NiNPS, calcd. *m*/*z* 563.2079 [M + H]^+^, obsd. *m*/*z* 563.2071 [M + H]^+^. UV (acetone, nm): λ 207, 247, 275, 323.

*3-Ph_3_P-3-(4(7)-NC_5_H_4_-2′-S)-closo-3,1,2-PdC_2_B_9_H_10_* (**7**). The synthesis was carried out using **Cs[1]** (0.15 g, 0.40 mmol), *t*-BuOK (0.13 g, 1.20 mmol), and [(PPh_3_)_2_PdCl_2_] (0.31 g, 0.44 mmol) in THF (15 mL) to give dark brown solid of **7** (0.06 g, 25% yield). ^1^H NMR (acetone-*d*_6_, ppm): *δ* 7.80 (6H, m, P*Ph*_3_), 7.60 ÷ 7.47 (10H, br m, P*Ph*_3_ + *H*_py_), 7.38 (2H, *H*_py_), 6.40 (1H, m, *H*_py_), 4.48 (1H, br s, C*H*_carb_), 3.75 (1H, br s, C*H*_carb_), 3.4 ÷ (−0.5) (8H, br m, B*H*). ^13^C NMR (acetone-*d*_6_, ppm): *δ* 152.5 (*C*_py_), 136.3 (*C*_py_), 134.30 (d, *J* = 12.6 Hz, Ph), 131.1 (Ph), 128.73 (d, *J* = 10.3 Hz, Ph), 124.6 (*C*_py_), 117.1 (*C*_py_), 55.0 (*C*_carb_H). ^11^B NMR (acetone-*d*_6_, ppm): *δ* 18.5 (1B, d, *J* = 136 Hz), −5.9 (2B, d, *J* = 146 Hz), −9.0 (2B, s + d, *J* = 142 Hz), −10.8 (2B, d, *J* = 144 Hz), −22.0 (1B, d, *J* = 140 Hz), −25.1 (1B, d, *J* = 131 Hz). ^31^P NMR (acetone-*d*_6_, ppm): *δ* 32.1 (PPh_3_). IR (film, cm^−1^): 2964 (ν_C-H_), 2933 (ν_C-H_), 2857 (ν_C-H_), 2560 (br, ν_B-H_), 1594, 1483, 1459, 1435, 1415, 1249. ESI HRMS: *m*/*z* for C_25_H_29_B_9_PdNPS, calcd. *m*/*z* 610.1782 [M + H]^+^, obsd. *m*/*z* 610.1771 [M + H]^+^. UV (acetone, nm): λ 206, 247, 269, 327.

*3-Ph_3_P-3-(1(2)-NC_5_H_4_-2′-S)-closo-3,1,2-NiC_2_B_9_H_10_* (**8**). The synthesis was carried out using **Cs[3]** (0.20 g, 0.58 mmol), *t*-BuOK (0.19 g, 1.74 mmol), and [(PPh_3_)_2_NiCl_2_] (0.42 g, 0.64 mmol) in THF (15 mL) to give brown solid of **8** (0.07 g, 22% yield). ^1^H NMR (acetone-*d*_6_, ppm): *δ* 8.04 (6H, m, P*Ph*_3_), 7.73 ÷ 7.43 (10H, br m, P*Ph*_3_ + *H*_py_), 7.39 (1H, m, *H*_py_), 7.29 (1H, m, *H*_py_), 6.45 (1H, m, *H*_py_), 3.44 (1H, br s, C*H*_carb_), 2.7 ÷ 0.5 (9H, br m, B*H*). ^13^C NMR (acetone-*d*_6_, ppm): *δ* 151.7 (*C*_py_), 136.4 (*C*_py_), 135.05 (d, *J* = 10.0 Hz, Ph), 134.2, 131.8 (Ph), 131.1 (Ph), 130.3, 128.48 (d, *J* = 10.0 Hz, Ph), 120.3 (*C*_py_), 119.0 (*C*_py_). ^11^B NMR (acetone-*d*_6_, ppm): *δ* −2.5 (1B, d, *J* = 120 Hz), −7.0 (1B, d, *J* = 138 Hz), −10.8 (1B, d, *J* = 143 Hz), −11.8 (1B, d, *J* = 120 Hz), −14.6 (2B, d, *J* = 126 Hz), −18.1 (2B, d, *J* = 135 Hz), −20.7 (1B, d, *J* = 153 Hz). ^31^P NMR (acetone-*d*_6_, ppm): *δ* 43.7 (PPh_3_). IR (film, cm^−1^): 3084 (ν_C-H_), 3062 (ν_C-H_), 2968 (ν_C-H_), 2931 (ν_C-H_), 2860 (ν_C-H_), 2559 (br, ν_B-H_), 1598, 1440, 1421, 1364. ESI HRMS: *m*/*z* for C_25_H_29_B_9_NiNPS, calcd. *m*/*z* 563.2079 [M + H]^+^, obsd. *m*/*z* 563.2064 [M + H]^+^. UV (acetone, nm): λ 213, 325, 397.

*3-Ph_3_P-3-(1(2)-NC_5_H_4_-2′-S)-closo-3,1,2-PdC_2_B_9_H_10_* (**9**). The synthesis was carried out using **Cs[3]** (0.19 g, 0.51 mmol), *t*-BuOK (0.17 g, 1.53 mmol), and [(PPh_3_)_2_PdCl_2_] (0.39 g, 0.56 mmol) in THF (15 mL) to give dark brown solid of **9** (0.10 g, 33% yield). ^1^H NMR (acetone-*d*_6_, ppm): *δ* 7.88 (6H, m, P*Ph*_3_), 7.61 ÷ 7.47 (10H, br m, P*Ph*_3_ + *H*_py_), 7.40 (1H, m, *H*_py_), 7.36 (1H, m, *H*_py_), 6.67 (1H, t, *J* = 6.4 Hz*, H*_py_), 3.85 (1H, br s, C*H*_carb_), 3.6 ÷ (−0.1) (9H, br m, B*H*). ^13^C NMR (acetone-*d*_6_, ppm): *δ* 150.2 (*C*_py_), 137.1 (*C*_py_), 134.87 (d, *J* = 11.7 Hz, Ph), 134.0, 131.5 (Ph), 129.8, 128.89 (d, *J* = 10.9 Hz, Ph), 120.4 (*C*_py_), 119.6 (*C*_py_), 39.4 (*C*_carb_H). ^11^B NMR (acetone-*d*_6_, ppm): *δ* 1.9 (1B, d, *J* = 138 Hz), −3.4 (1B, d, *J* = 121 Hz), −6.3 (1B, d, *J* = 124 Hz), −12.7 (1B, d, *J* = 132 Hz), −13.7 (3B, d, *J* = 157 Hz), −22.4 (1B, d, *J* = 147 Hz), −25.3 (1B, d, *J* = 133 Hz). ^31^P NMR (acetone-*d*_6_, ppm): *δ* 45.4 (PPh_3_). IR (film, cm^−1^): 3091 (ν_C-H_), 2965 (ν_C-H_), 2932 (ν_C-H_), 2861 (ν_C-H_), 2554 (br, ν_B-H_), 1595, 1442, 1250. ESI HRMS: *m*/*z* for C_25_H_29_B_9_PdNPS, calcd. *m*/*z* 628.2037 [M + NH_4_]^+^, obsd. *m*/*z* 628.1990 [M + NH_4_]^+^; calcd. *m*/*z* 632.1601 [M + Na]^+^, obsd. *m*/*z* 632.1636 [M + NH_4_]^+^. UV (acetone, nm): λ 207, 215, 323.

*3-Ph_3_P-3-(1(2)-NC_5_H_4_-2′-CH_2_S)-closo-3,1,2-NiC_2_B_9_H_10_* (**10**). The synthesis was carried out using **Cs[5]** (0.12 g, 0.31 mmol), *t*-BuOK (0.10 g, 0.93 mmol), and [(PPh_3_)_2_NiCl_2_] (0.22 g, 0.34 mmol) in THF (15 mL) to give brown solid of **10** (0.06 g, 34% yield). ^1^H NMR (DMSO-*d*_6_, ppm): *δ* 8.76 (1H, d, *J* = 5.3 Hz, *H*_py_), 7.73 (1H, t, *J* = 7.7 Hz, *H*_py_), 7.59 (1H, m, *H*_py_), 7.54 ÷ 7.40 (8H, br m, P*Ph*_3_), 7.40 ÷ 7.29 (7H, br m, P*Ph*_3_), 7.10 (1H, t, *J* = 6.6 Hz, *H*_py_), 4.63 (1H, d, *J* = 13.6 Hz, C*H*_2_), 4.23 (1H, d, *J* = 13.6 Hz, C*H*_2_), 2.3 ÷ 0.9 (9H, br m, B*H*). ^13^C NMR (CDCl_3_, ppm): *δ* 152.7 (*C*_py_), 139.3 (*C*_py_), 133.8 (d, *J* = 8.9 Hz, Ph), 130.5 (Ph), 128.4 (d, *J* = 11.6 Hz, Ph + *C*_py_), 123.4 (*C*_py_). ^11^B NMR (CDCl_3_, ppm): *δ* −7.7 (1B, d, *J* = 158 Hz), −9.8 (3B), −13.3 (2B, d, *J* = 130 Hz), −17.6 (2B), −22.5 (1B). ^31^P NMR (CDCl_3_, ppm): *δ* 37.4 (PPh_3_). IR (film, cm^−1^): 2965 (ν_C-H_), 2928 (ν_C-H_), 2857 (ν_C-H_), 2544 (br, ν_B-H_), 1609, 1483, 1439, 1356, 1186, 1158, 1122. ESI HRMS: *m*/*z* for C_26_H_31_B_9_NiNPS, calcd. *m*/*z* 594.2501 [M + NH_4_]^+^, obsd. *m*/*z* 594.2543 [M + NH_4_]^+^. UV (acetone, nm): λ 206, 243, 291, 323.

*3-Ph_3_P-3-(1(2)-NC_5_H_4_-2′-CH_2_S)-closo-3,1,2-PdC_2_B_9_H_10_* (**11**). The synthesis was carried out using **Cs[5]** (0.23 g, 0.59 mmol), *t*-BuOK (0.20 g, 1.77 mmol), and [(PPh_3_)_2_PdCl_2_] (0.46 g, 0.65 mmol) in THF (15 mL) to give dark brown solid of **9** (0.10 g, 27% yield). ^1^H NMR (CD_2_Cl_2_, ppm): *δ* 8.12 (1H, m, *H*_py_), 7.64 (1H, t, *J* = 7.9 Hz, *H*_py_), 7.55 ÷ 7.37 (9H, br m, P*Ph*_3_ + *H*_py_), 7.36 ÷ 7.27 (7H, br m, P*Ph*_3_), 6.82 (1H, t, *J* = 6.6 Hz, *H*_py_), 4.59 (1H, m, C*H*_2_), 4.03 (1H, m, C*H*_2_), 2.5 ÷ 0.5 (9H, br m, B*H*). ^13^C NMR (CD_2_Cl_2_, ppm): *δ* 152.1 (*C*_py_), 139.2 (*C*_py_), 134.0 (d, *J* = 8.6 Hz, Ph), 130.8 (Ph), 128.4 (d, *J* = 11.6 Hz, Ph), 123.6 (*C*_py_). ^11^B NMR (CD_2_Cl_2_, ppm): *δ* −4.5 (1B, d, *J* = 149 Hz), −6.6 (1B, d, *J* = 119 Hz), −9.5 (1B, d, *J* = 143 Hz), −10.7 (1B, d, *J* = 140 Hz), −14.2 (2B), −15.0 (2B), −24.6 (1B). ^31^P NMR (CD_2_Cl_2_, ppm): *δ* 47.6 (PPh_3_). IR (film, cm^−1^): 2968 (ν_C-H_), 2928 (ν_C-H_), 2861 (ν_C-H_), 2552 (br, ν_B-H_), 1602, 1487, 1435, 1399, 1312, 1190. ESI HRMS: *m*/*z* for C_26_H_31_B_9_PdNPS, calcd. *m*/*z* 642.2194 [M + NH_4_]^+^, obsd. *m*/*z* 642.2190 [M + NH_4_]^+^. UV (acetone, nm): λ 207, 251, 325.

### 3.7. Single Crystal X-ray Diffraction Study

Single crystal X-ray diffraction experiments for **2**·HBr, **4**, **Cs[5]**·0.5Me_2_CO, **8**, and **10** were carried out using SMART APEX2 CCD diffractometer (λ(Mo-Kα) = 0.71073 Å, graphite monochromator, ω-scans) at 140 K (See Supplementary Materials). Collected data were processed using the SAINT and SADABS programs incorporated into the APEX2 program package [83]. The structure was solved using the direct methods and refined using the full-matrix least-squares procedure against F^2^ in anisotropic approximation. The refinement was carried out with the SHELXTL program [84]. The CCDC numbers (2294563 for **2**·HBr, 2294561 for **4**, 2294562 for **Cs[5]**·0.5Me_2_CO, 2294564 for **8**, and 2294565 for **10**) contain the Supplementary Materials for this paper. These data can be obtained free of charge via www.ccdc.cam.ac.uk/data_request/cif (accessed on 9 October 2023).

## 4. Conclusions

A series of *C*- and *B*-substituted *nido*-carborane derivatives with a pyridyl pendant group was prepared. The obtained compounds were used as ligands in the complexation with [(Ph_3_P)_2_NiCl_2_] and [(Ph_3_P)_2_PdCl_2_] to give the corresponding η^5^:κ^1^(*N*)-coordinated complexes of nickel(II) and palladium(II), 3-Ph_3_P-3-(4(7)-NC_5_H_4_-2′-S)-*closo*-3,1,2-MC_2_B_9_H_10_, 3-Ph_3_P-3-(1(2)-NC_5_H_4_-2′-S)-*closo*-3,1,2-MC_2_B_9_H_10_, and 3-Ph_3_P-3-(1(2)-NC_5_H_4_-2′-CH_2_S)-*closo*-3,1,2-MC_2_B_9_H_10_ (M=Ni, Pd). The study of the catalytic activity of the obtained complexes is in progress.

## Data Availability

Crystallographic data are available from CCDC, UK, see Supplementary Material for details.

## Supplementary Materials

The following supporting information can be downloaded at: https://www.mdpi.com/article/10.3390/ijms242015069/s1, The NMR spectra of compounds **1**–**11** and crystallographic data on compounds **2**, **4**, **5**, **8**, and **10**.
