# Supplementary material for: Nickel(II) and Palladium(II) Complexes with η5:κ1(N)-Coordinated Dicarbollide Ligands Containing Pendant Pyridine Group
Source: Int J Mol Sci. 2023 Oct 11;24(20):15069. doi: 10.3390/ijms242015069 (PMC10606403; doi:10.3390/ijms242015069)
Supplement: Supplementary file 1 [file ijms-24-15069-s001.zip › ijms-2637531-supplementary.pdf]

Supplementary Materials

Nickel(II) and Palladium(II) Complexes with η^5:κ^1(*N*)-Coordi- nated Dicarbollide Ligands Containing Pendant Pyridine Group

Dmitriy K. Semyonov [1,2], Marina Yu. Stogniy [1*], Sergey A. Anufriev [1], Sergey V. Timofeev [1], Kyrill Yu. Suponitsky [1,3] and Igor B. Sivaev [1]

[1] A.N. Nesmeyanov Institute of Organoelement Compounds, Russian Academy of Sciences, 28 Vavilov Str., 119991, Moscow, Russia; dksemyonov@mail.ru (D.K.S.); stogniy@ineos.ac.ru (M.Y.S.); trueman476@mail.ru (S.A.A.); timofeev@ineos.ac.ru (S.V.T.); kirshik@yahoo.com (K.Y.S.); sivaev@ineos.ac.ru (I.B.S.)

[2] M.V. Lomonosov Institute of Fine Chemical Technology, MIREA - Russian Technological University, 86 Vernadsky Av., 119571, Moscow, Russia

[3] Basic Department of Chemistry of Innovative Materials and Technologies, G.V. Plekhanov Russian University of Economics, 36 Stremyannyi Line, 117997, Moscow, Russia

* Correspondence: stogniy@ineos.ac.ru

Spectral data for Cs[9-(NC_5H_4-2’-S)-7,8-$C_2B_9H_{11}$] (**1**)

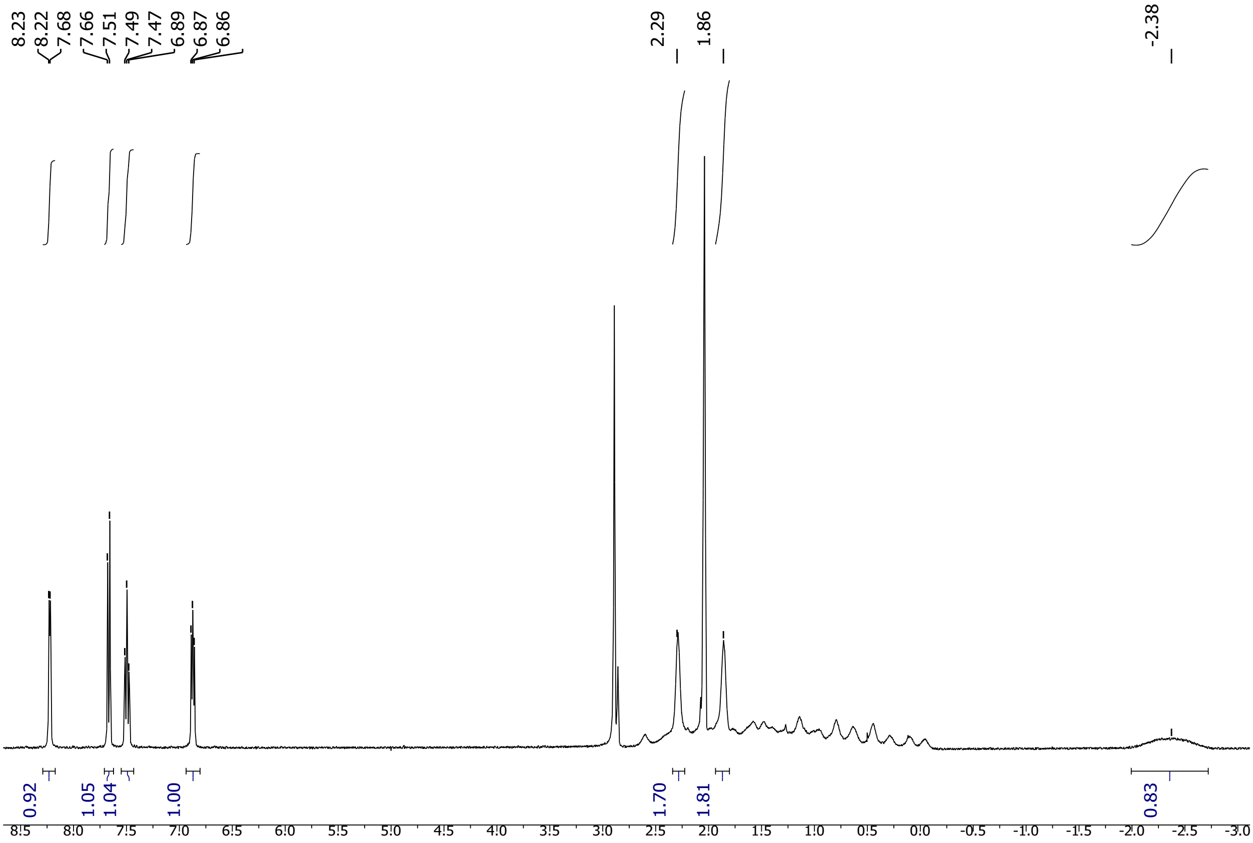

Figure S1. ^{1}H NMR spectrum of **1** in acetone-d_6

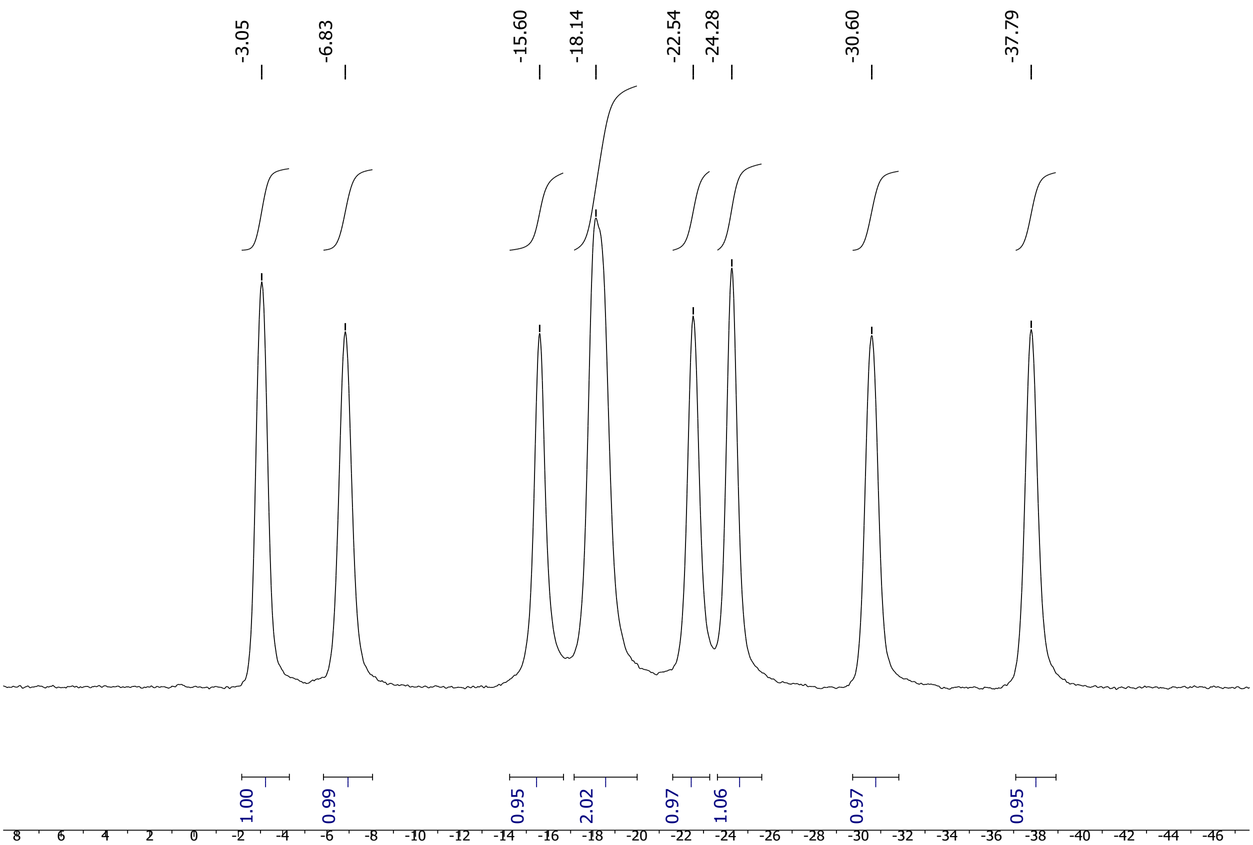

Figure S2. $^{11}B\{^{1}H\}$ NMR spectrum of **1** in acetone-d_6

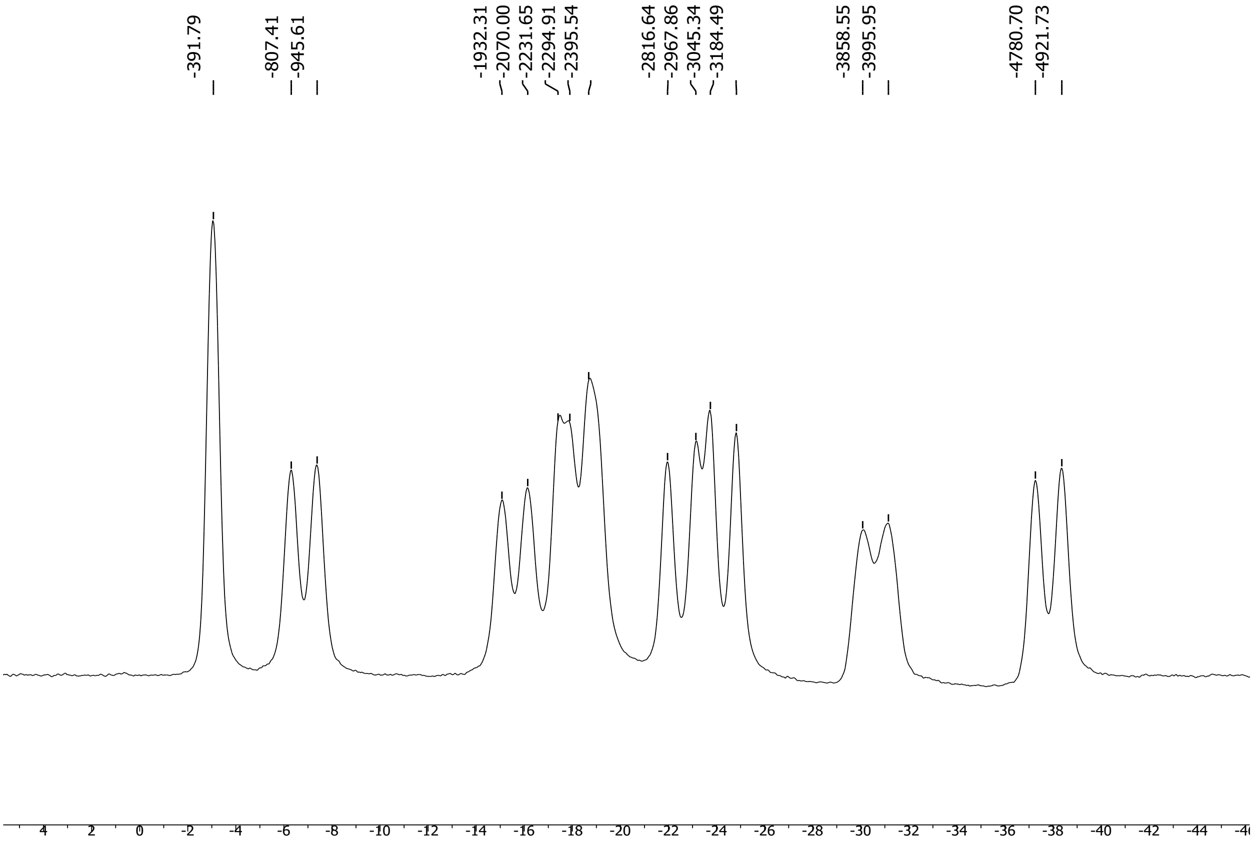

Figure S3. ^{11}B NMR spectrum of **1** in acetone-d_6

Spectral data for 1-(NC_5H_4-2'-S)-1,2-$C_2B_{10}H_{11}$ (**2**)

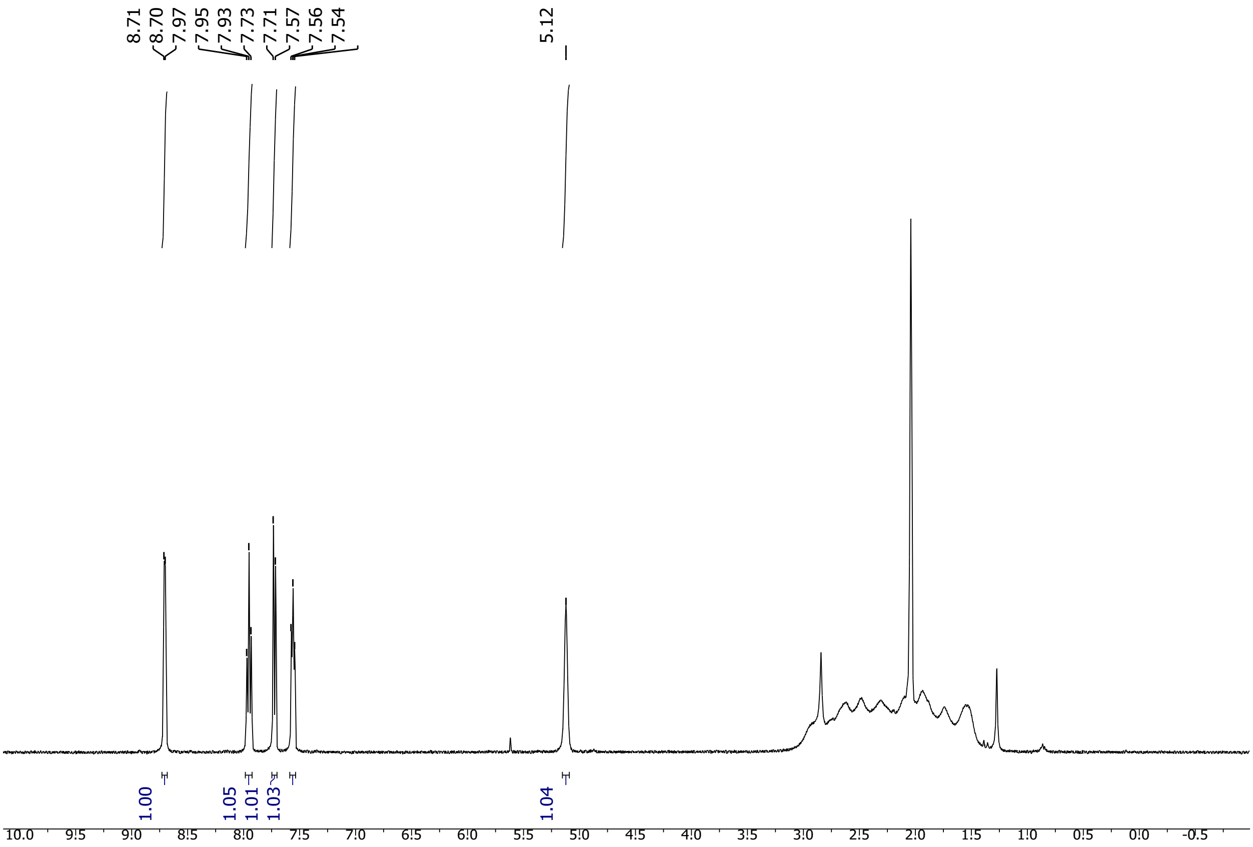

Figure S4. ^{1}H NMR spectrum of **2** in acetone-d_6

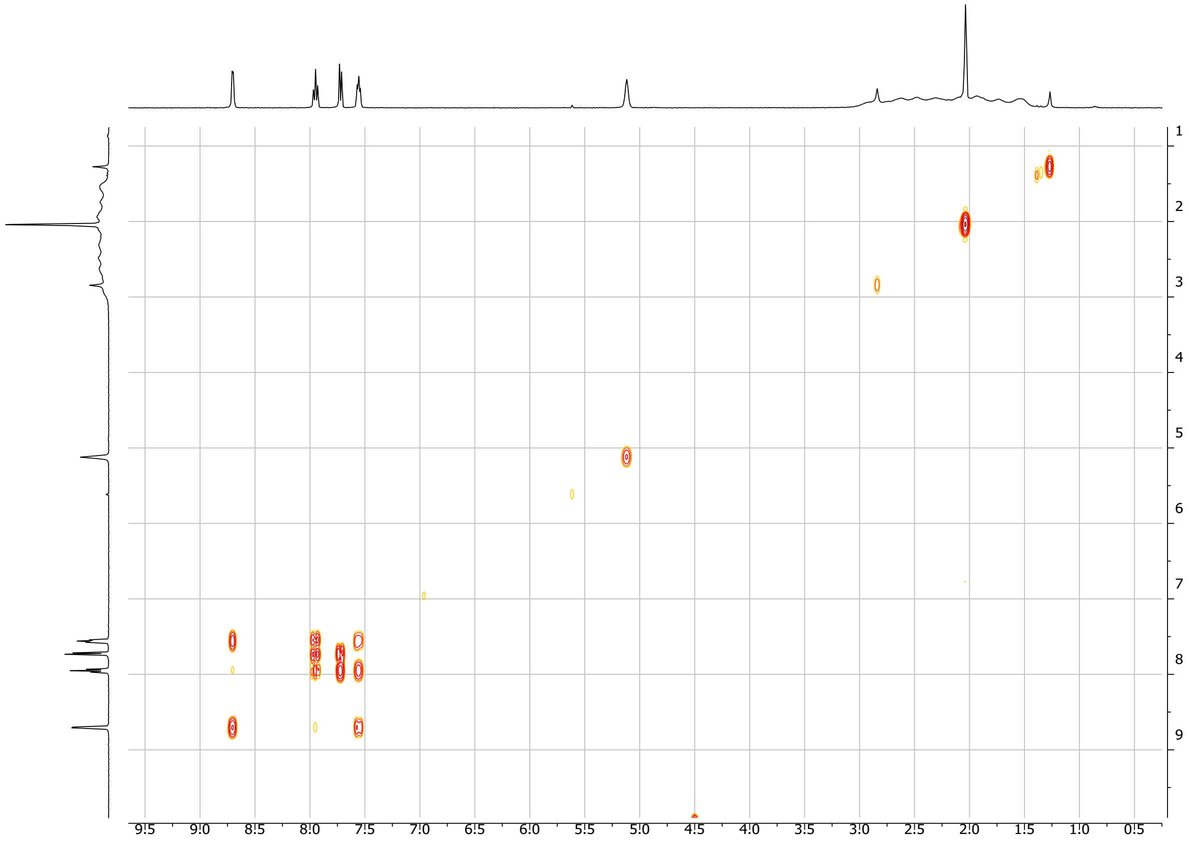

Figure S5. (HH) COSY NMR spectrum of **2** in acetone-d_6

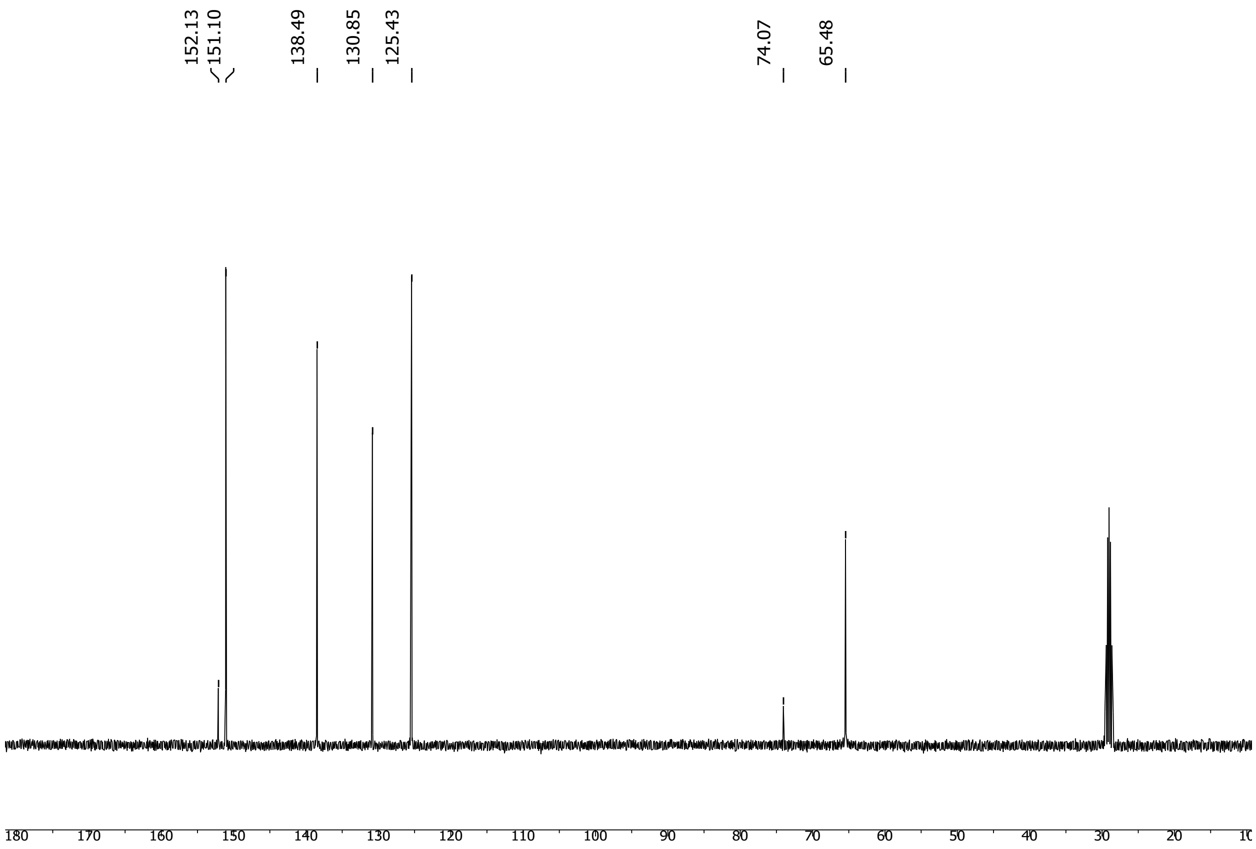

Figure S6. ^{13}C NMR spectrum of **2** in acetone-d_6

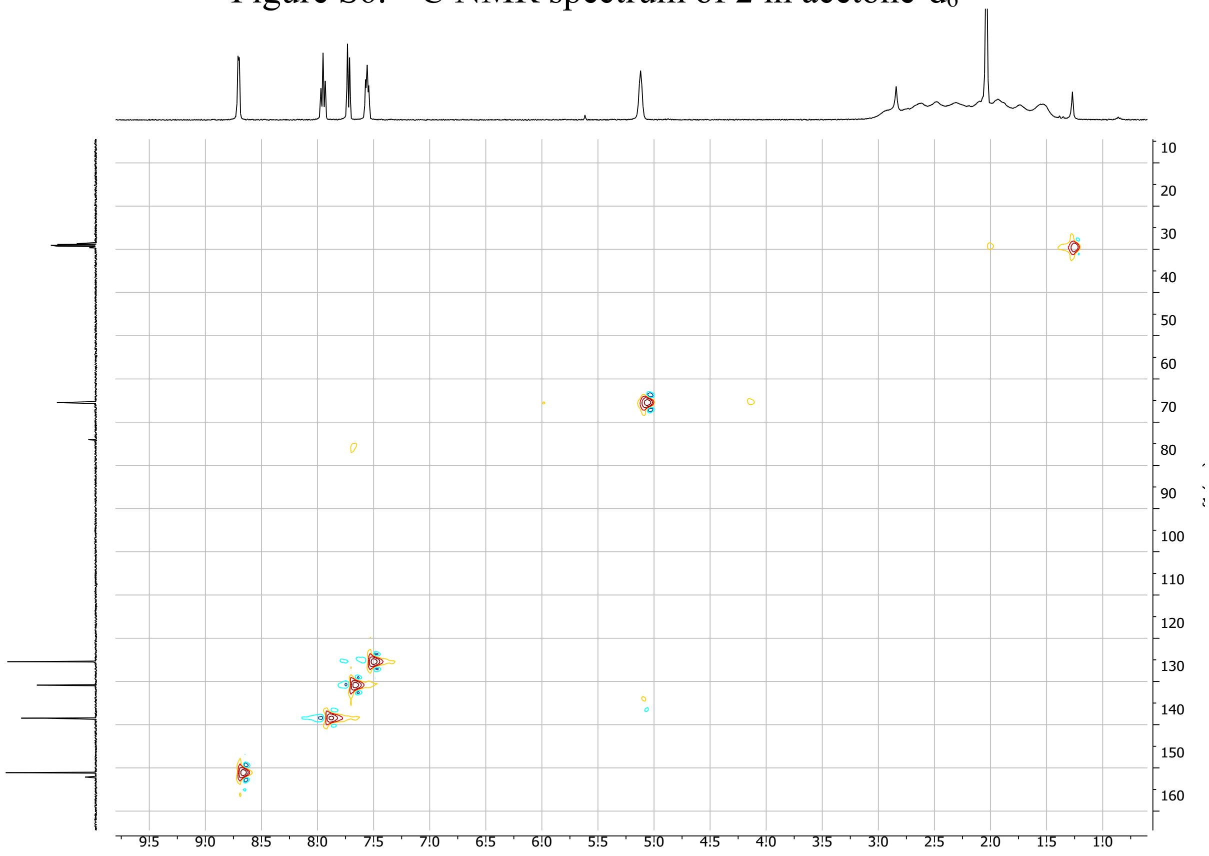

Figure S7. (HC) HSQC NMR spectrum of **2** in acetone-d_6

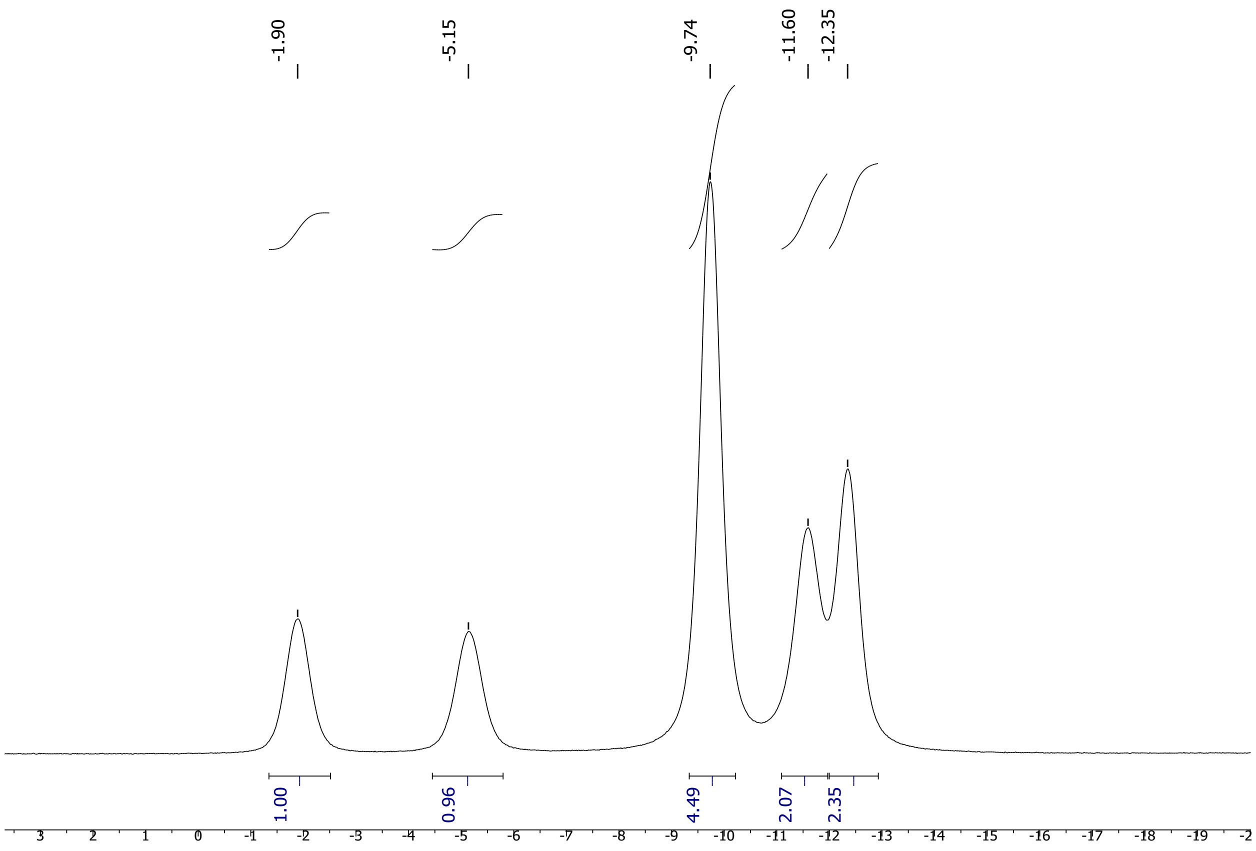

Figure S8. $^{11}B\{^{1}H\}$ NMR spectrum of **2** in acetone-d_6

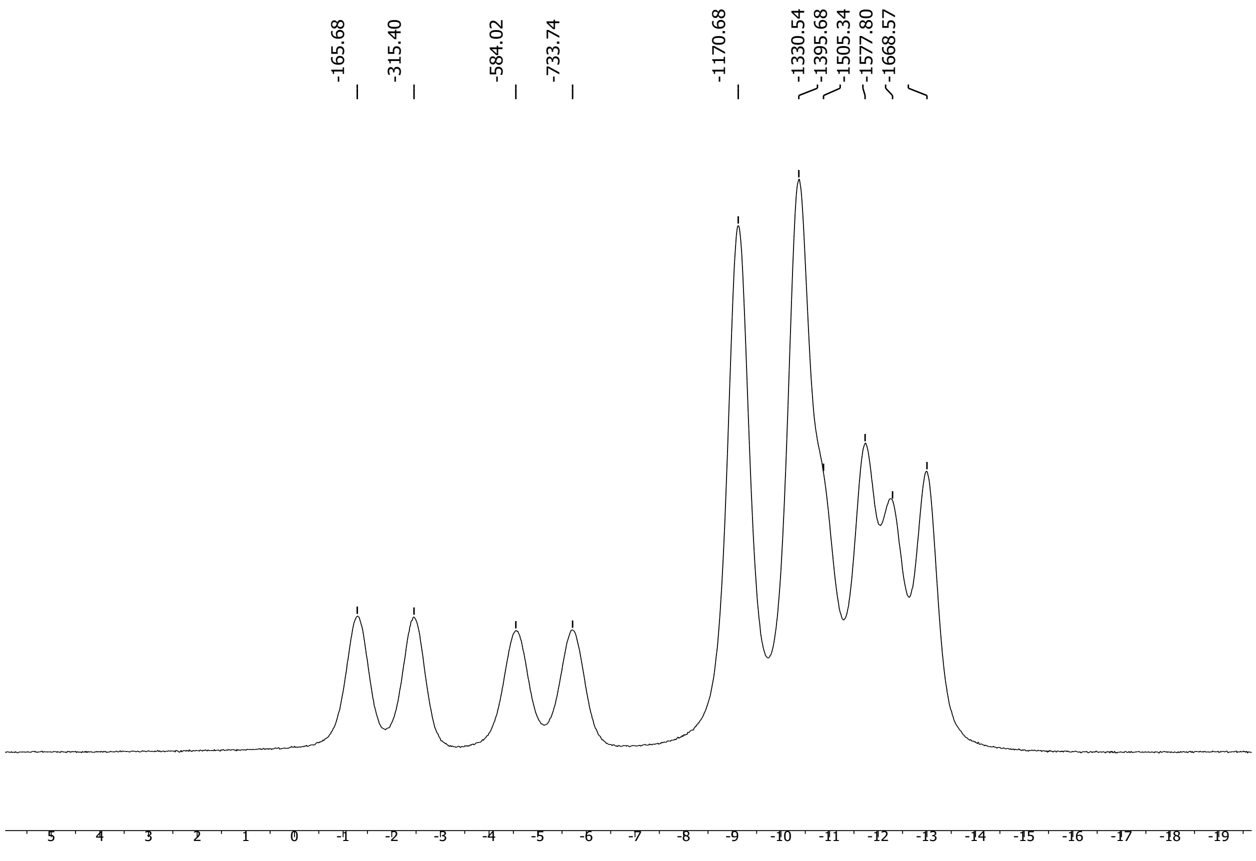

Figure S9. ^{11}B NMR spectrum of **2** in acetone-d_6

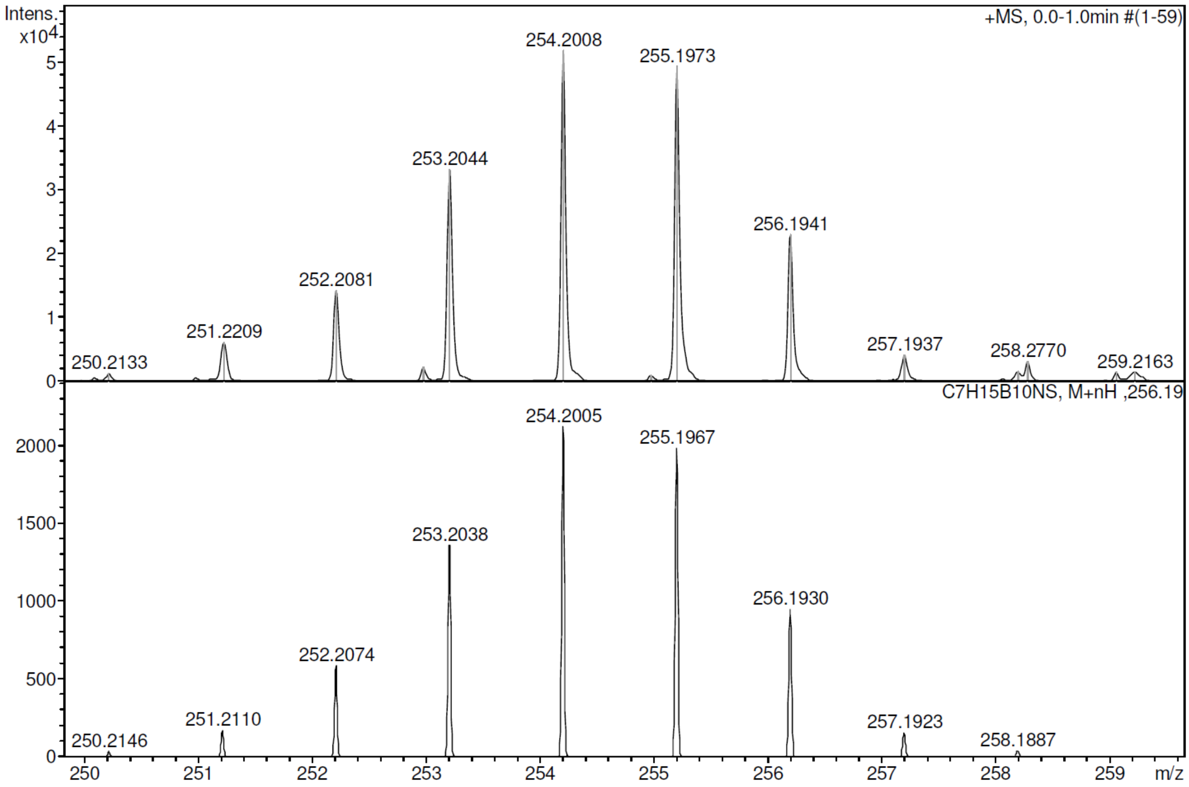

Figure S10. HRMS spectrum of **2**

Spectral data for [7-(HNC_5H_4-2'-S)-7,8-$C_2B_9H_{11}$] (**H[3]**)

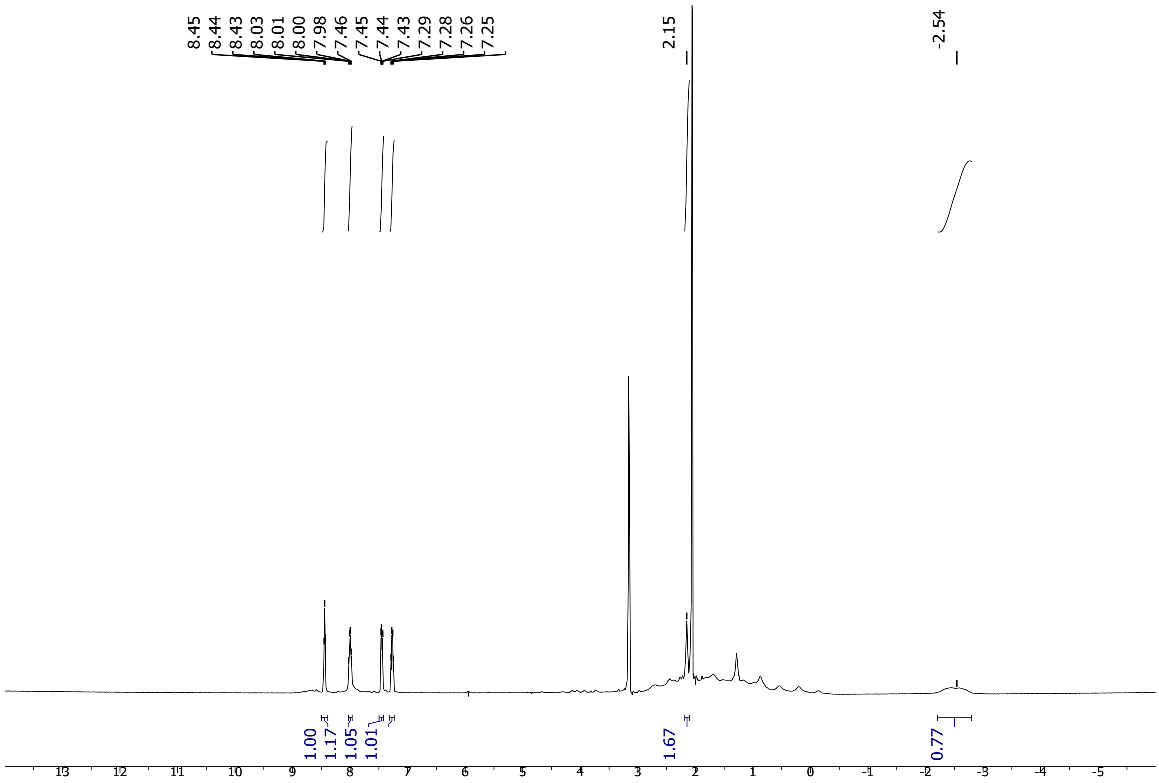

Figure S11. ^{1}H NMR spectrum of **H[3]** in acetone-d_6

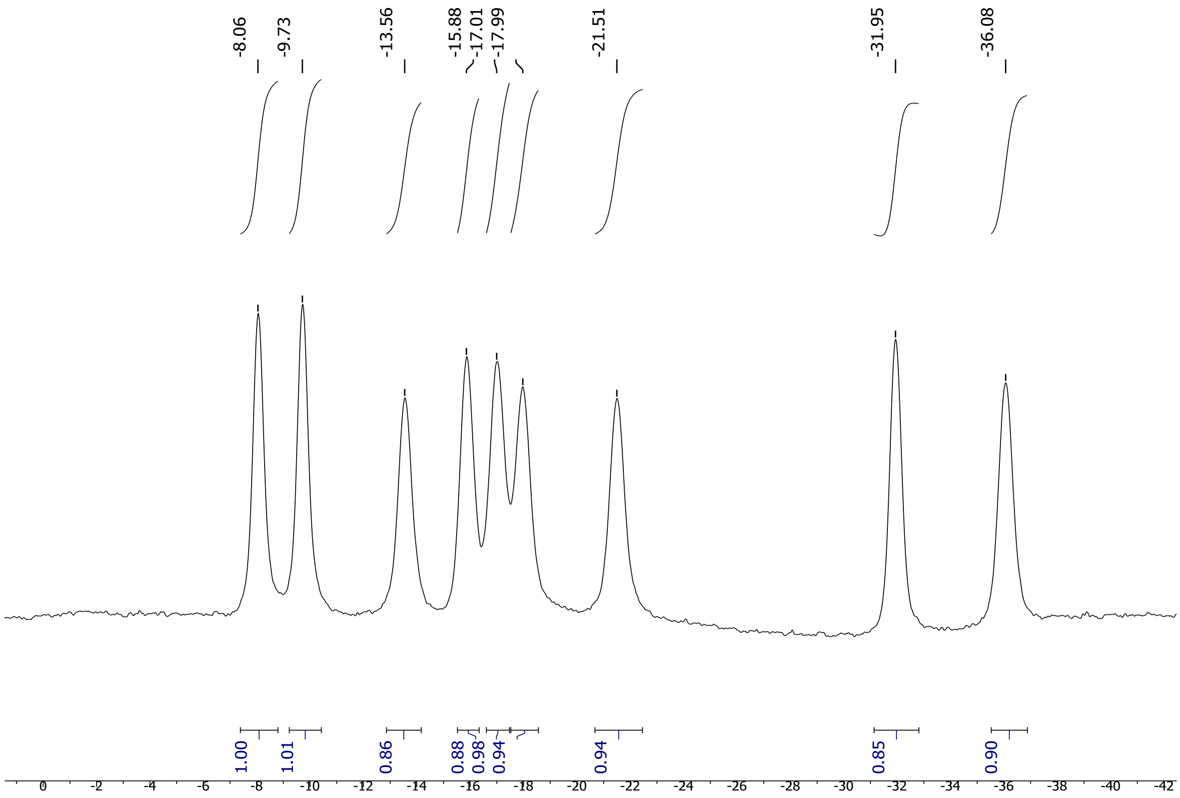

Figure S12. $^{11}B\{^{1}H\}$ NMR spectrum of **H[3]** in acetone-d_6

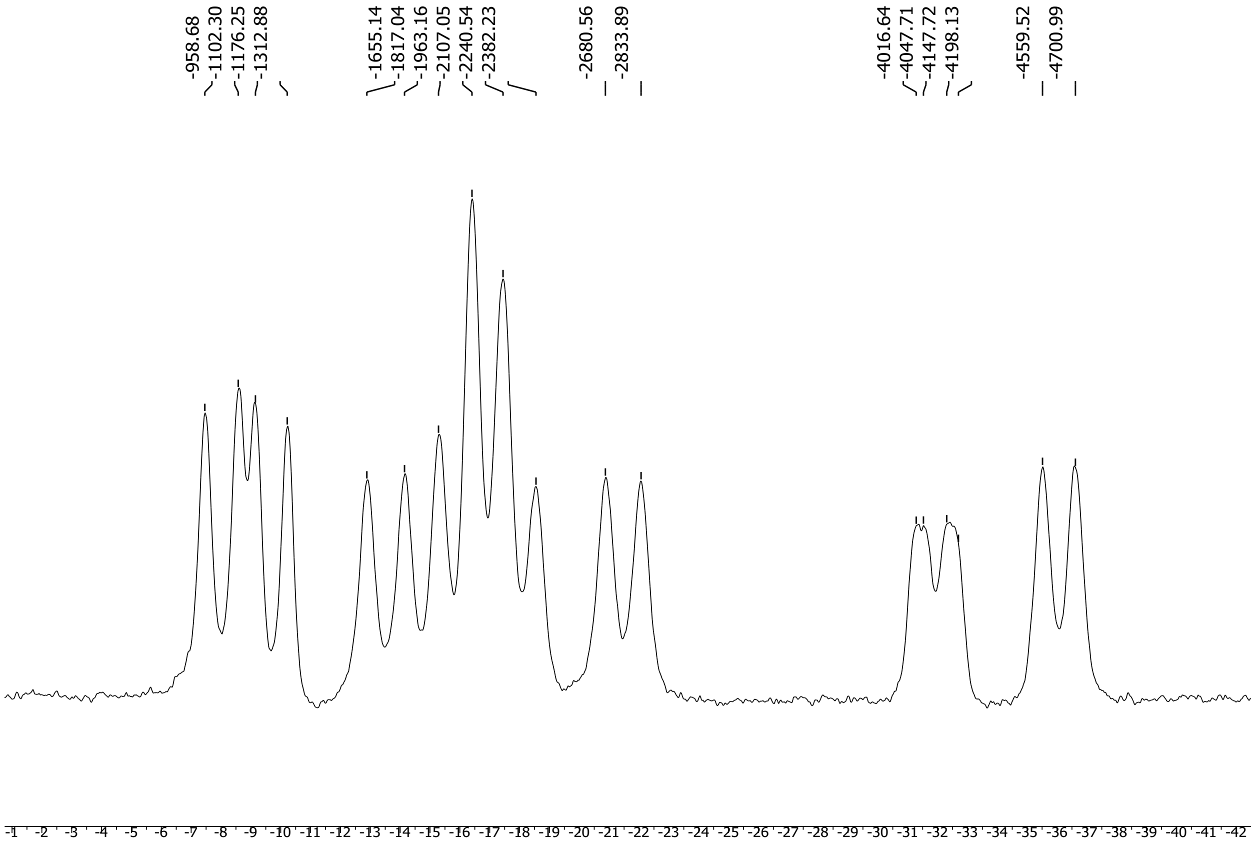

Figure S13. ^{11}B NMR spectrum of **H[3]** in acetone-d_6

Spectral data for Cs[7-(NC_5H_4-2'-S)-7,8-$C_2B_9H_{11}$] (**Cs[3]**)

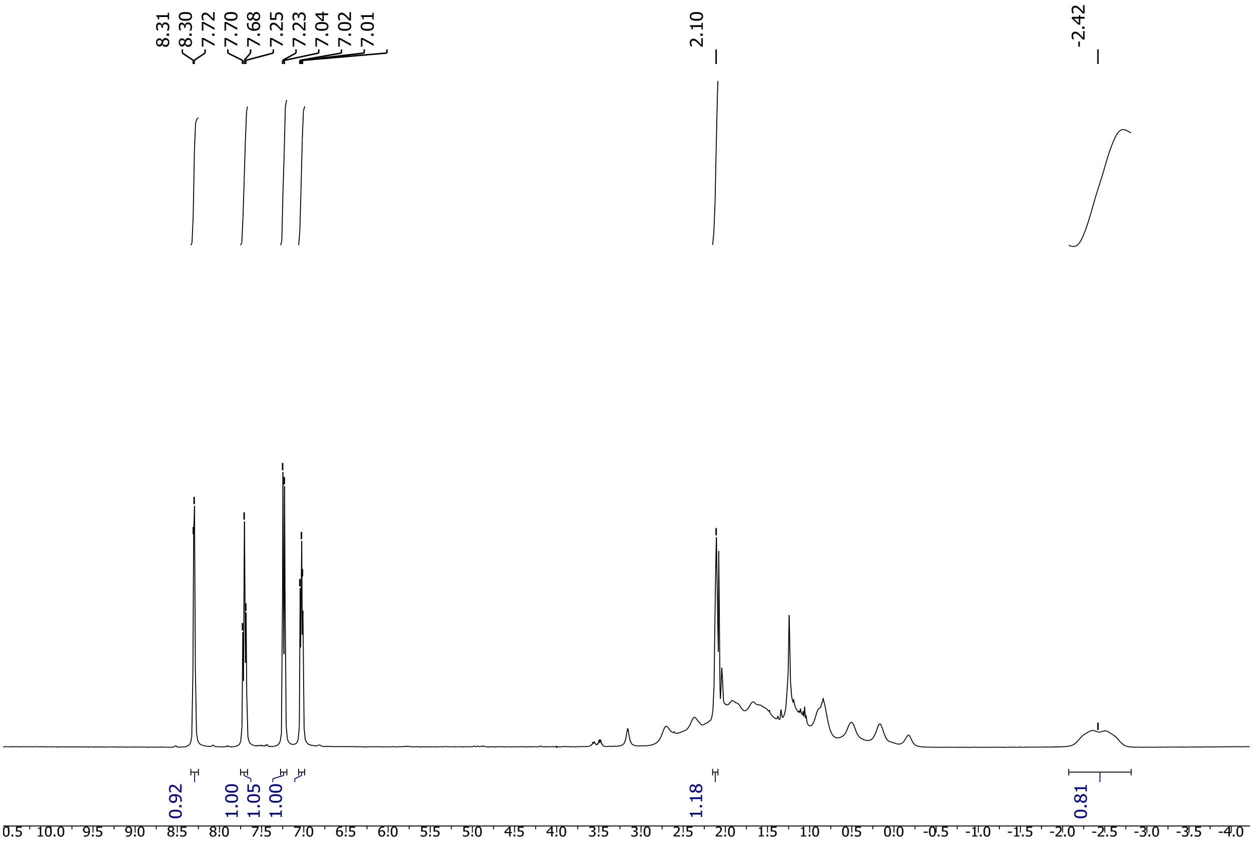

Figure S14. ¹H NMR spectrum of **Cs[3]** in acetone-d_6

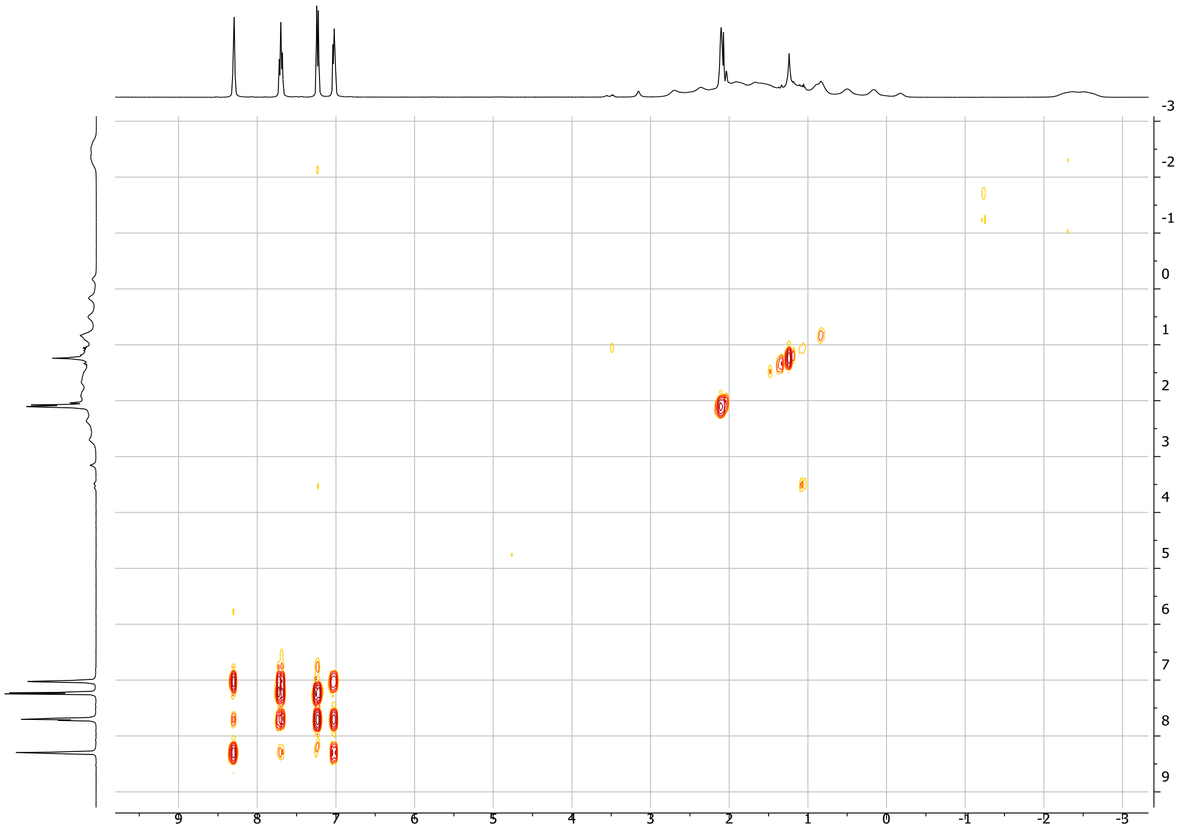

Figure S15. (HH) COSY NMR spectrum of **Cs[3]** in acetone-d_6

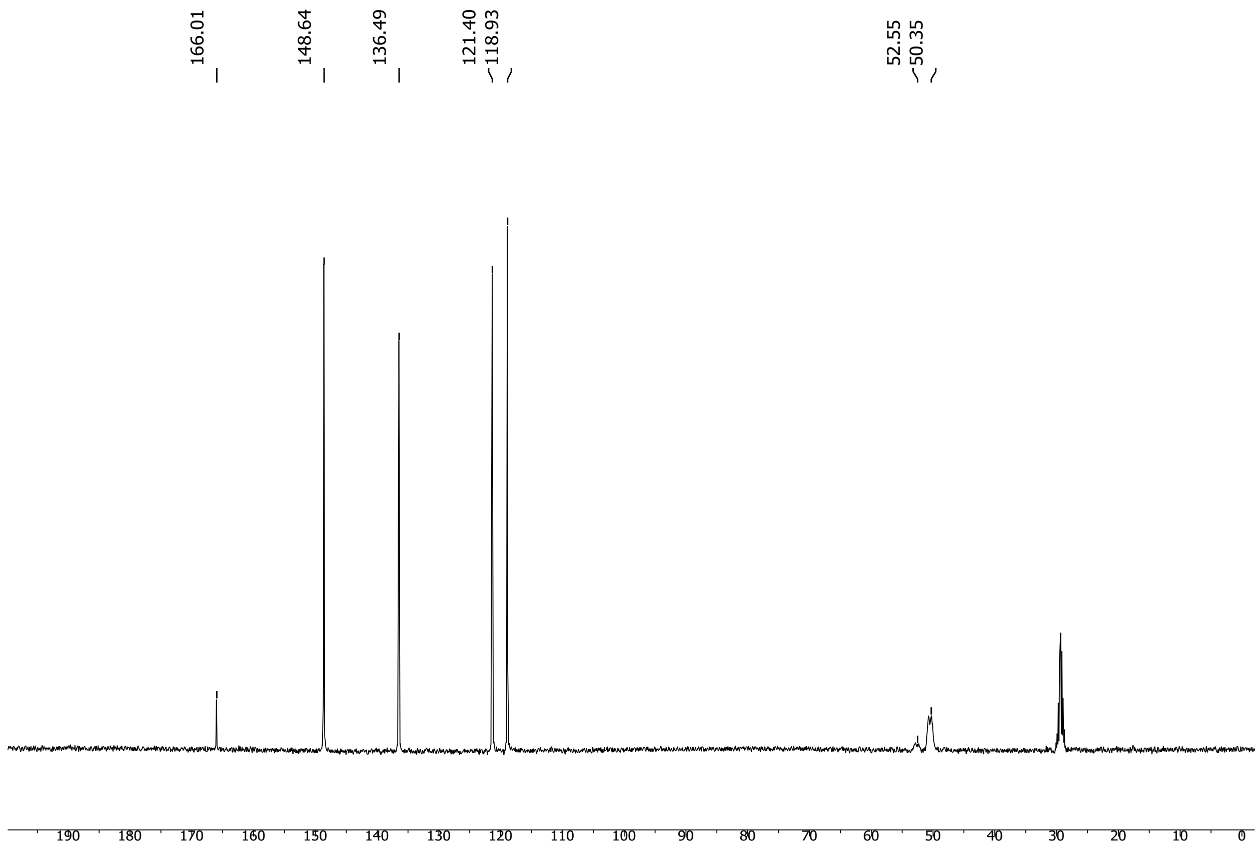

Figure S16. ^{13}C NMR spectrum of **Cs[3]** in acetone-d$_6$

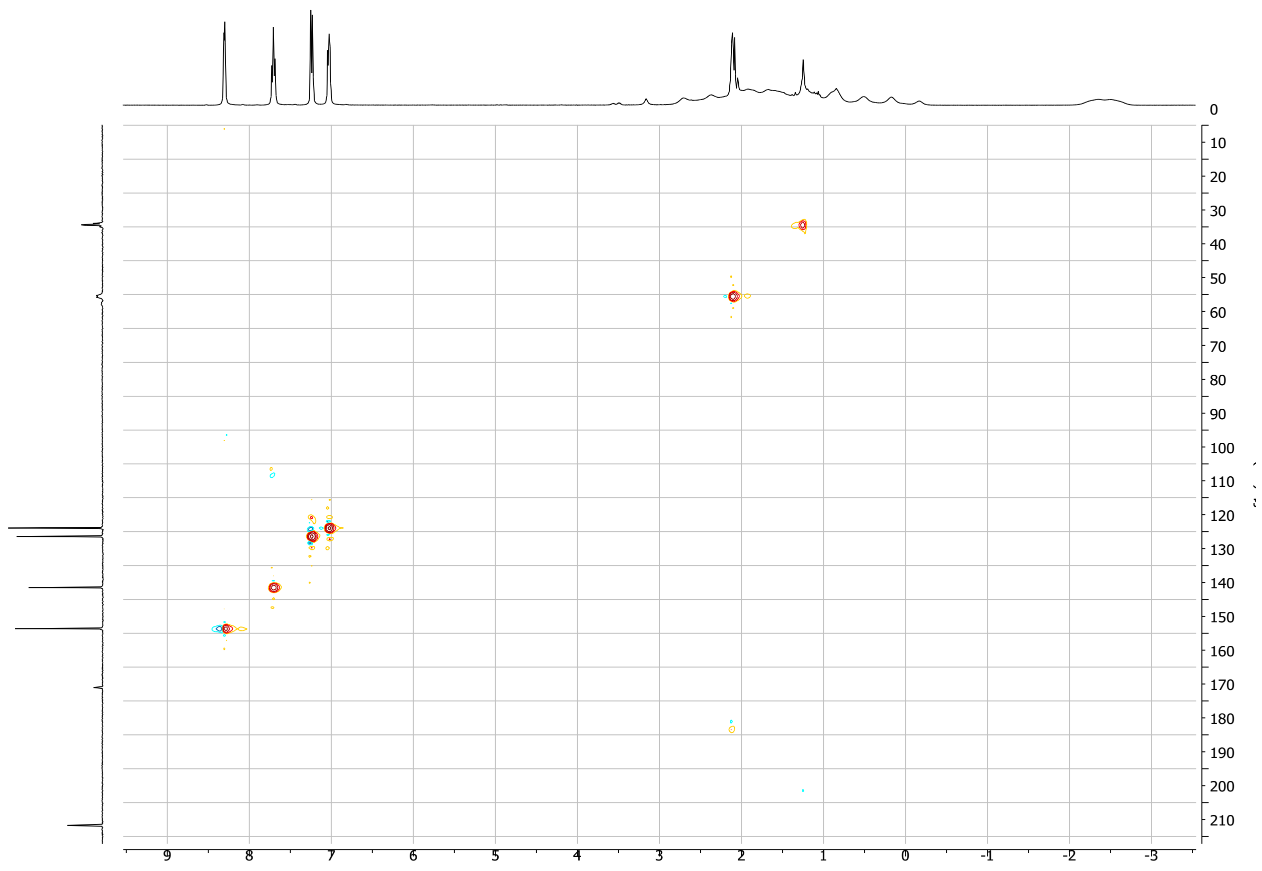

Figure S17. (HC) HSQC NMR spectrum of **Cs[3]** in acetone-d$_6$

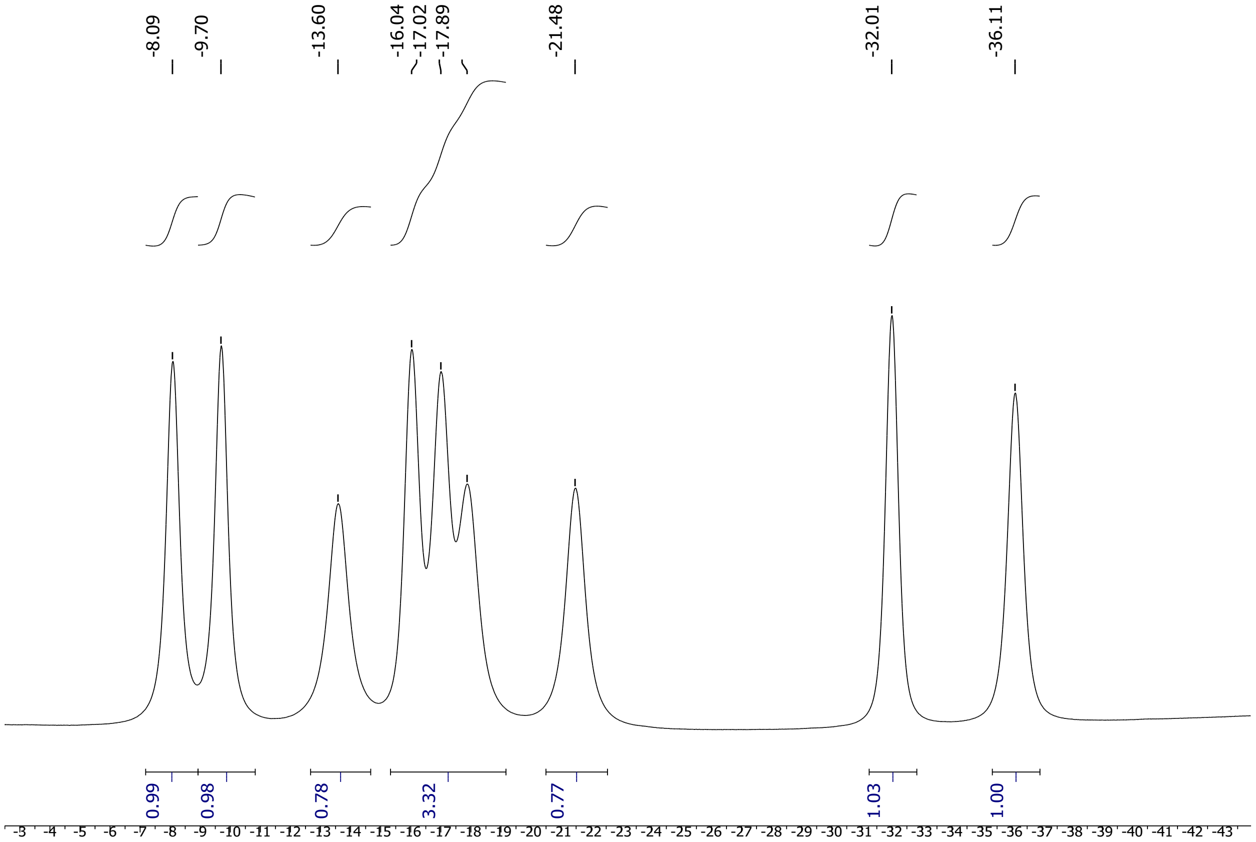

Figure S18. $^{11}B\{^{1}H\}$ NMR spectrum of **Cs[3]** in acetone-d_6

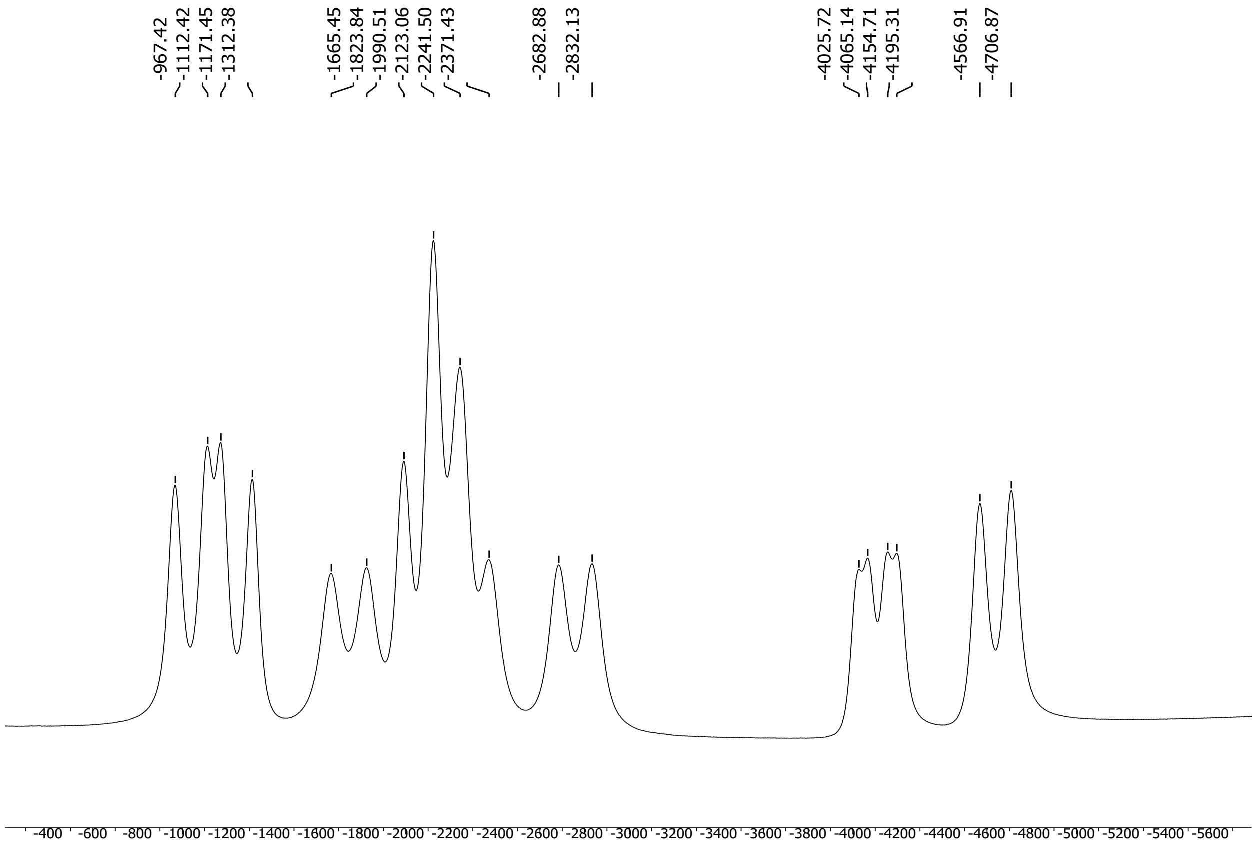

Figure S19. ^{11}B NMR spectrum of **Cs[3]** in acetone-d_6

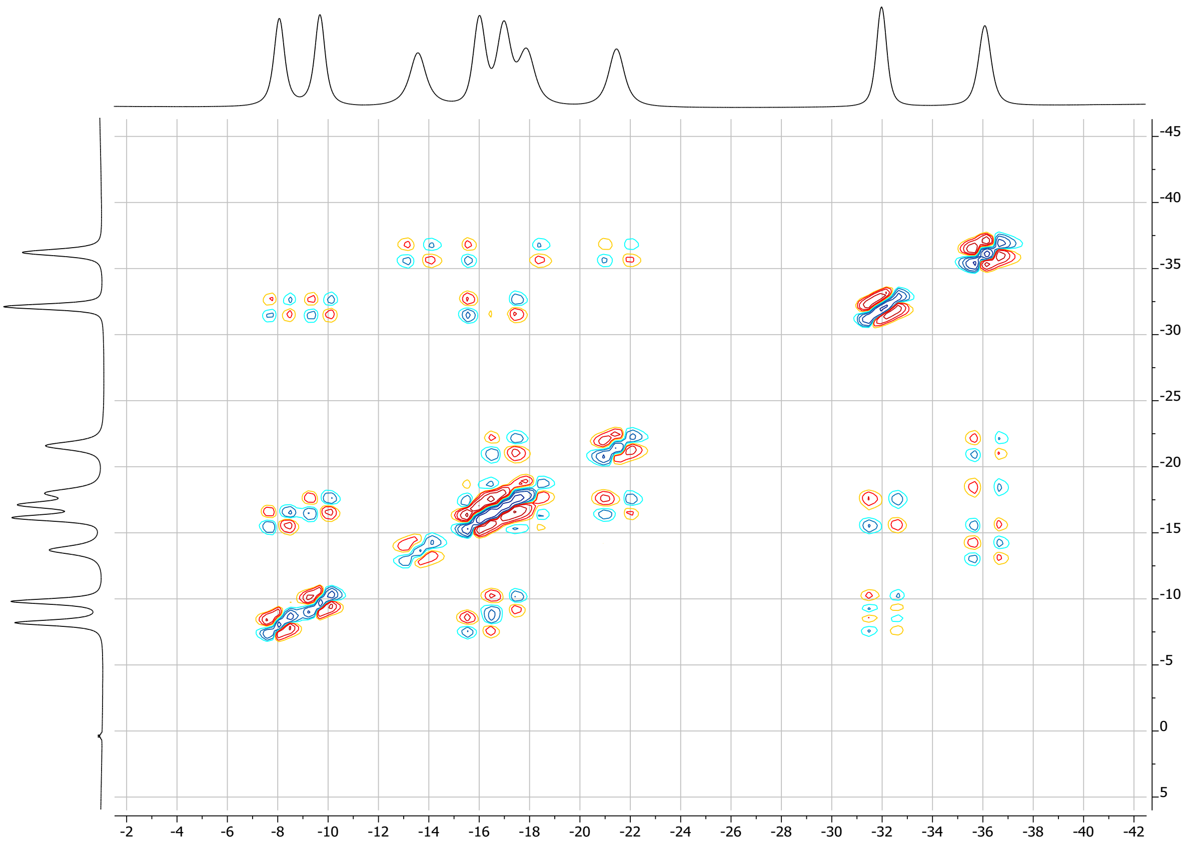

Figure S20. (BB) DQCOSY NMR spectrum of **Cs[3]** in acetone-d_6

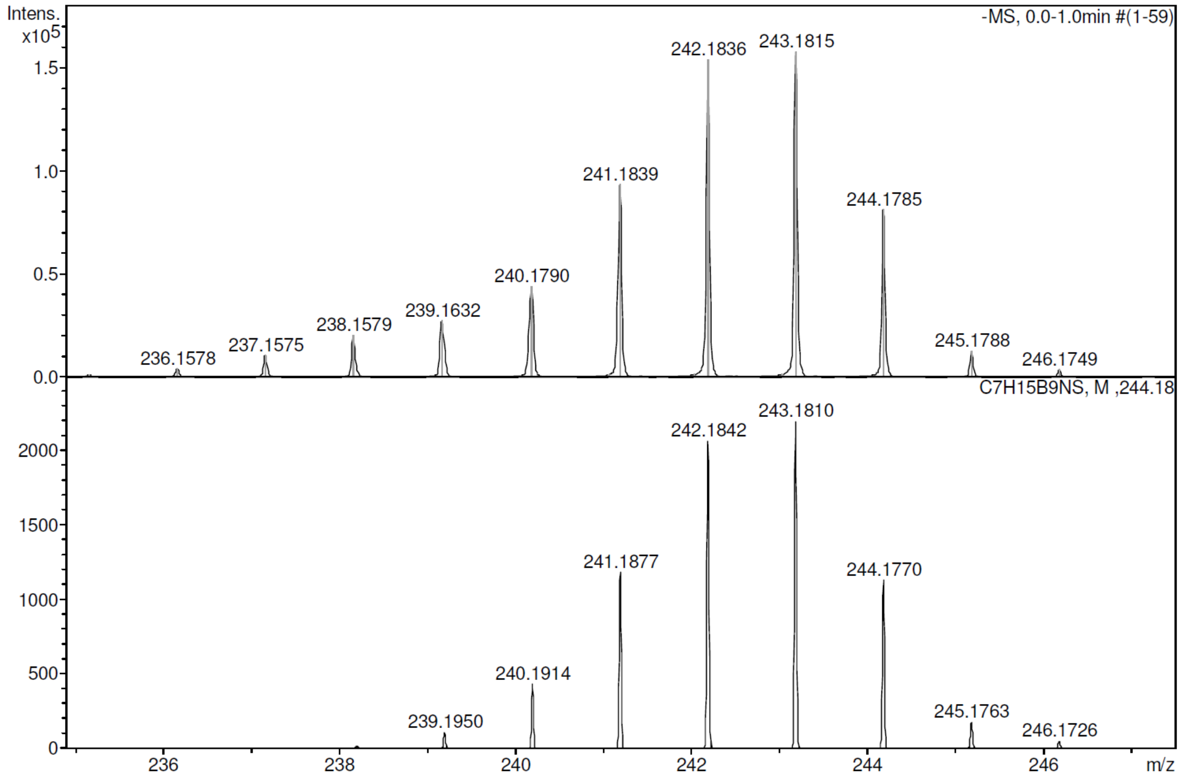

Figure S21. HRMS spectrum of **Cs[3]**

Spectral data for 1-(NC_5H_4-2'-CH_2S)-1,2-$C_2B_{10}H_{11}$ (**4**)

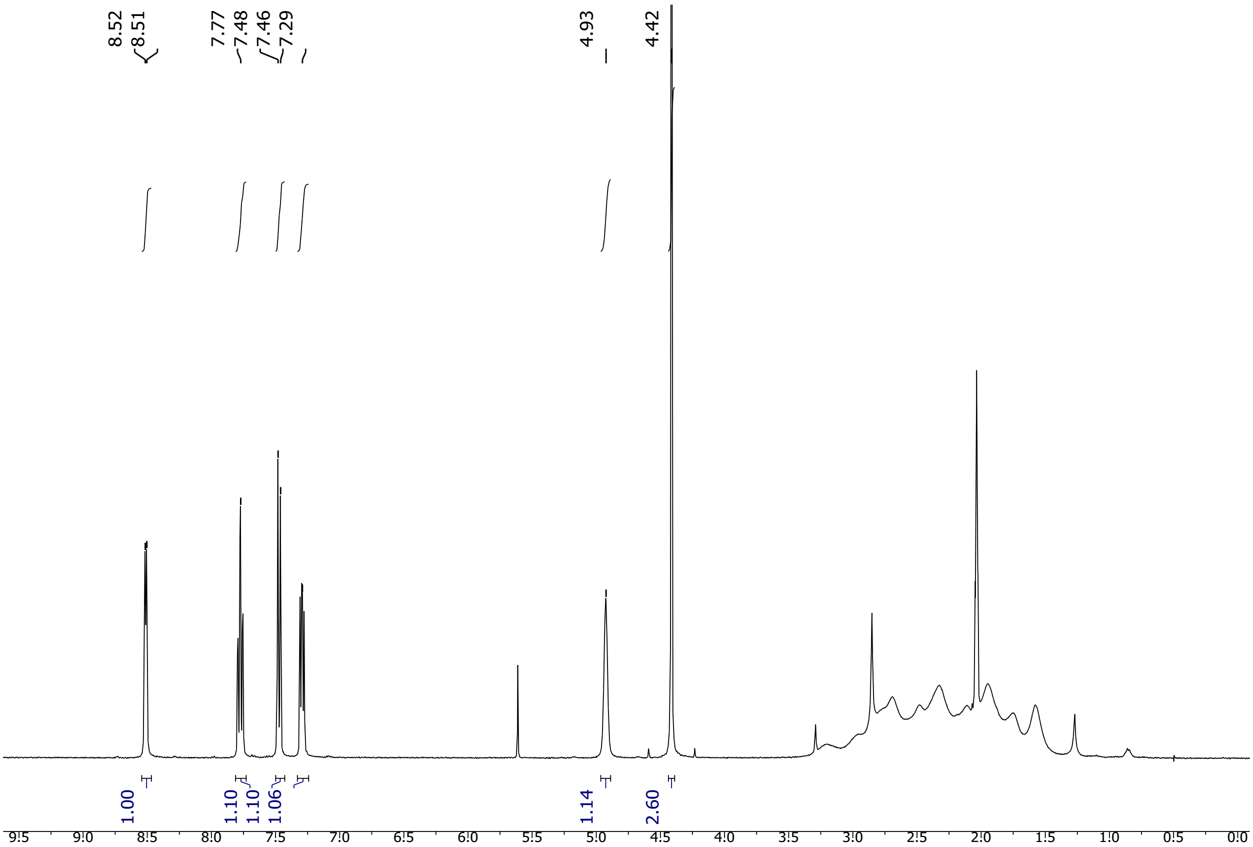

Figure S22. ^{1}H NMR spectrum of **4** in acetone-d_6

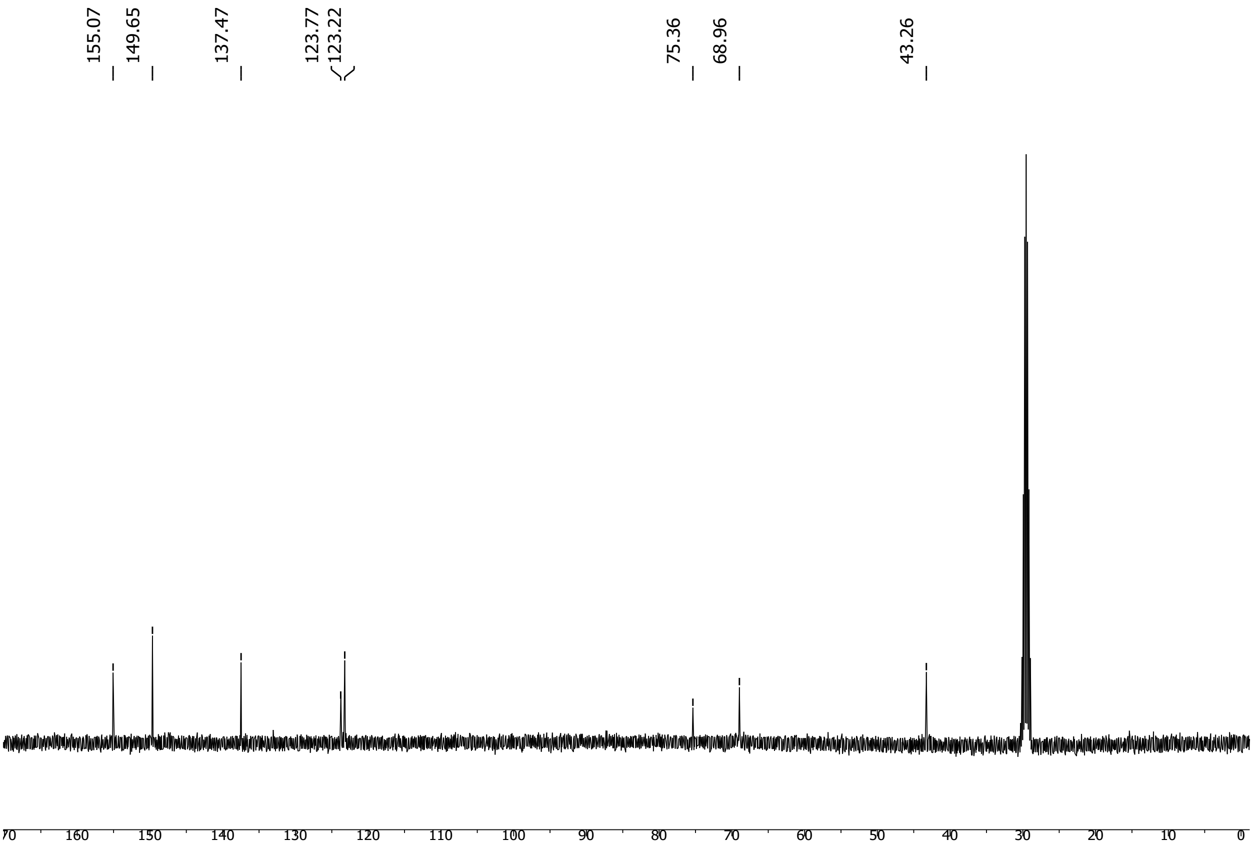

Figure S23. ^{13}C NMR spectrum of **4** in acetone-d_6

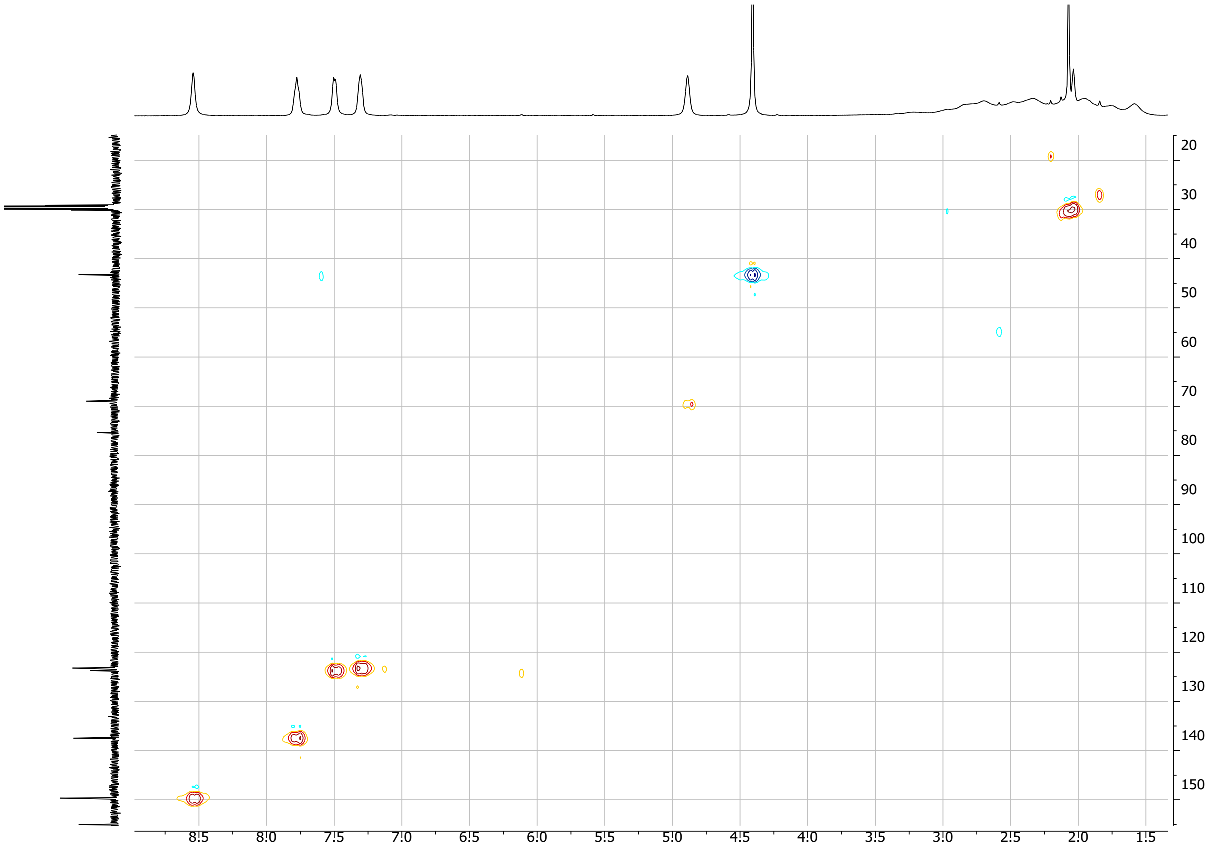

Figure S24. (HC) HSQC NMR spectrum of **4** in acetone-d_6

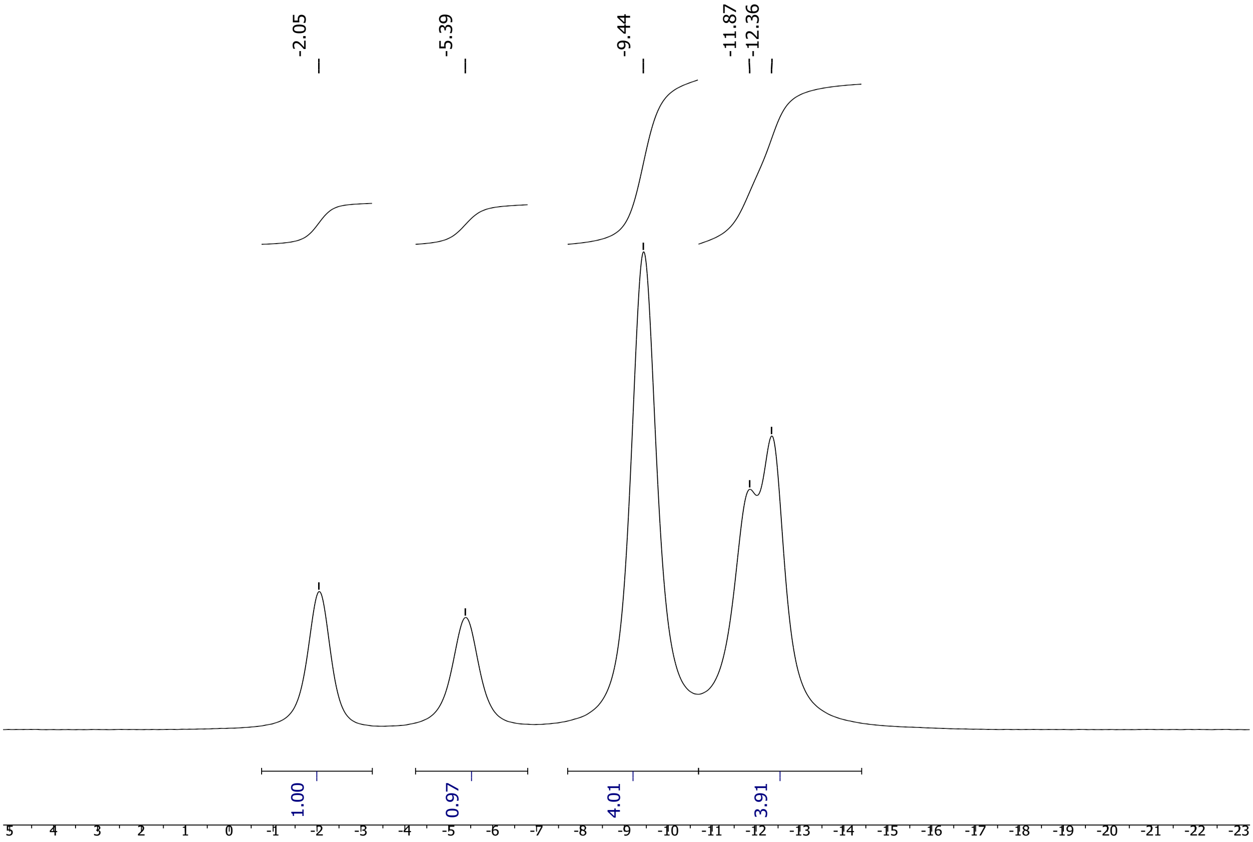

Figure S25. $^{11}B\{^1H\}$ NMR spectrum of **4** in acetone-d_6

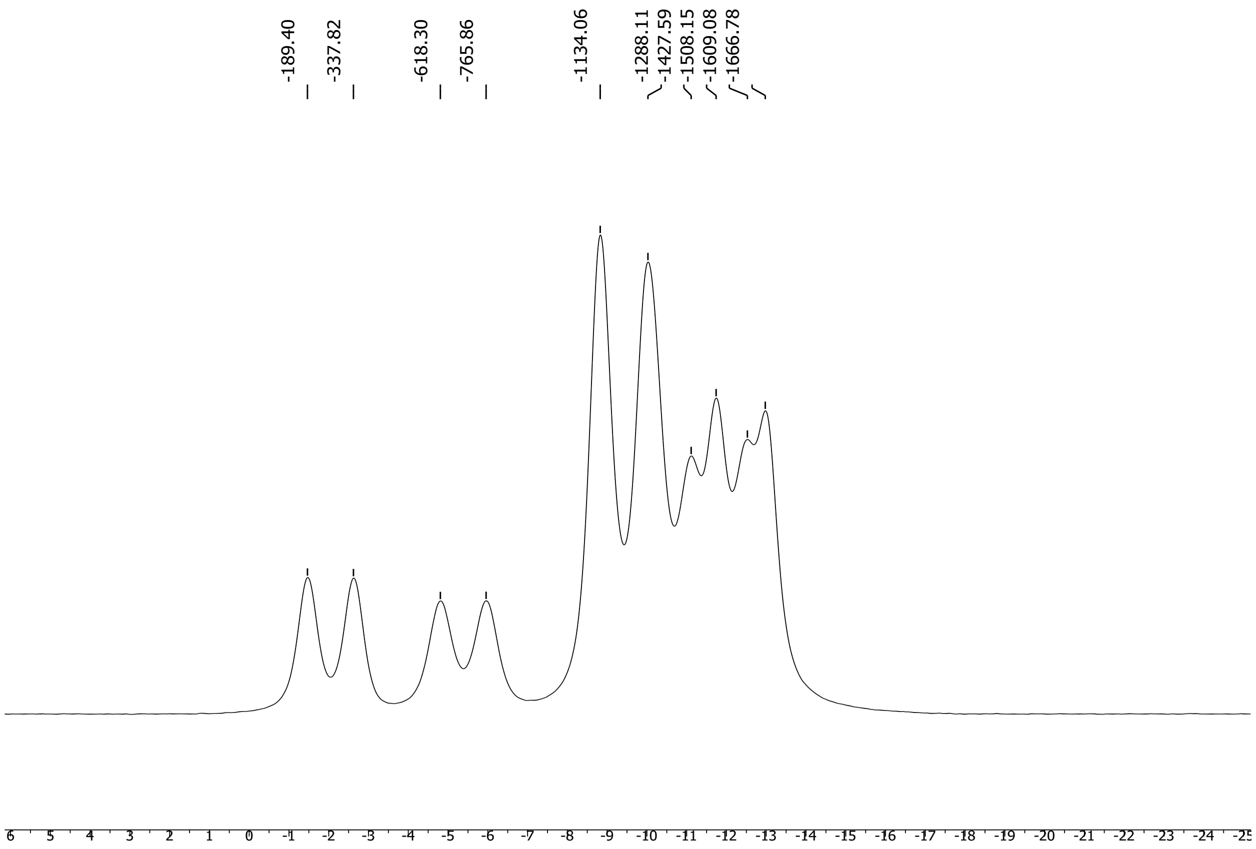

Figure S26. ^{11}B NMR spectrum of **4** in acetone-d_6

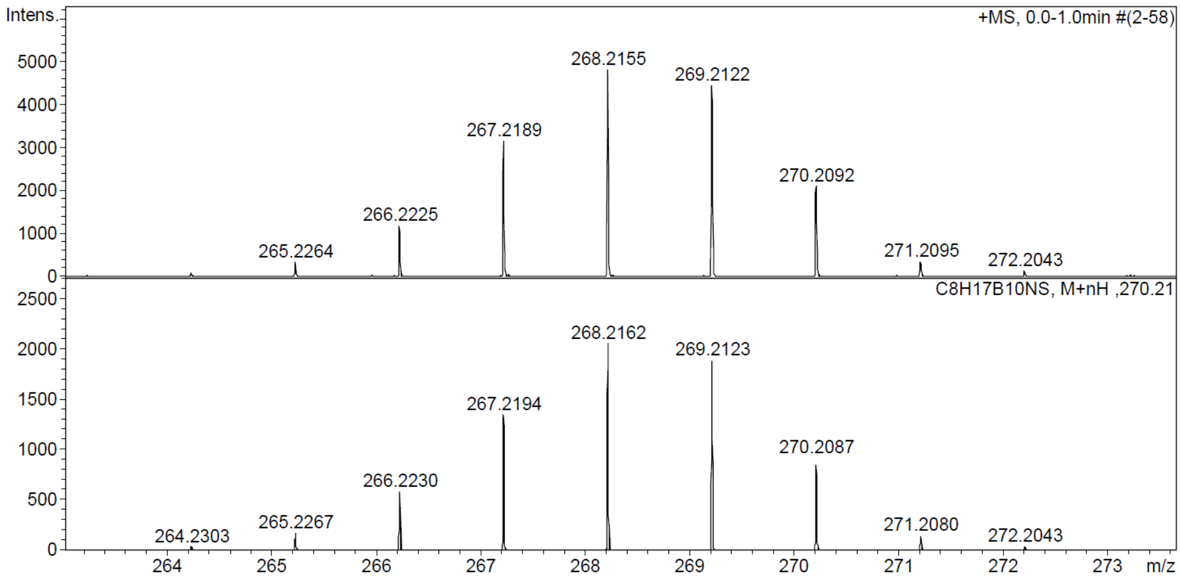

Figure S27. HRMS spectrum of **4**

Spectral data for Cs[7-(NC_5H_4-2'-CH_2S)-7,8-$C_2B_9H_{11}$] (**5**)

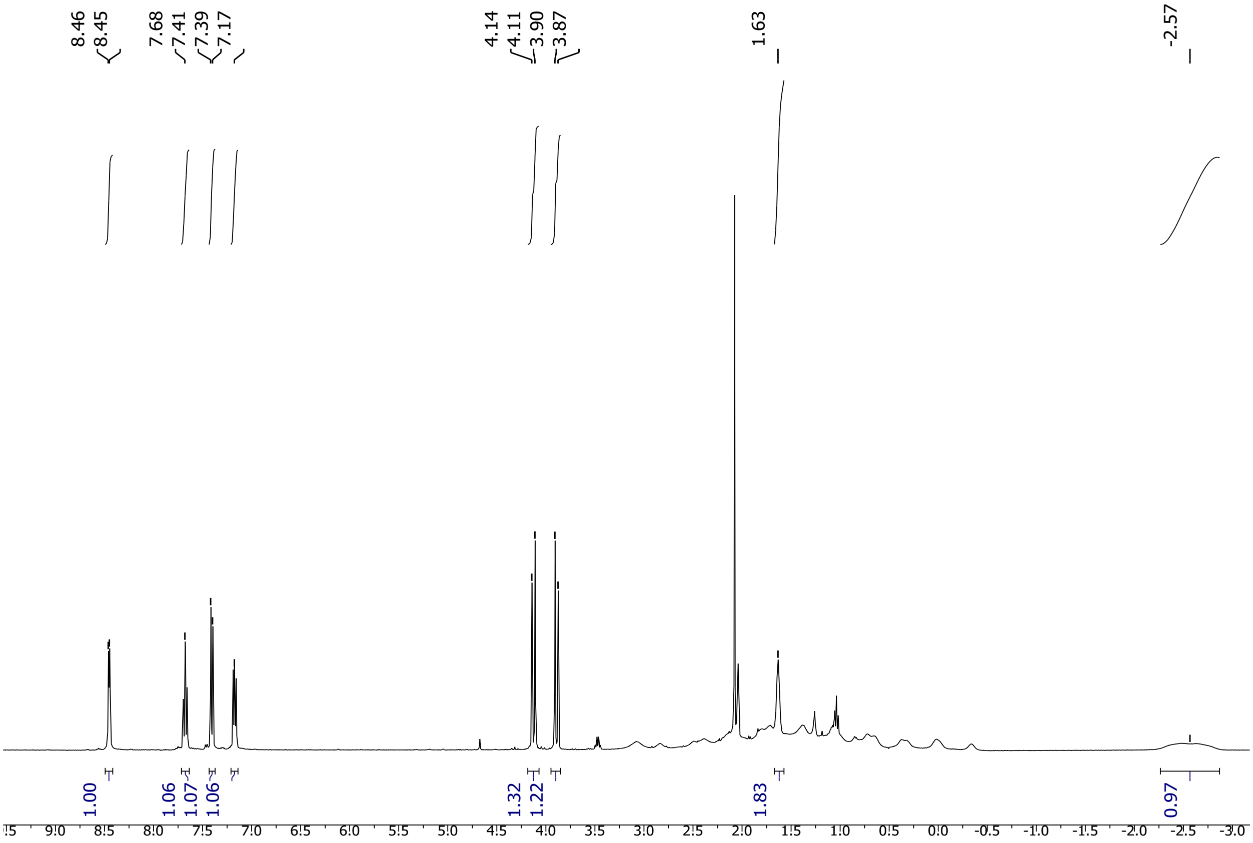

Figure S28. ^{1}H NMR spectrum of **5** in acetone-d_6

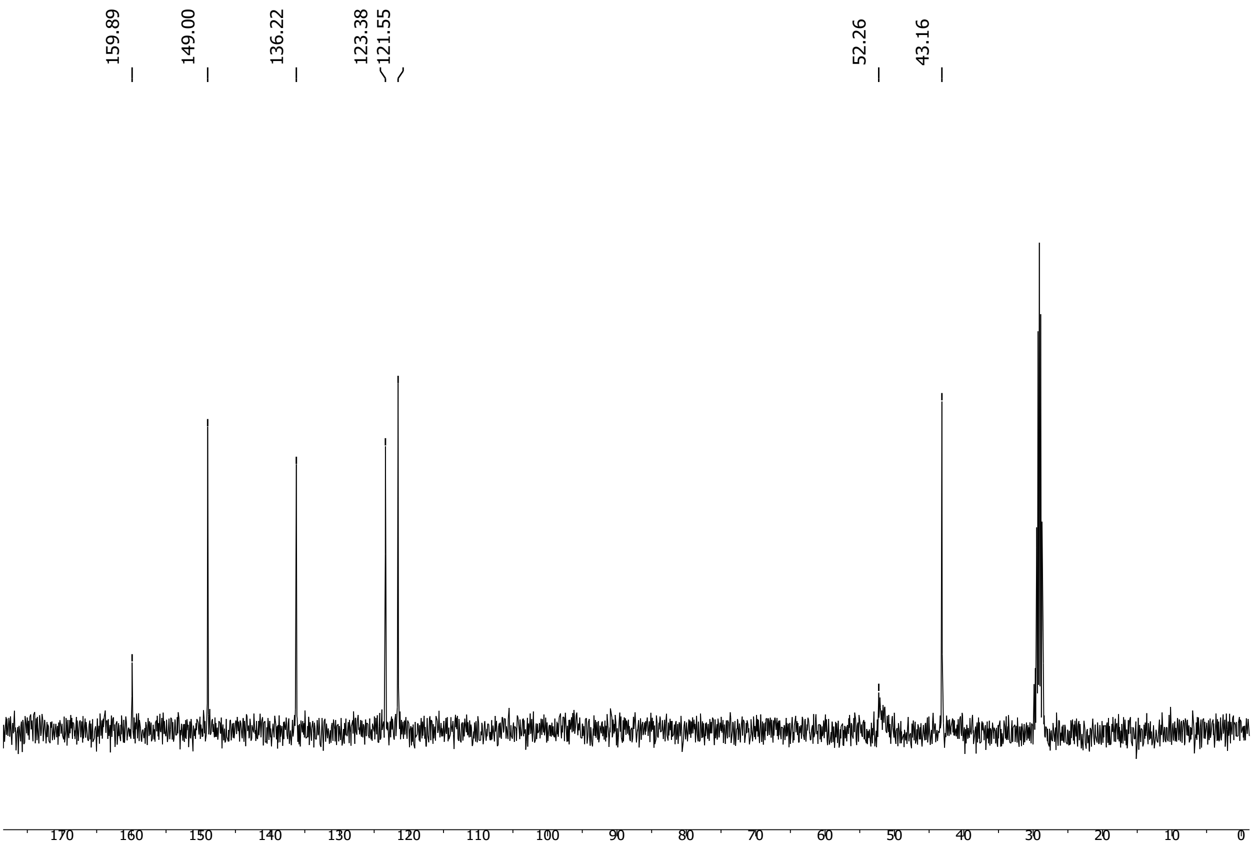

Figure S29. ^{13}C NMR spectrum of **5** in acetone-d_6

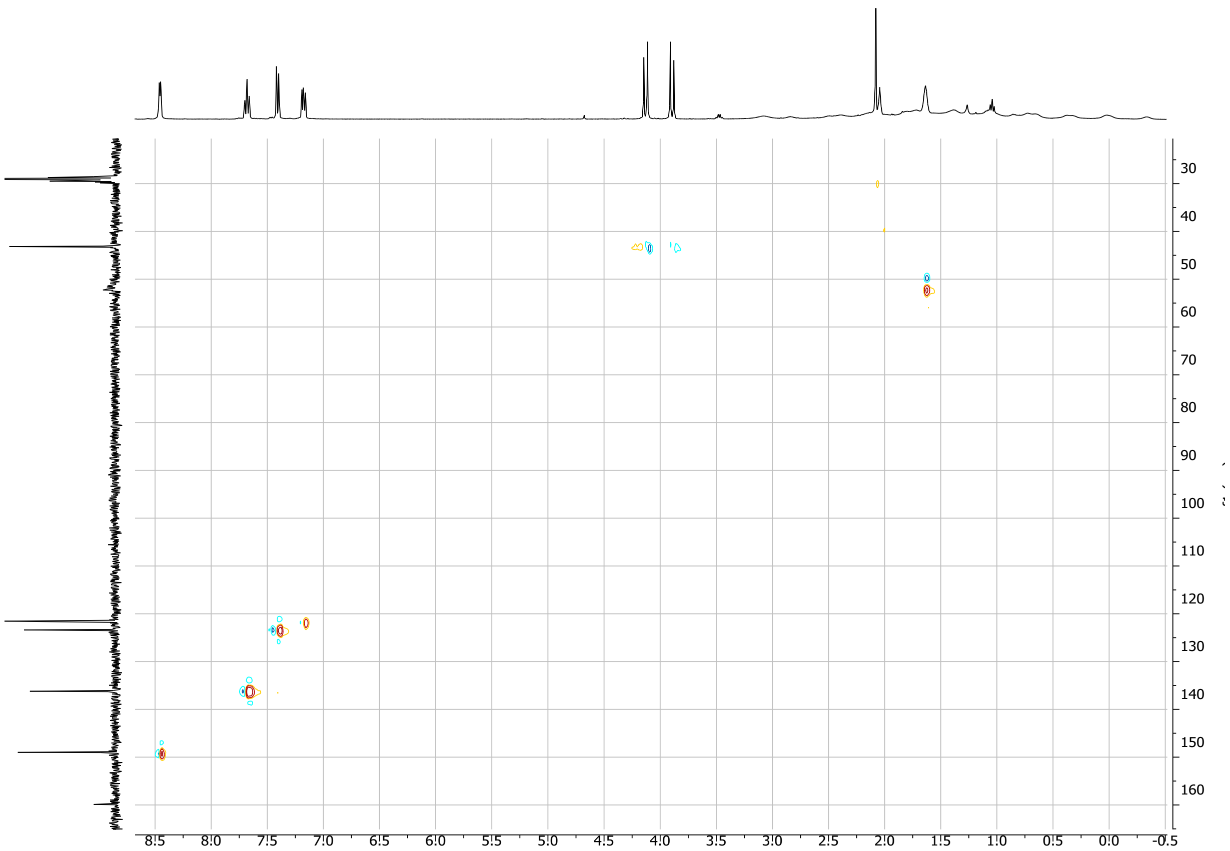

Figure S30. (HC) HSQC NMR spectrum of **5** in acetone-d_6

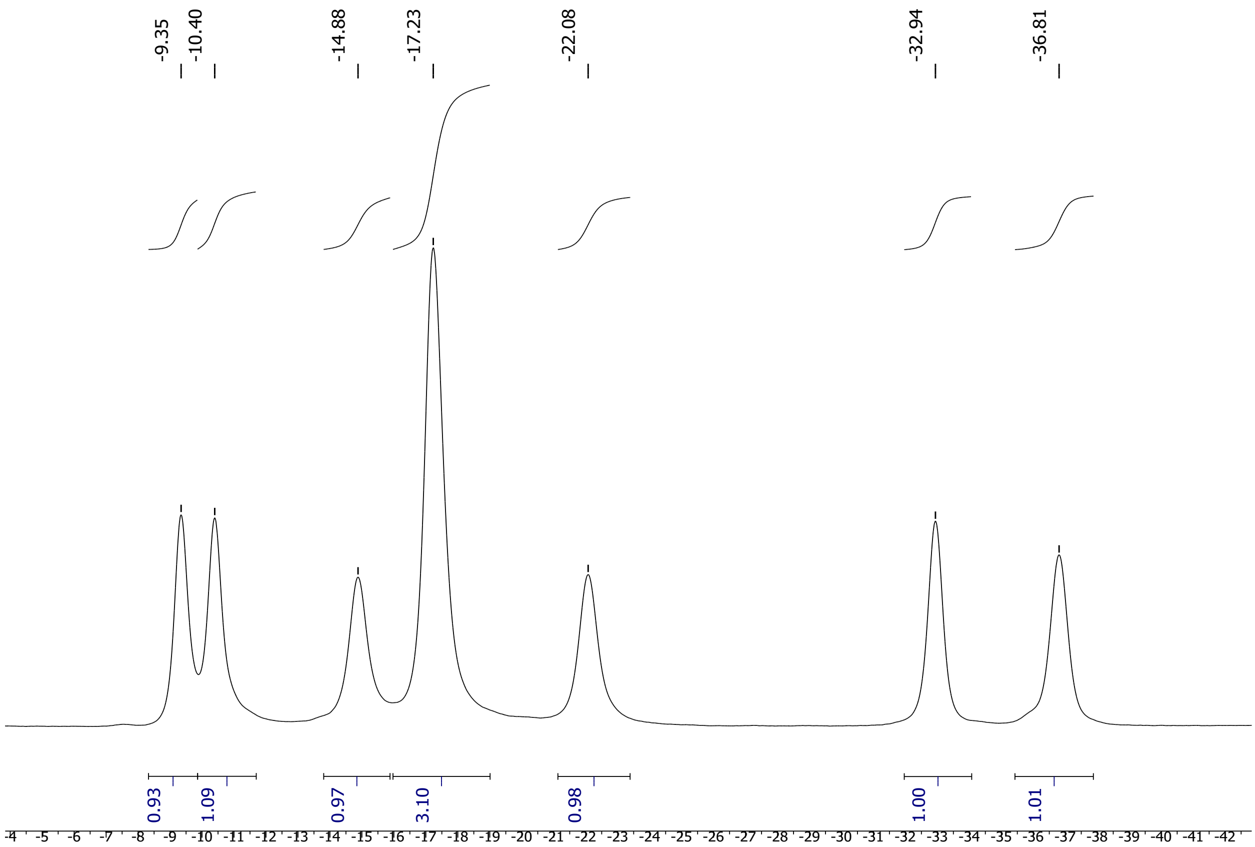

Figure S31. $^{11}B\{^{1}H\}$ NMR spectrum of **5** in acetone-d_6

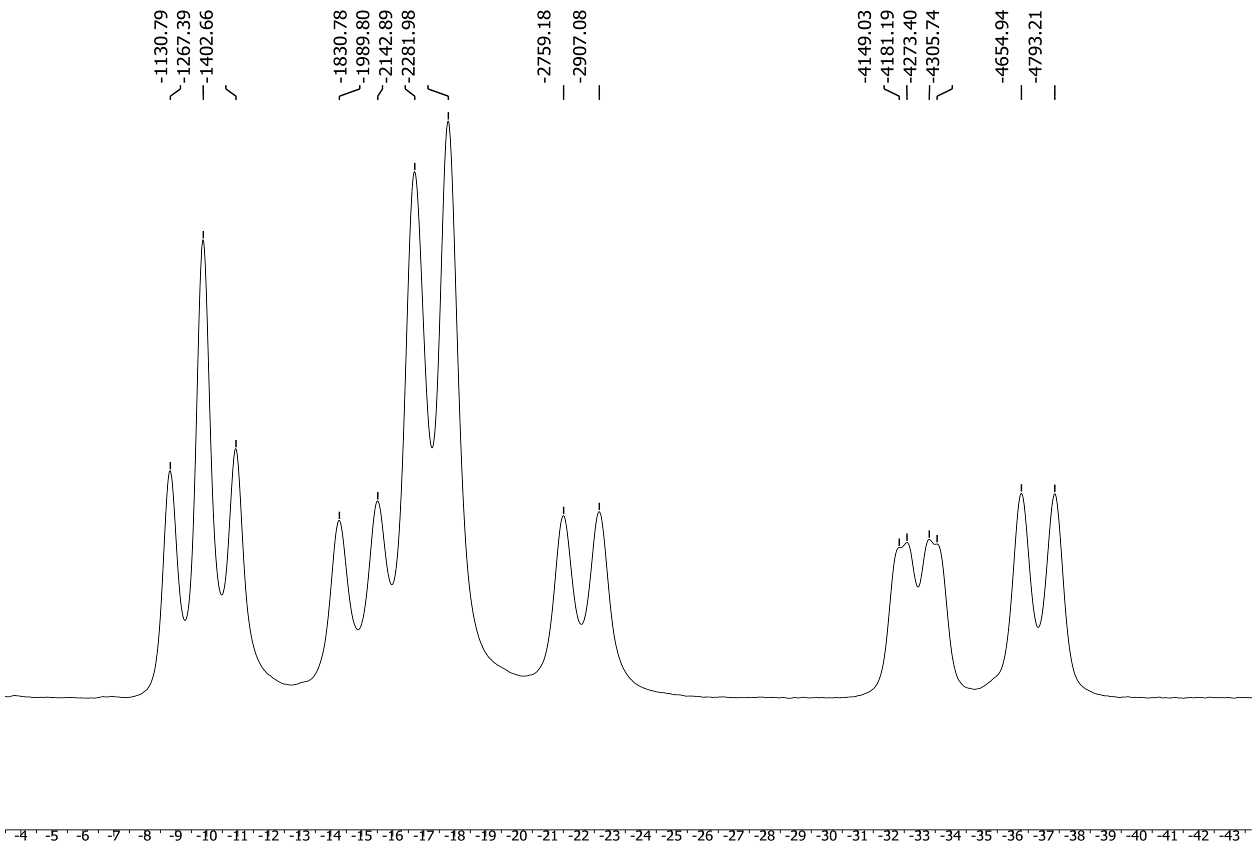

Figure S32. ^{11}B NMR spectrum of **5** in acetone-d_6

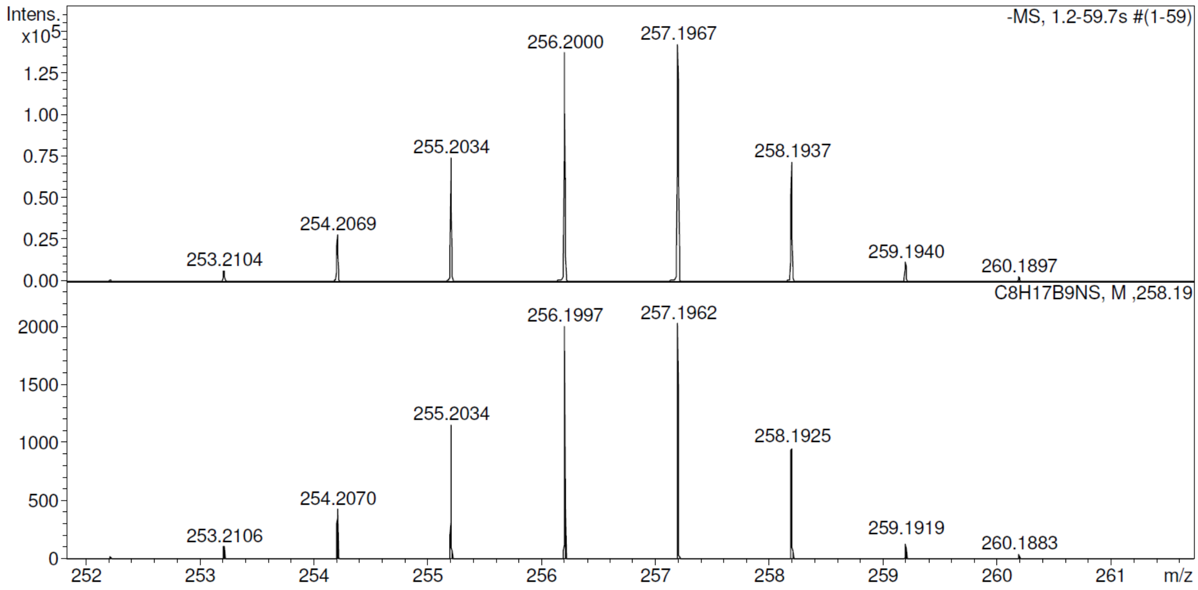

Figure S33. HRMS spectrum of **5**

Spectral data for 3-Ph_3P-3-(4(7)-NC_5H_4-2'-S)-*closo*-3,1,2-$NiC_2B_9H_{10}$ (**6**)

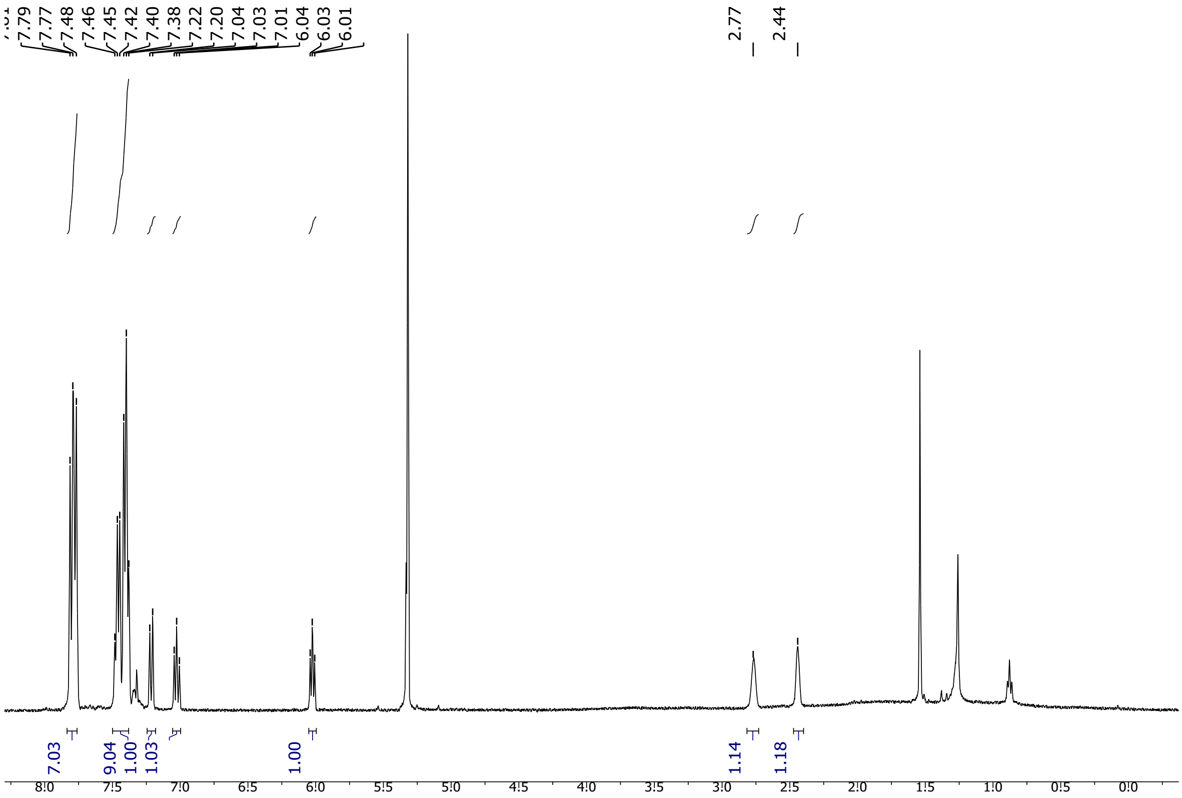

Figure S34. ^{1}H NMR spectrum of **6** in CD_2Cl_2

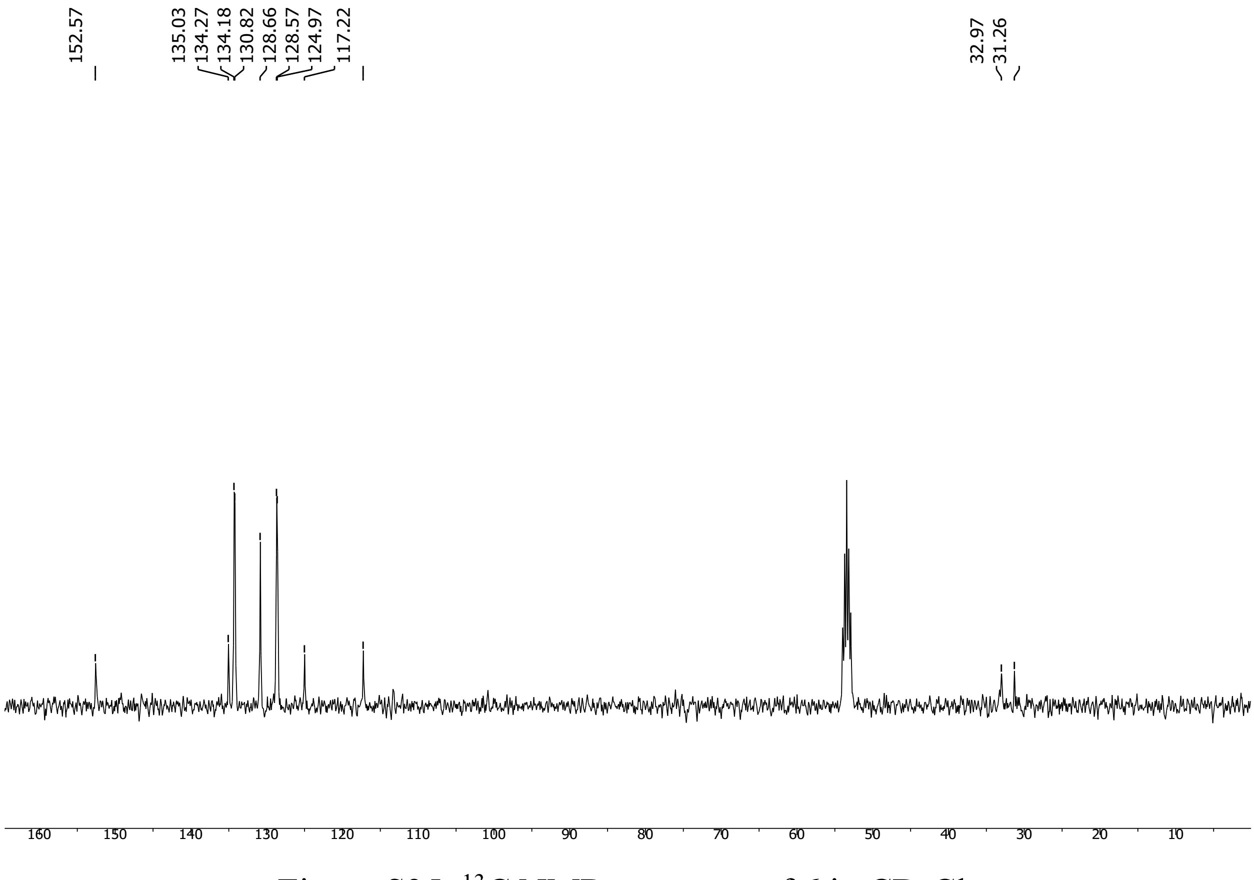

Figure S35. ^{13}C NMR spectrum of **6** in CD_2Cl_2

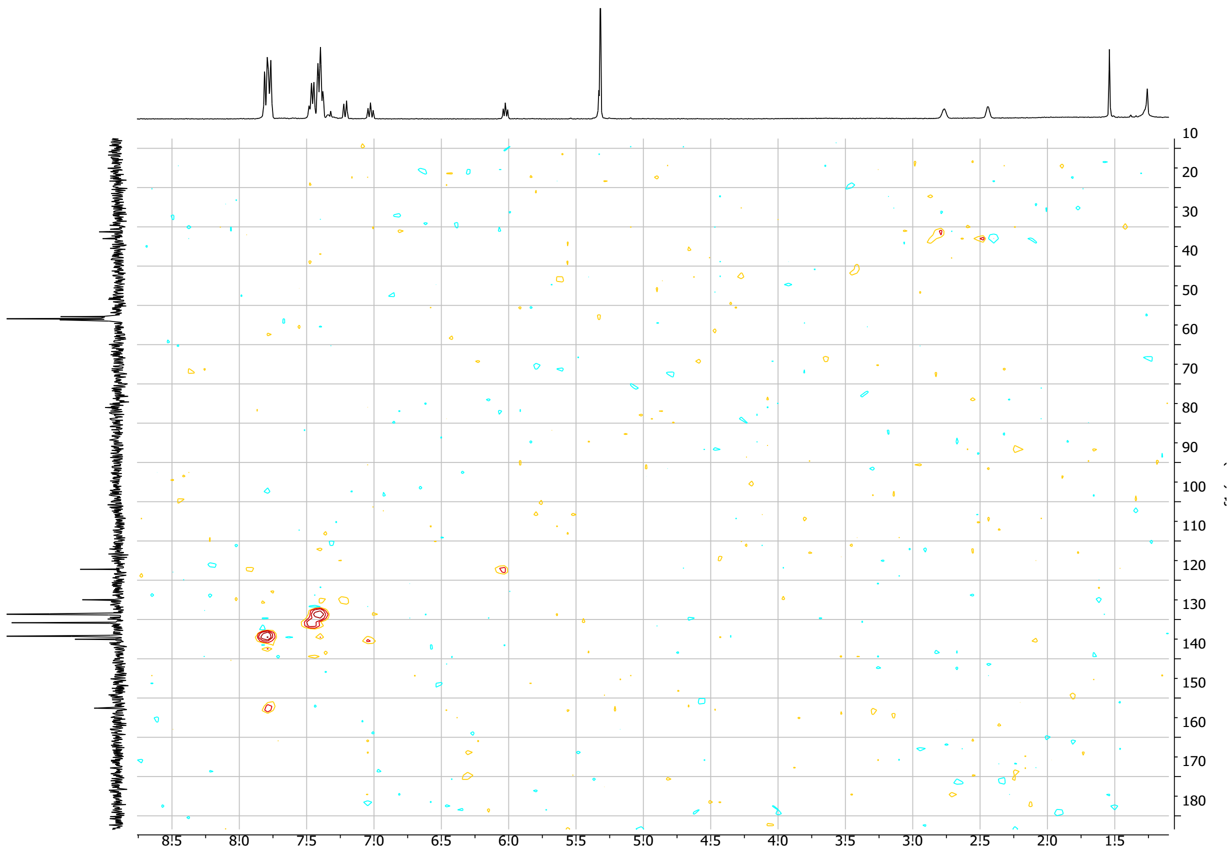

Figure S36. (HC) HSQC NMR spectrum of **6** in CD_2Cl_2

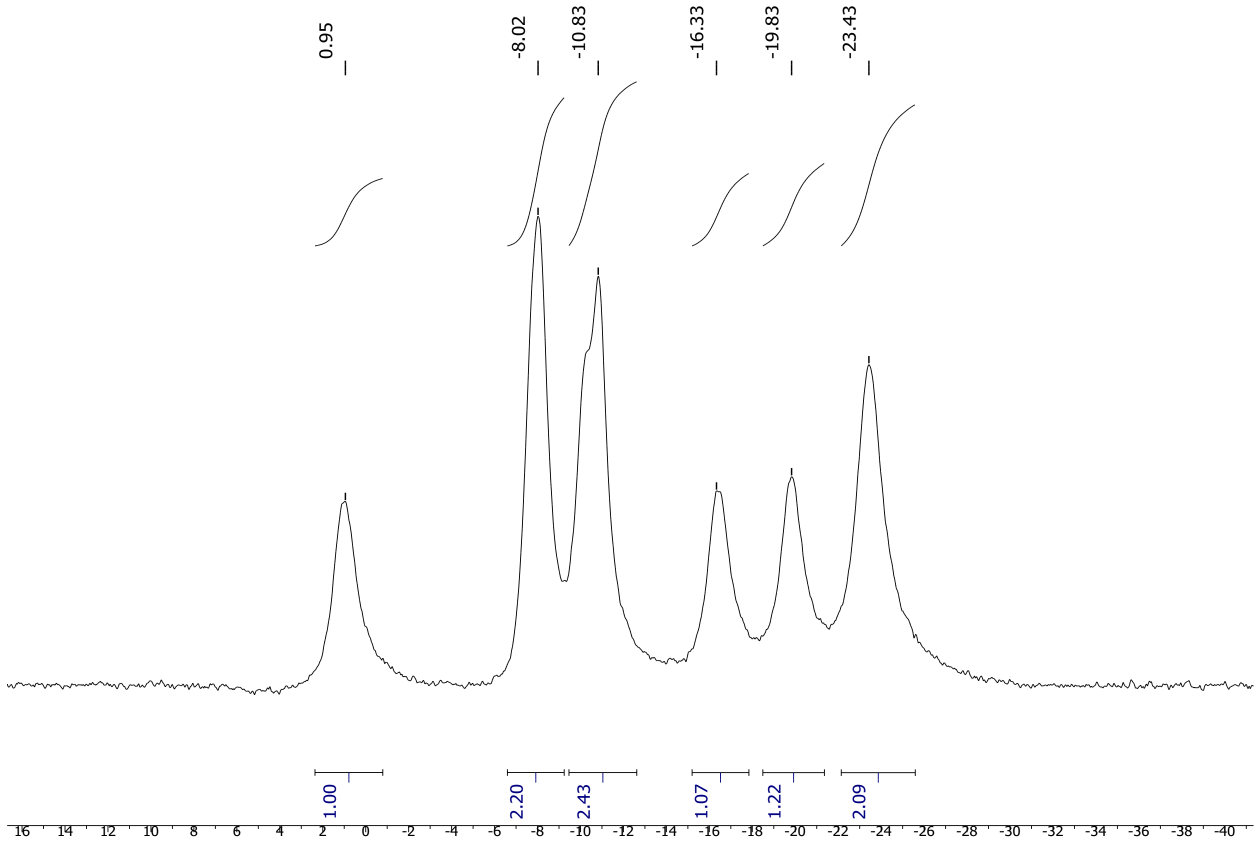

Figure S37. $^{11}B\{^1H\}$ NMR spectrum of **6** in CD_2Cl_2

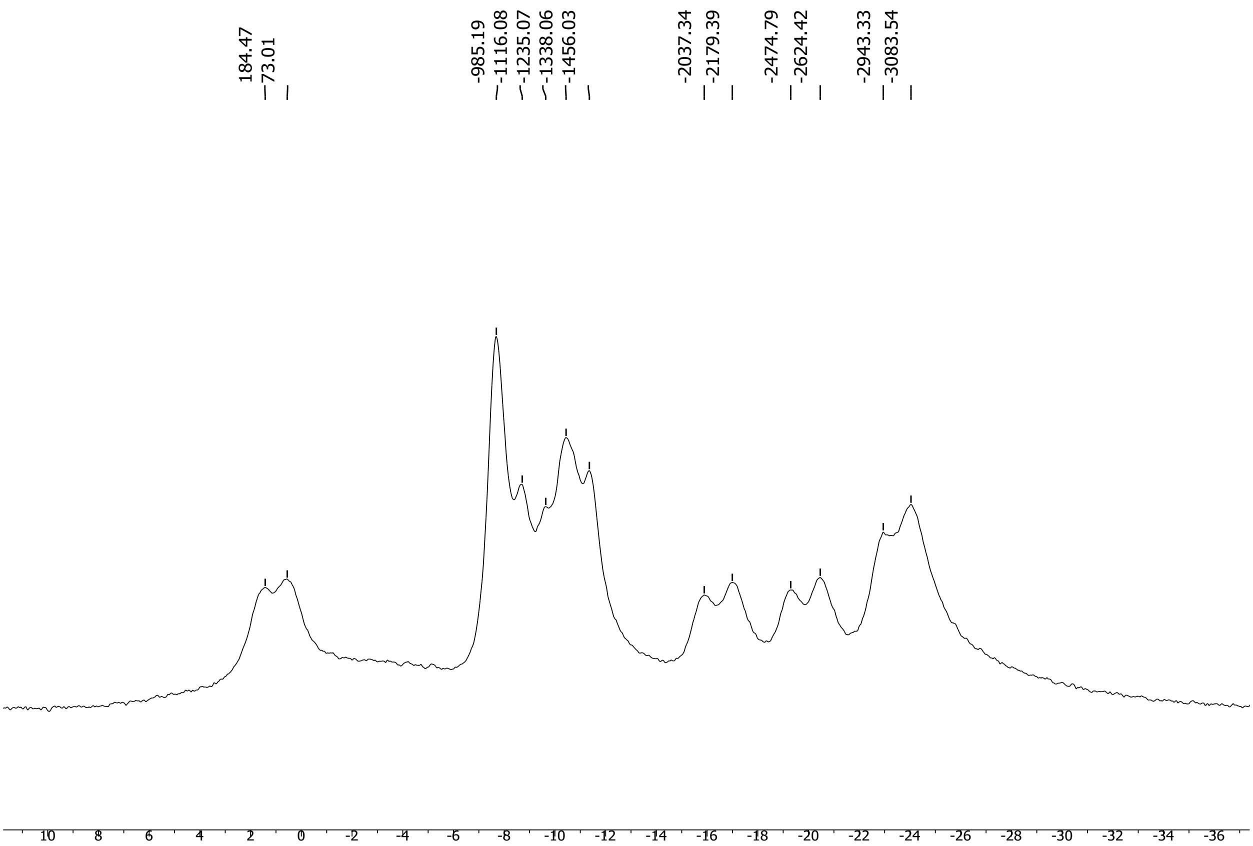

Figure S38. ^{11}B NMR spectrum of **6** in CD_2Cl_2

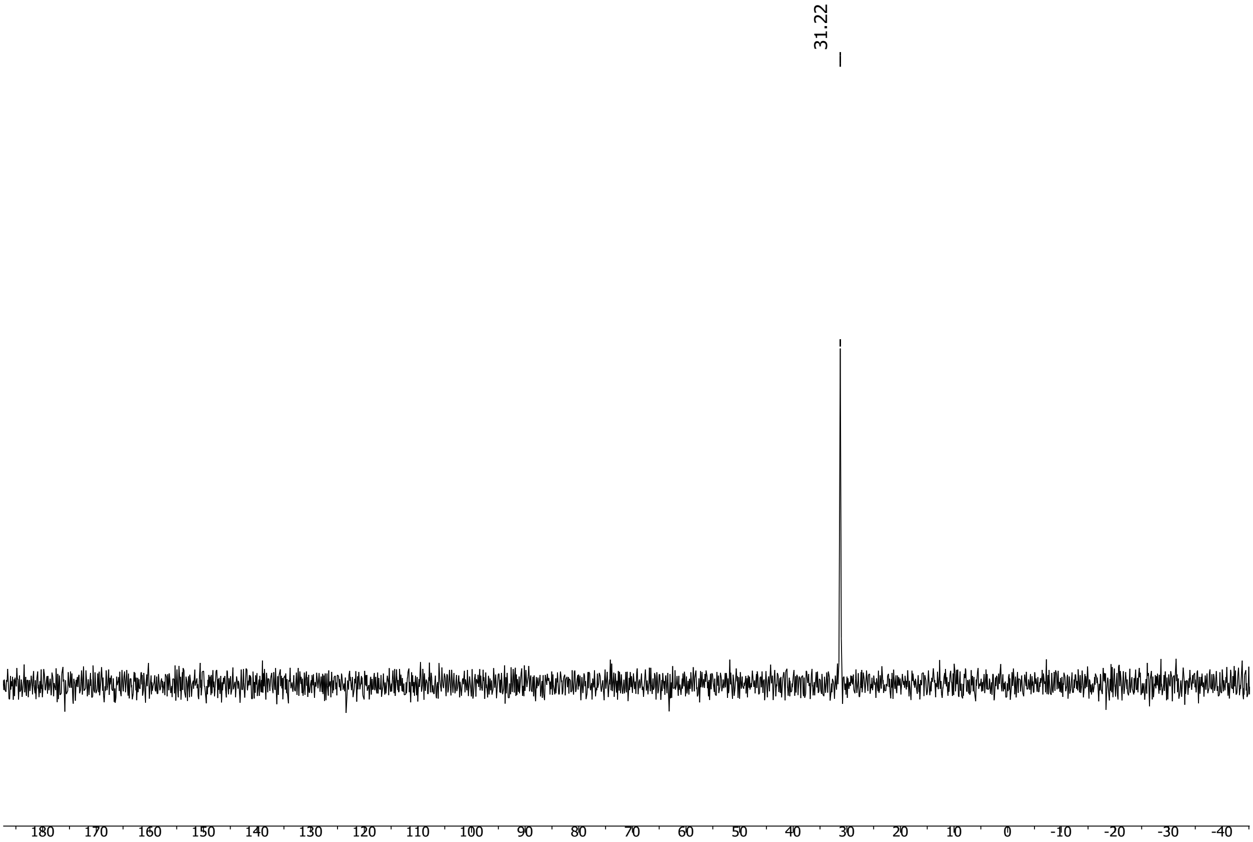

Figure S39. ^{31}P NMR spectrum of **6** in CD_2Cl_2

Spectral data for 3-Ph_3P-3-(4(7)-NC_5H_4-2'-S)-*closo*-3,1,2-$PdC_2B_9H_{10}$ (**7**)

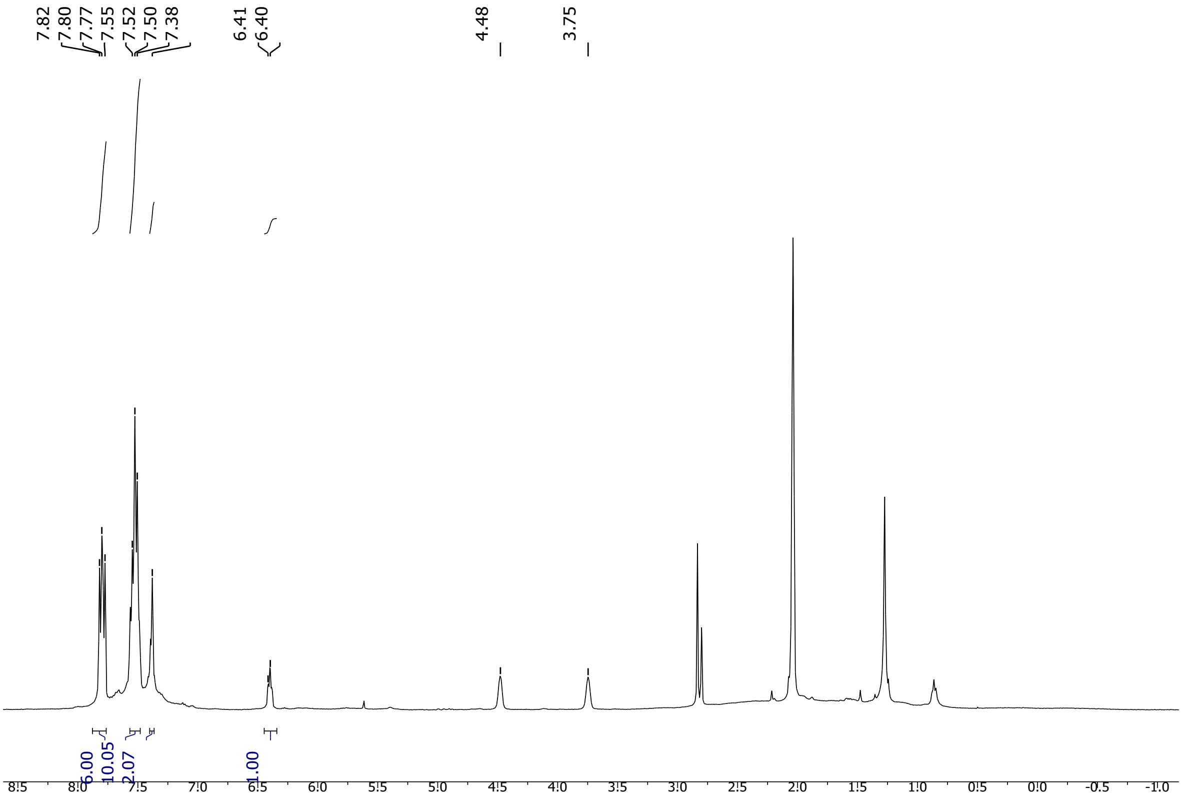

Figure S40. ^1H NMR spectrum of **7** in acetone-d_6

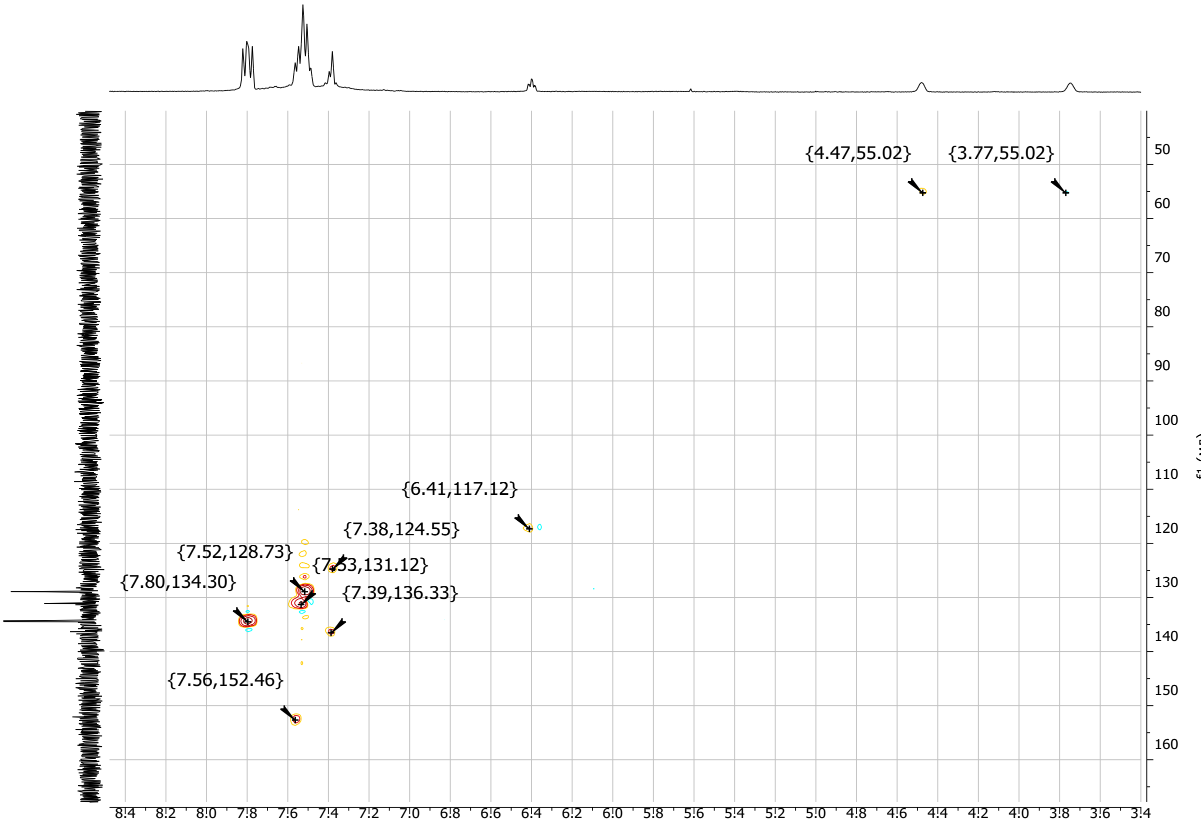

Figure S41. (HC) HSQC NMR spectrum of **7** in acetone-d_6

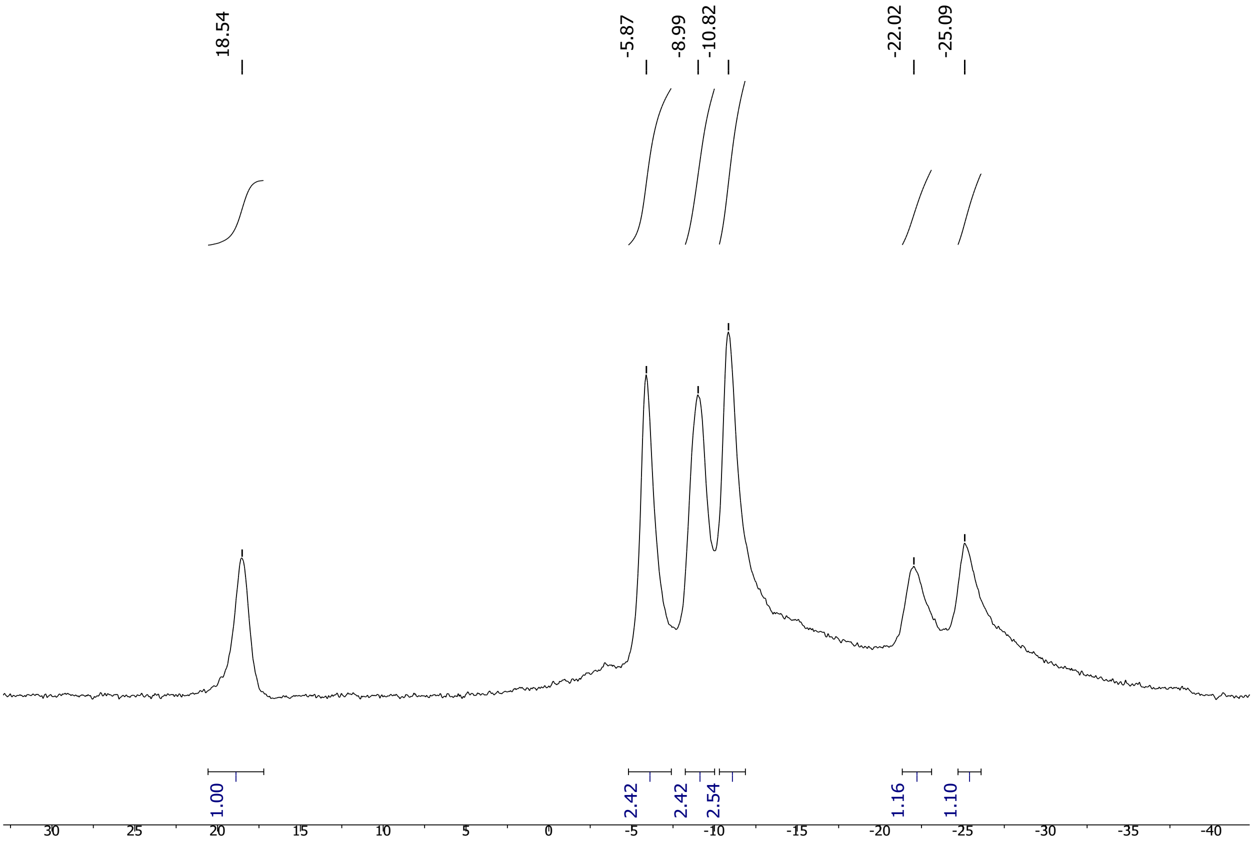

Figure S42. $^{11}B\{^{1}H\}$ NMR spectrum of **7** in in acetone-d_6

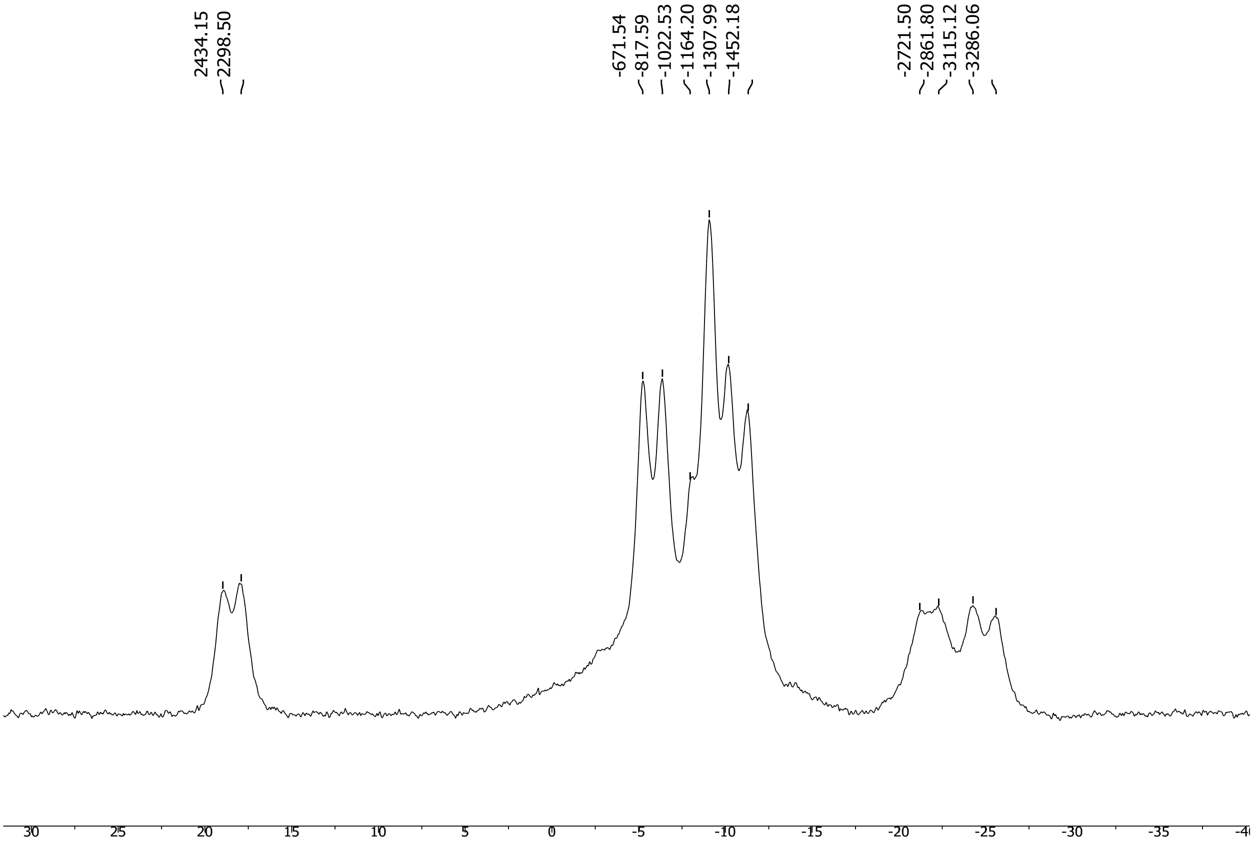

Figure S43. ^{11}B NMR spectrum of **7** in in acetone-d_6

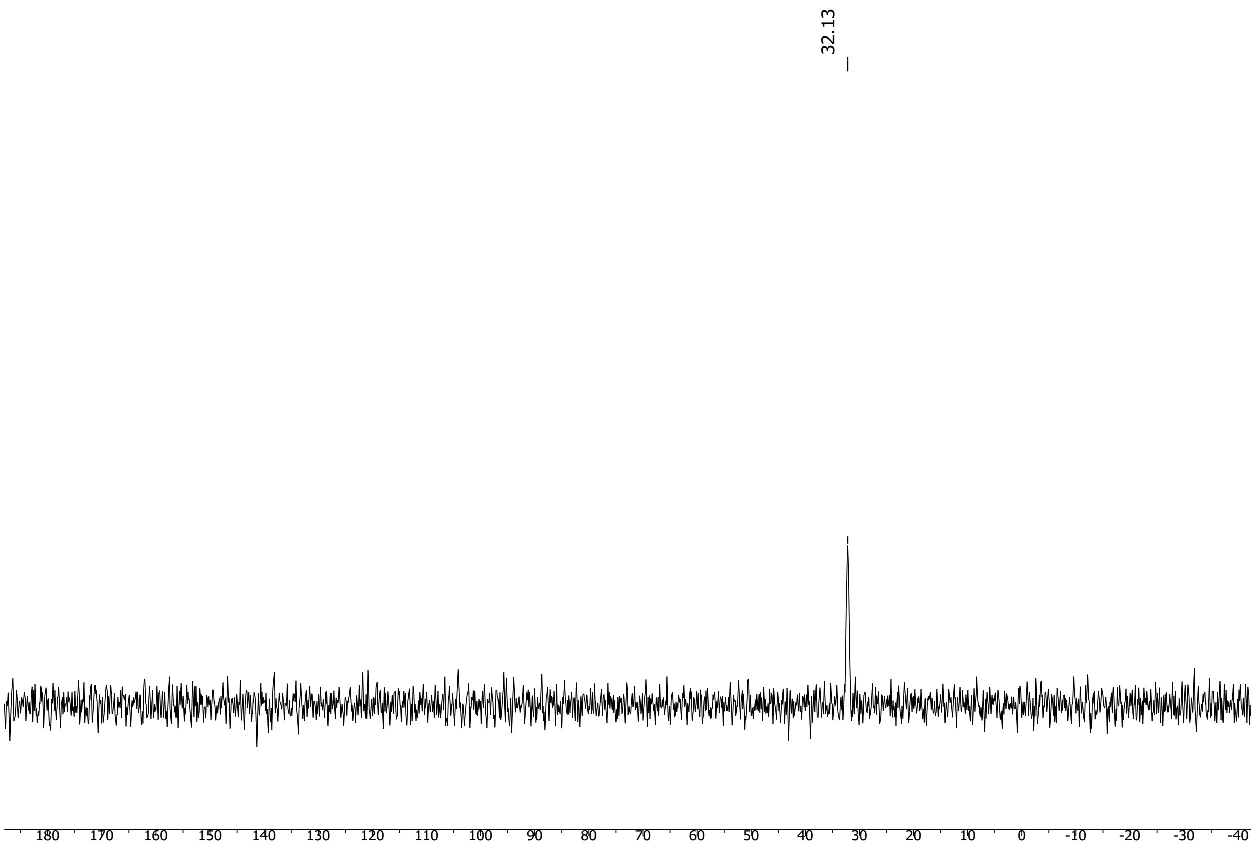

Figure S44. ^{31}P NMR spectrum of **7** in in acetone-d_6

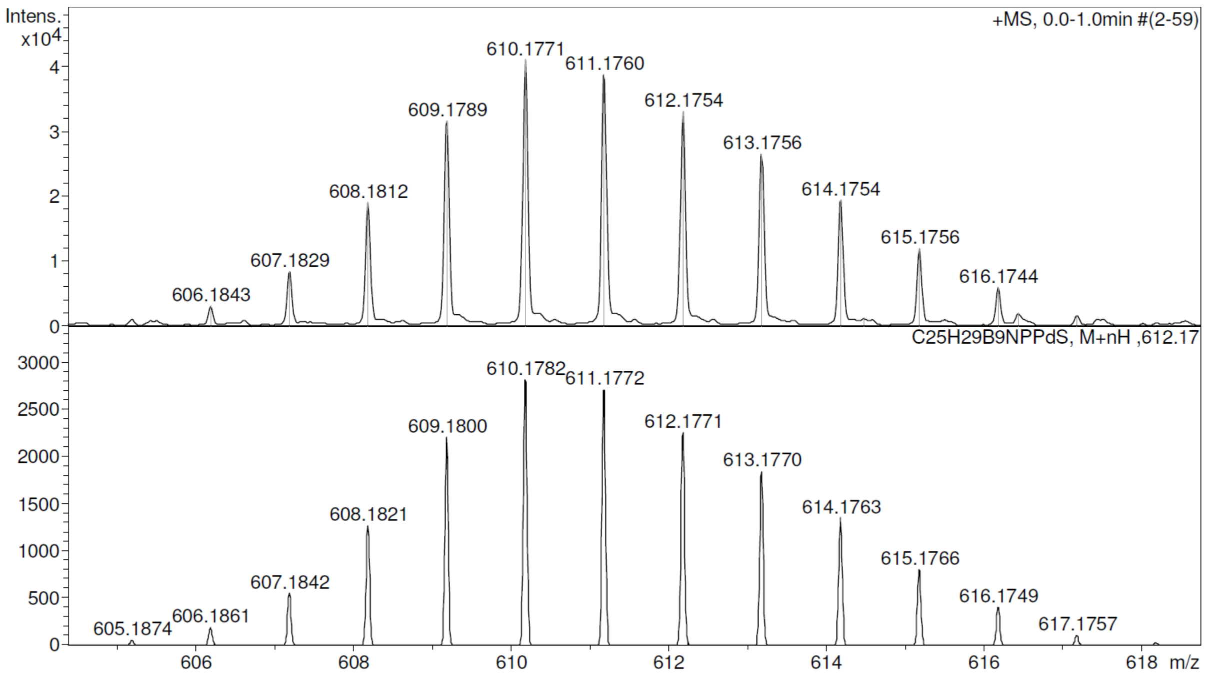

Figure S45. HRMS spectrum of **7**

Spectral data for 3-Ph_3P-3-(1(2)-NC_5H_4-2'-S)-*closo*-3,1,2-$NiC_2B_9H_{10}$ (**8**)

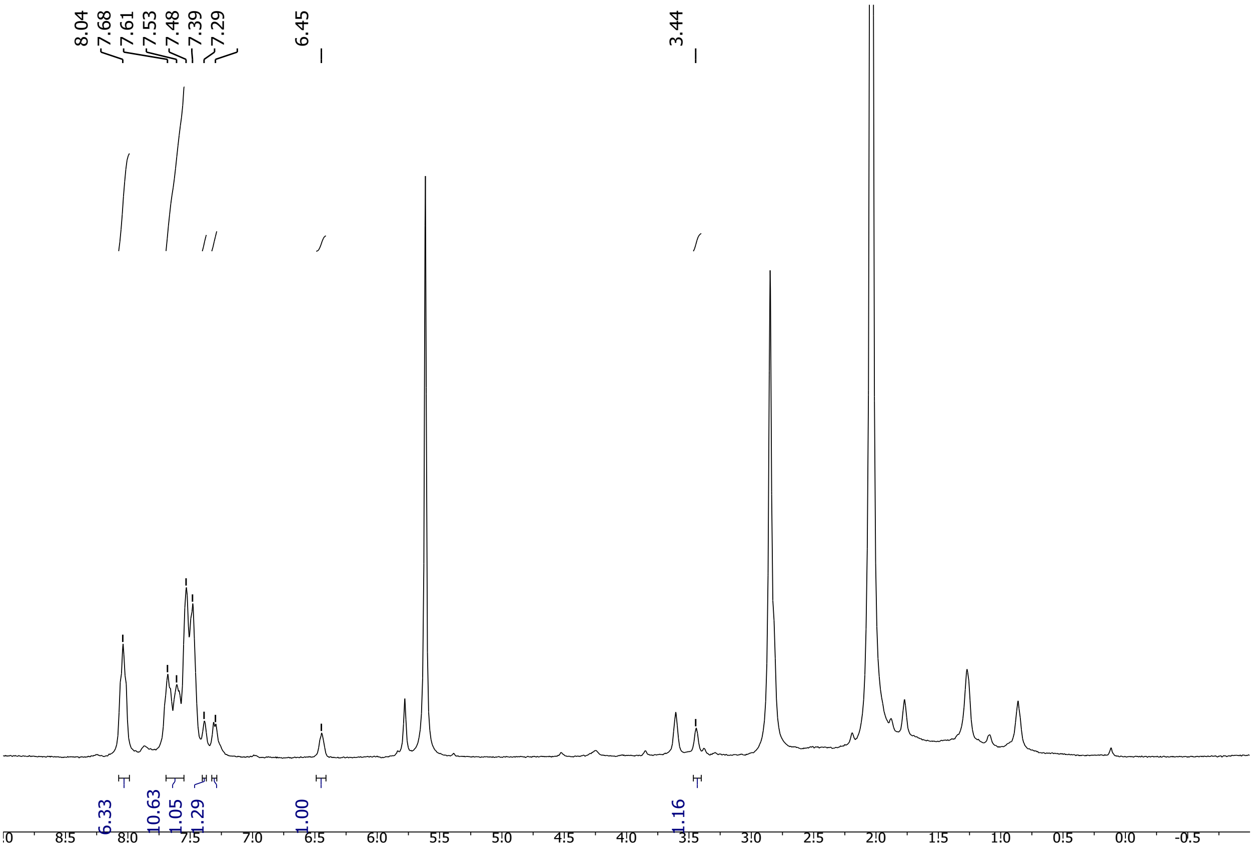

Figure S46. ^{1}H NMR spectrum of **8** in acetone-d_6

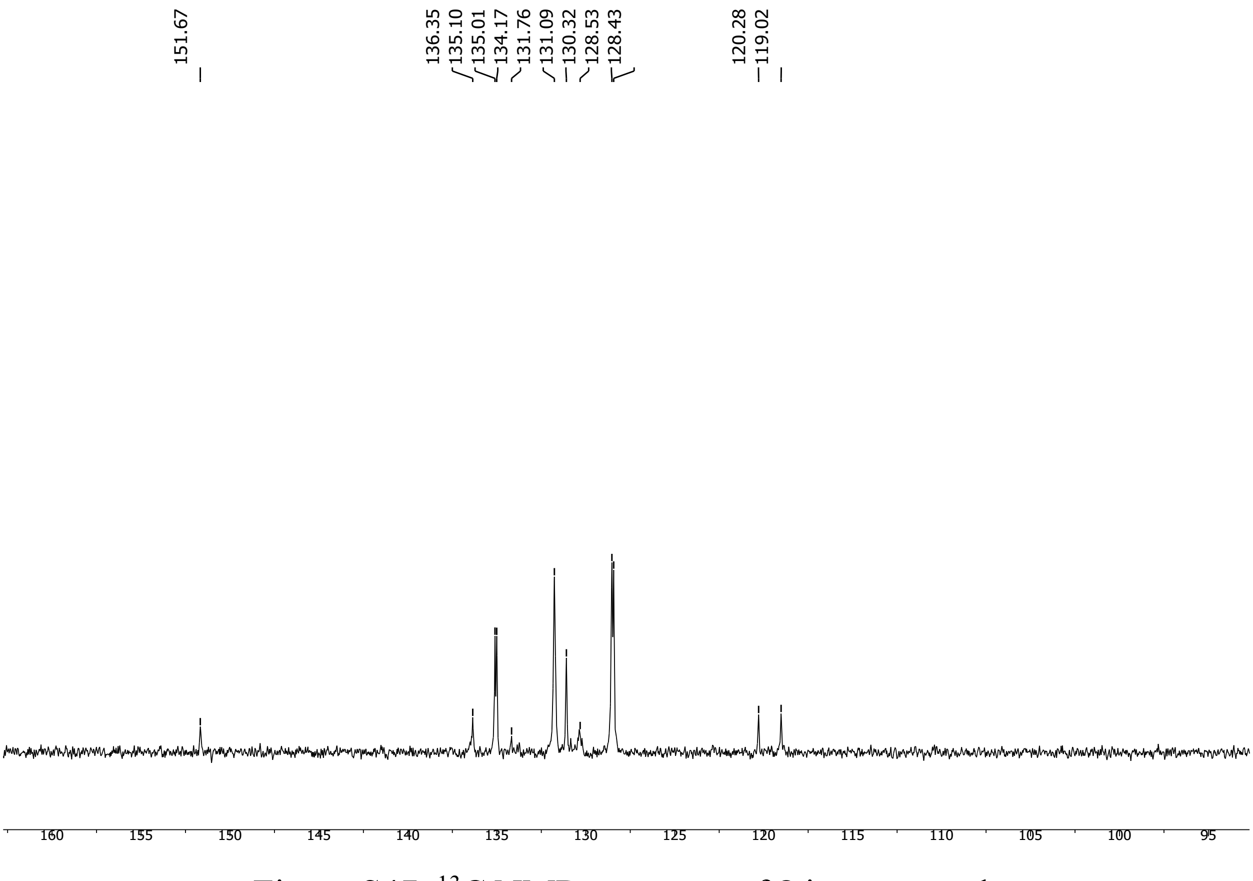

Figure S47. ^{13}C NMR spectrum of **8** in acetone-d_6

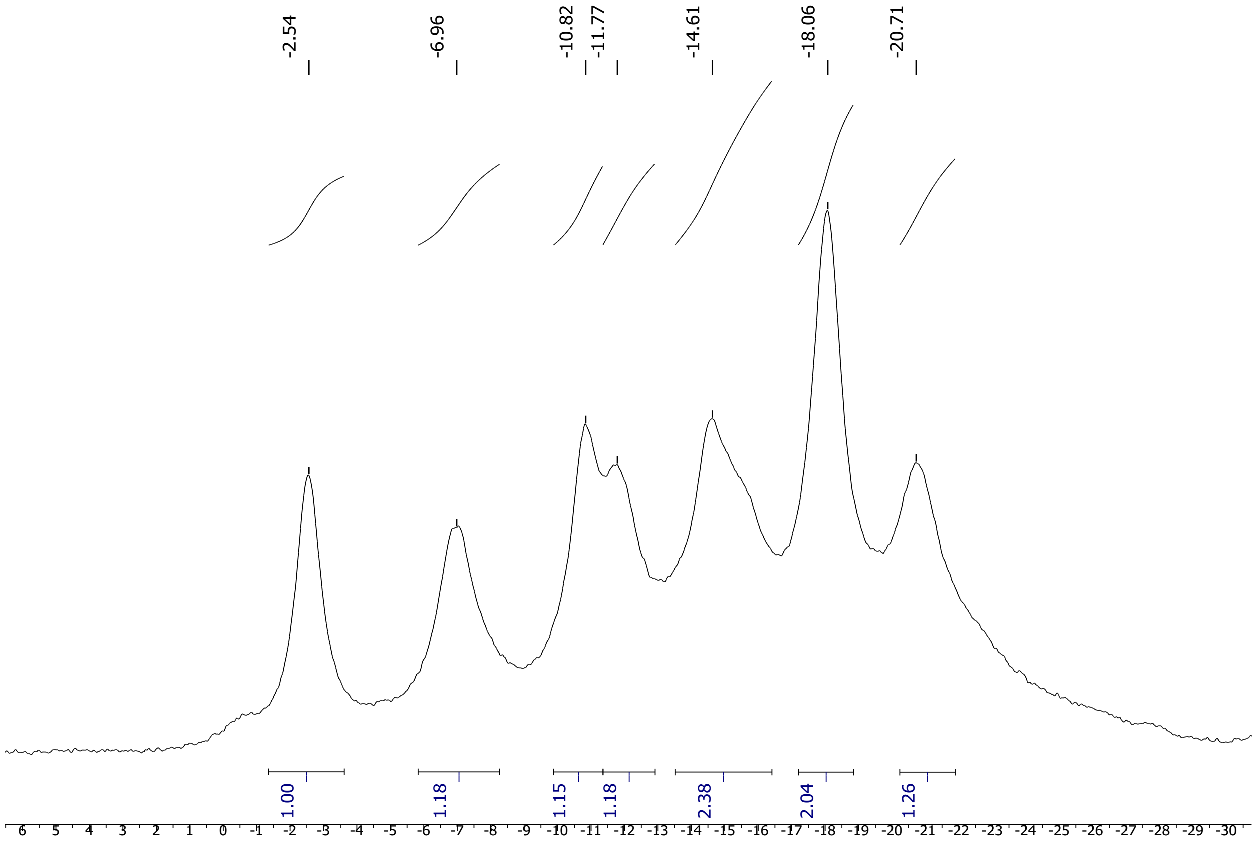

Figure S48. $^{11}B\{^{1}H\}$ NMR spectrum of **8** in acetone-d$_6$

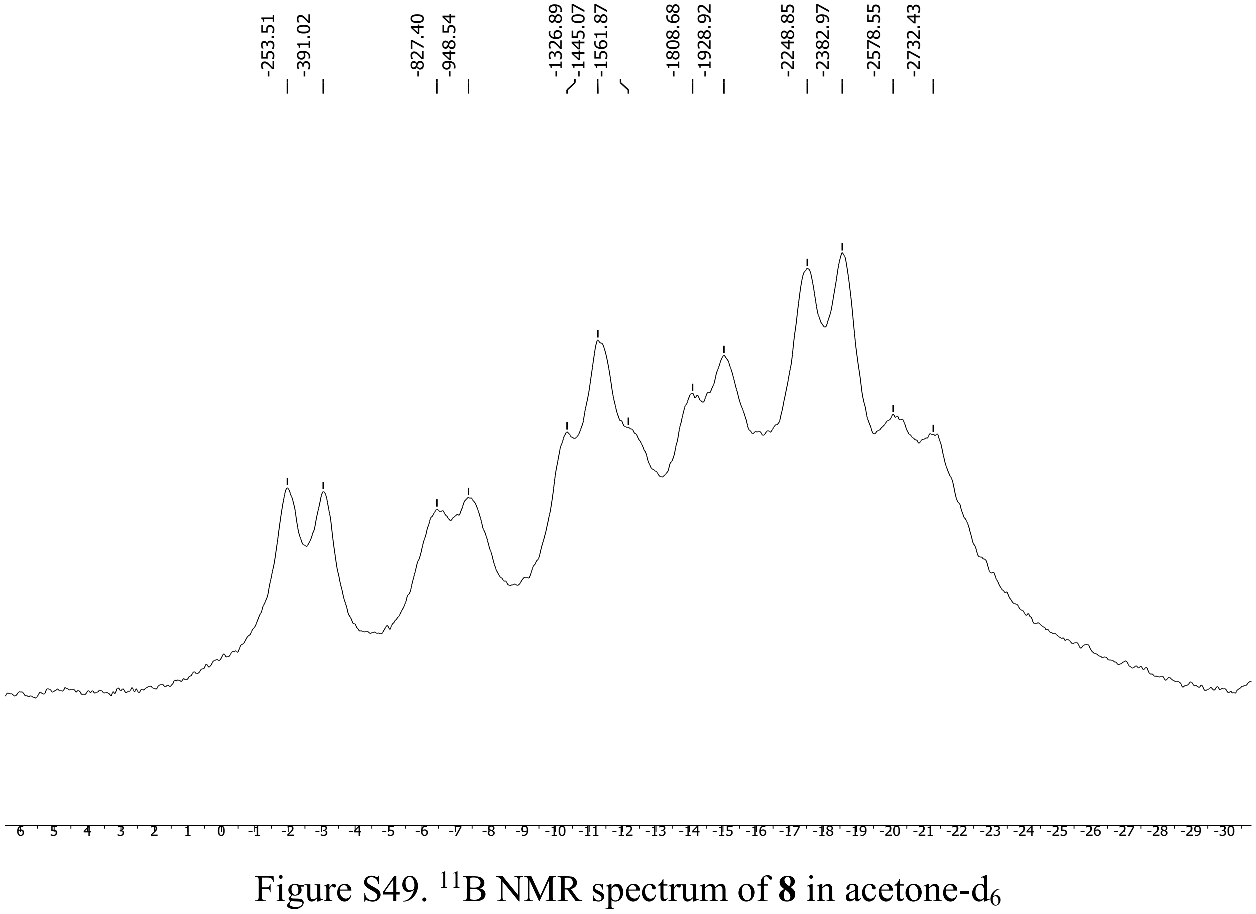

Figure S49. ^{11}B NMR spectrum of **8** in acetone-d$_6$

\

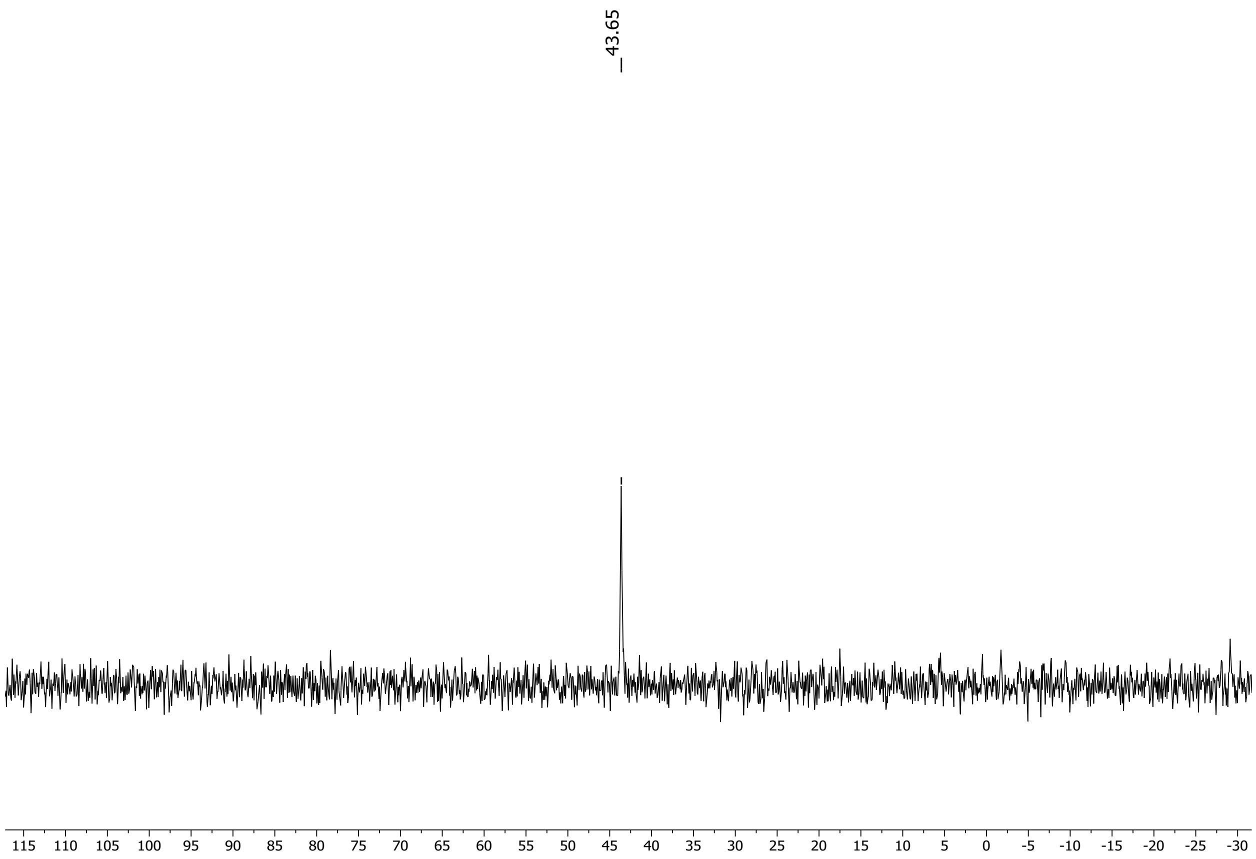

Figure S50. ^{31}P NMR spectrum of **8** in acetone-d_6

Spectral data for 3-Ph_3P-3-(1(2)-NC_5H_4-2'-S)-*closo*-3,1,2-$PdC_2B_9H_{10}$ (**9**)

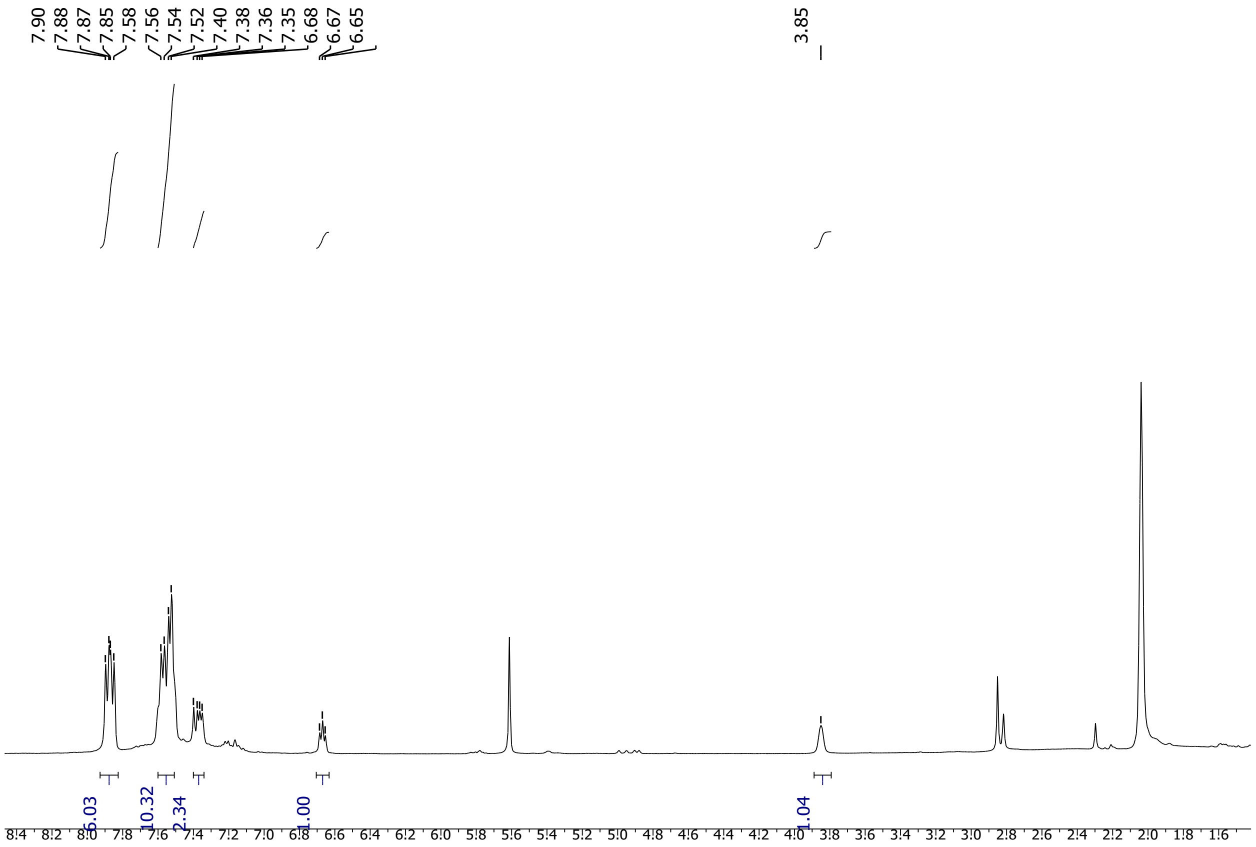

Figure S51. ^{1}H NMR spectrum of **9** in acetone-d_6

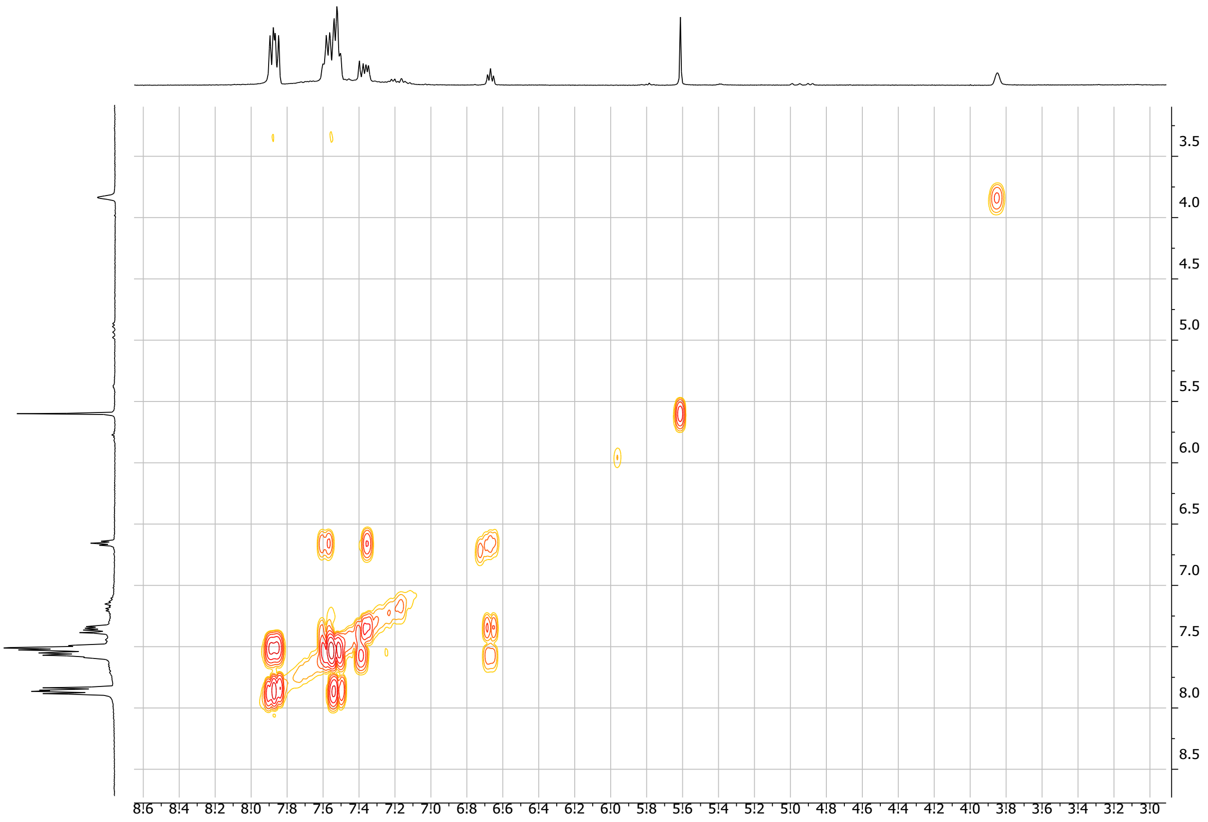

Figure S52. (HH) COSY NMR spectrum of **9** in acetone-d_6

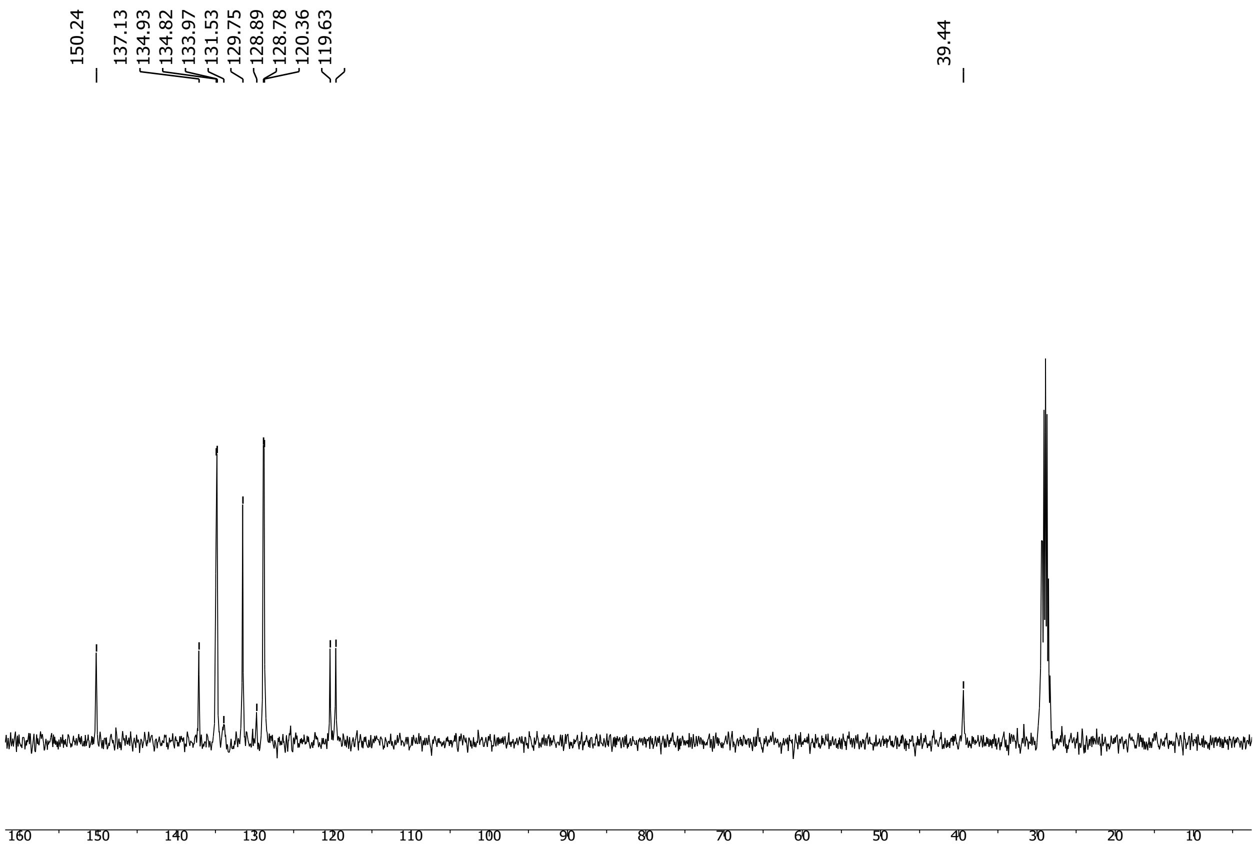

Figure S53. ^{13}C NMR spectrum of **9** in acetone-d_6

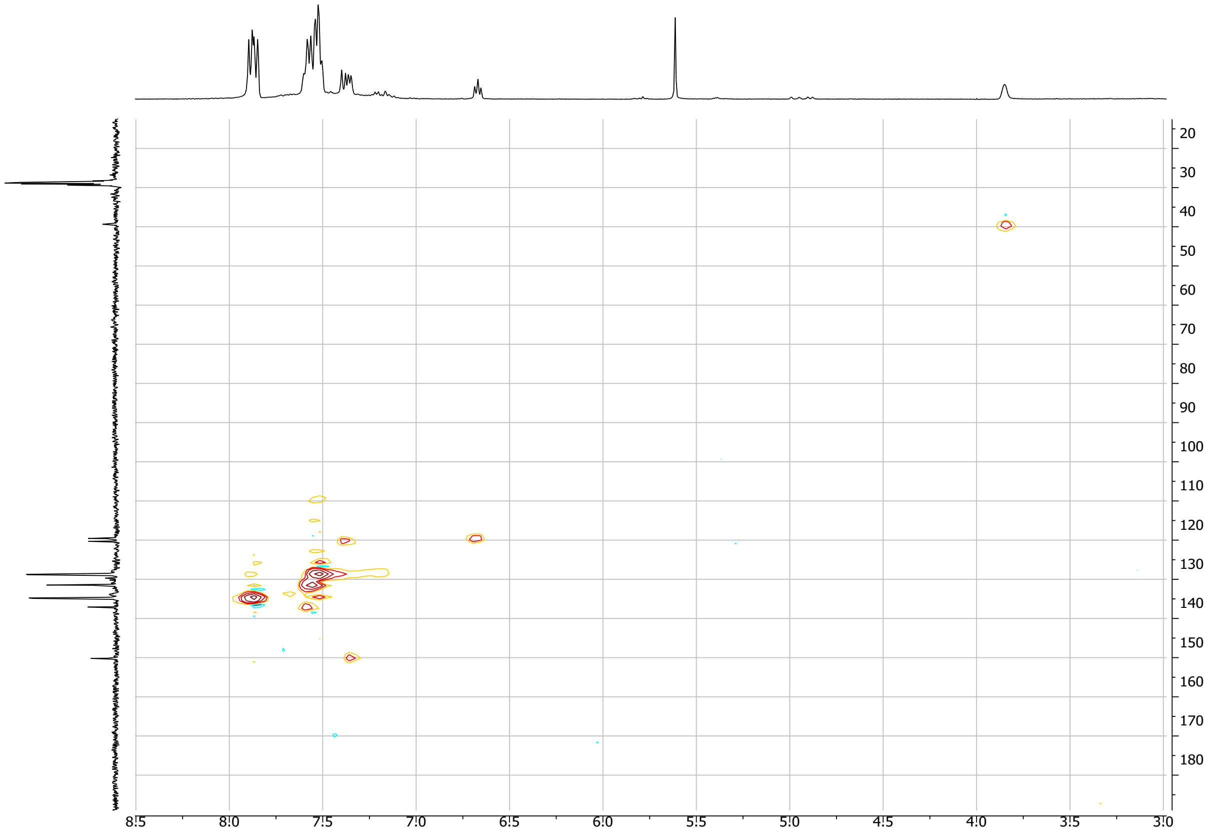

Figure S54. (HC) HSQC NMR spectrum of **9** in acetone-d_6

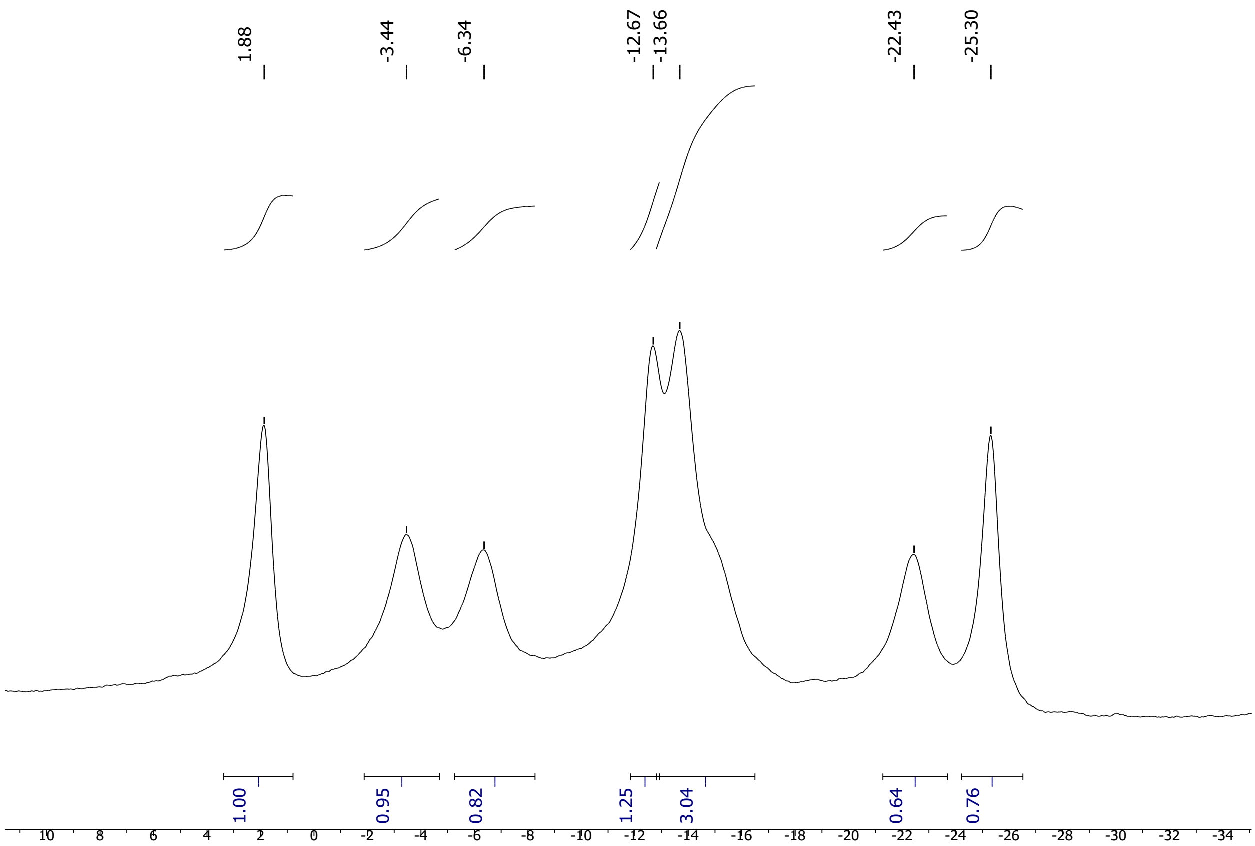

Figure S55. $^{11}B\{^{1}H\}$ NMR spectrum of **9** in acetone-d$_6$

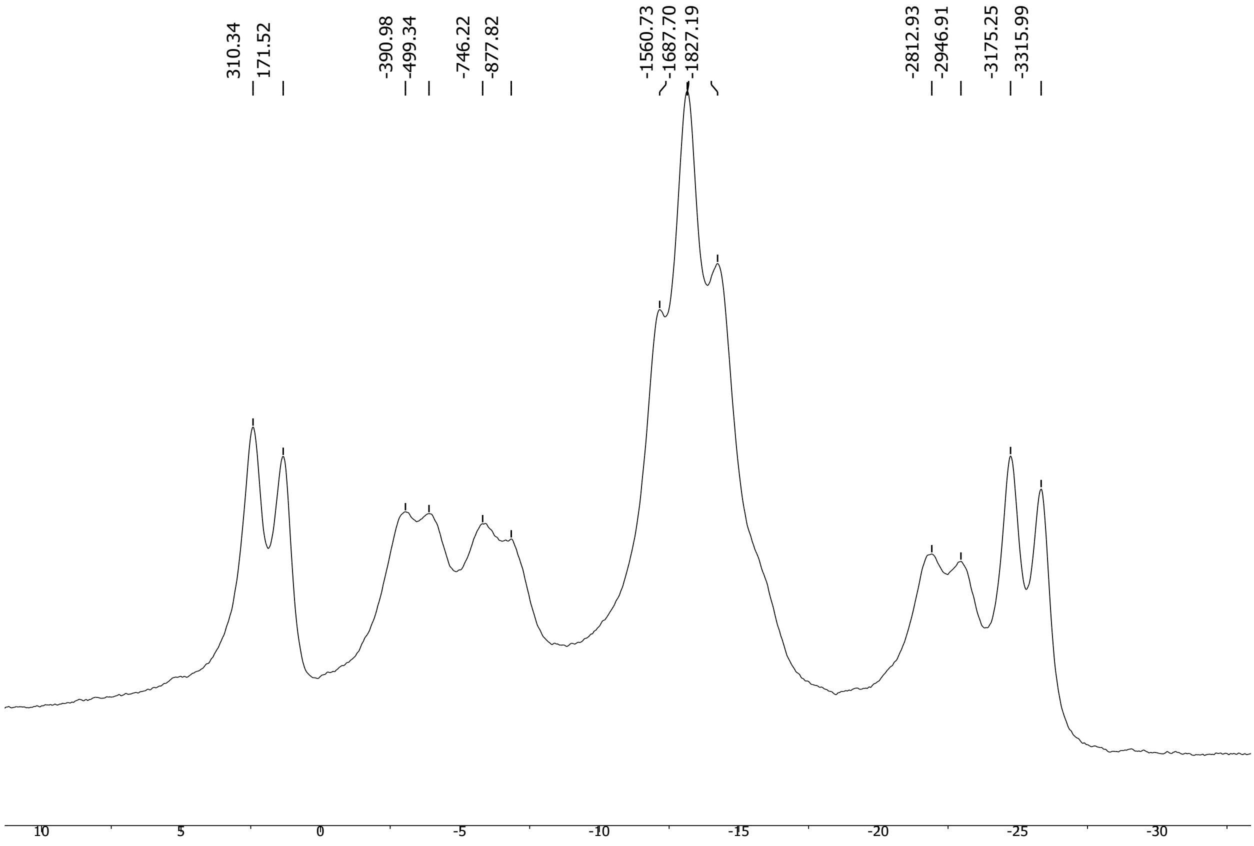

Figure S56. ^{11}B NMR spectrum of **9** in acetone-d$_6$

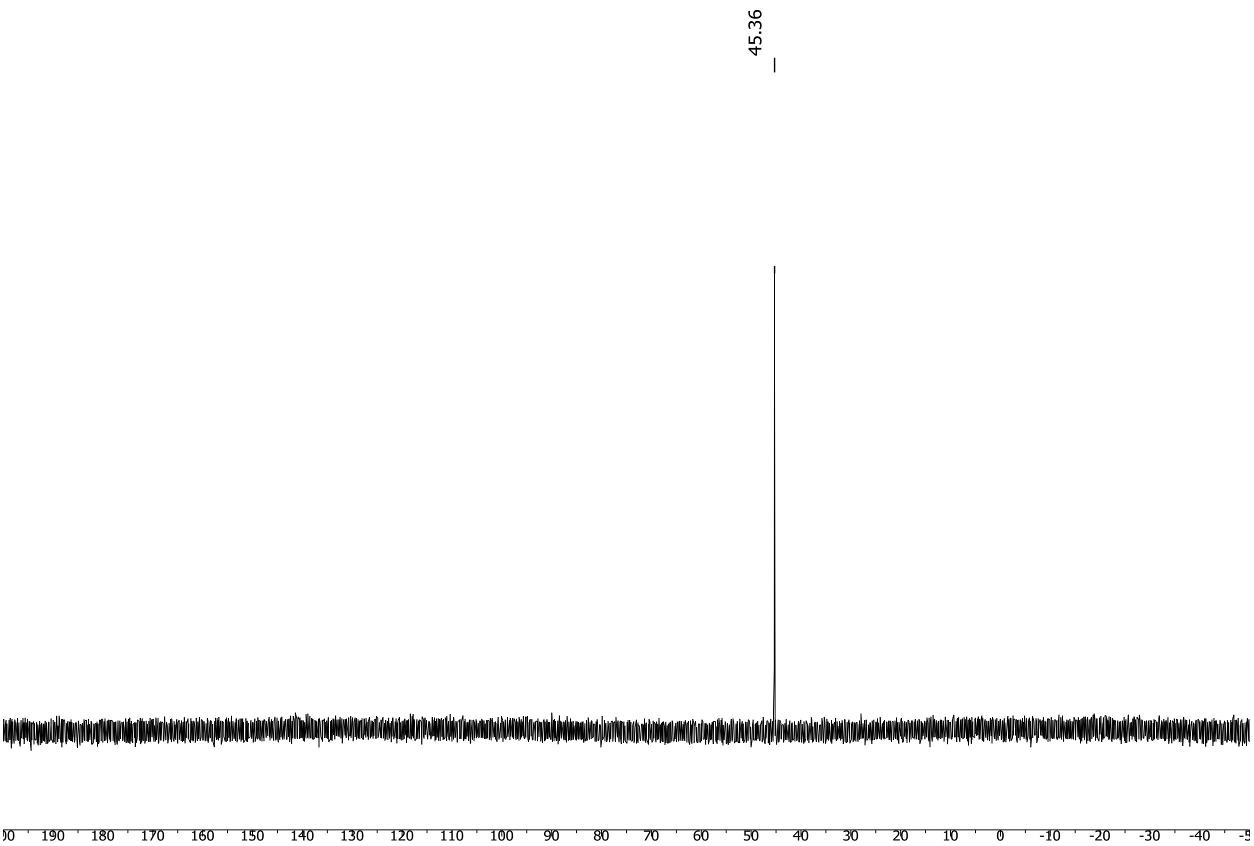

Figure S57. ^{31}P NMR spectrum of **9** in acetone-d_6

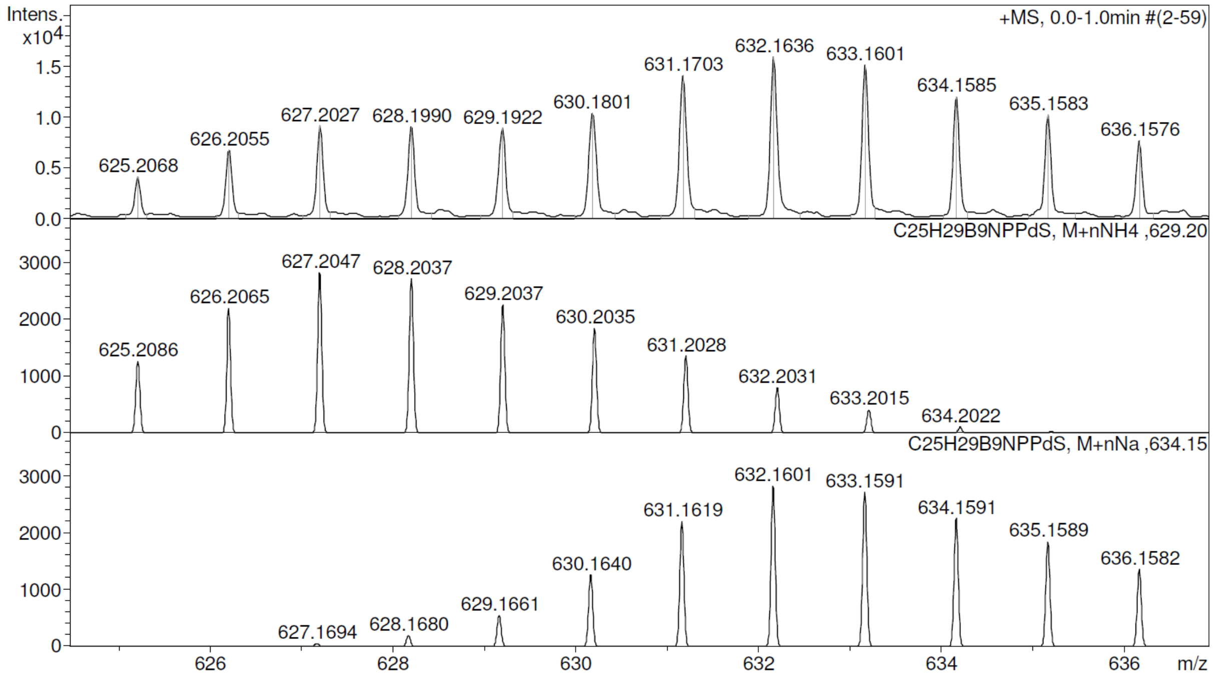

Figure S58. HRMS spectrum of **9**

Spectral data for 3-Ph_3P-3-(1(2)-NC_5H_4-2'-CH_2S)-*closo*-3,1,2-$NiC_2B_9H_{10}$ (**10**)

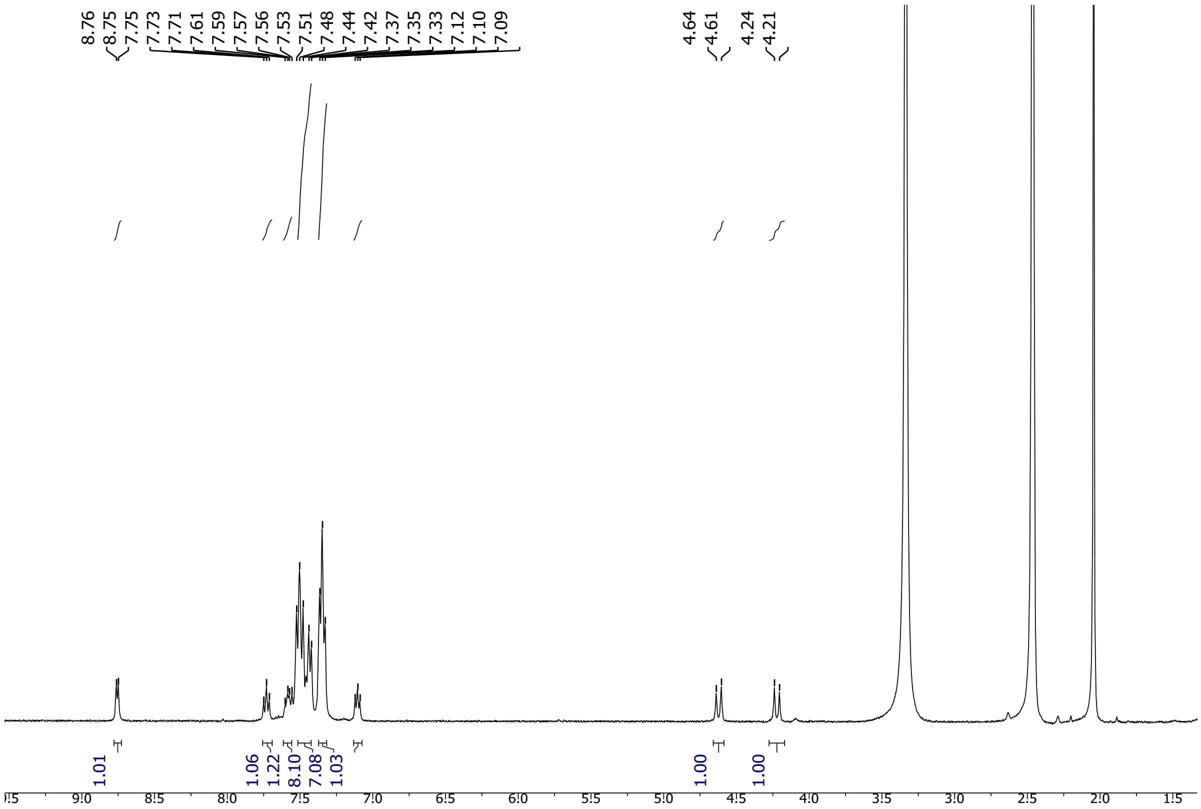

Figure S59. ^{1}H NMR spectrum of **10** in DMSO-d_6

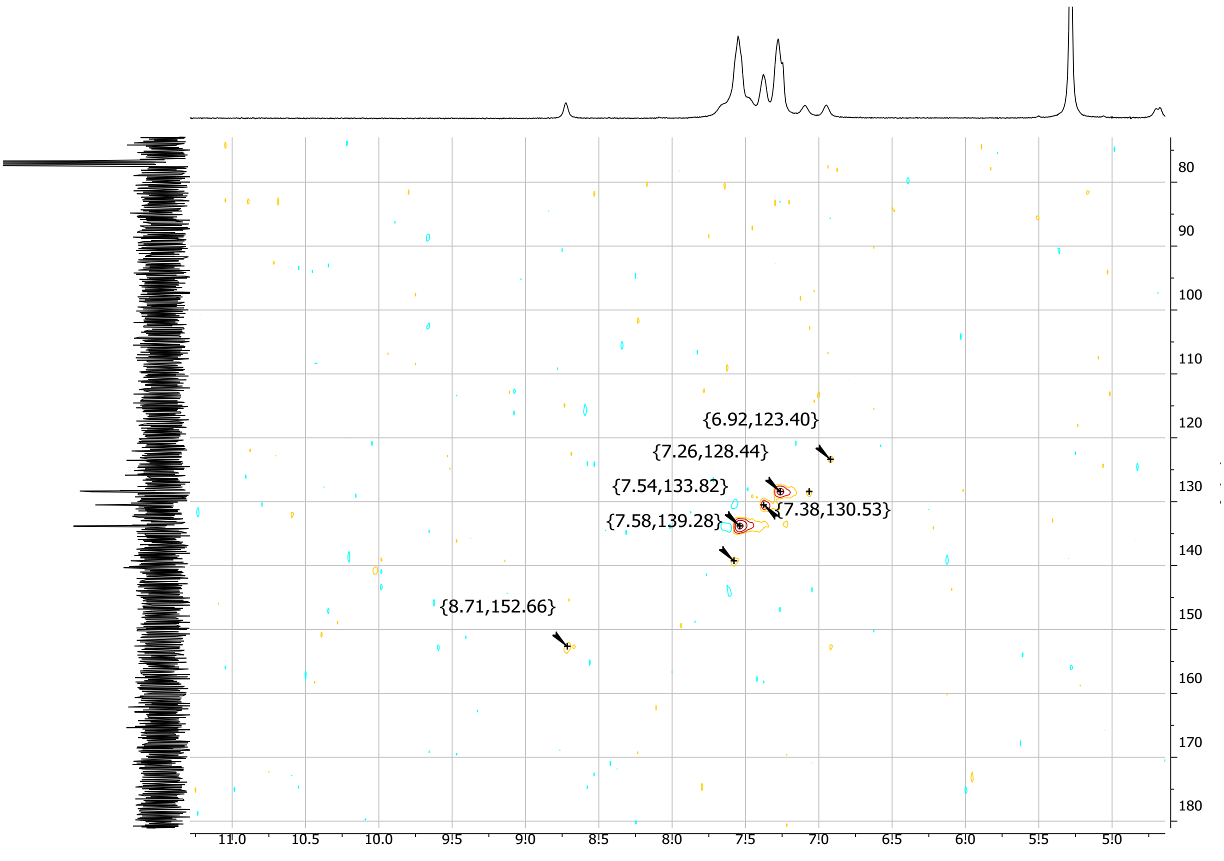

Figure S60. (HC) HSQC NMR spectrum of **10** in $CDCl_3$

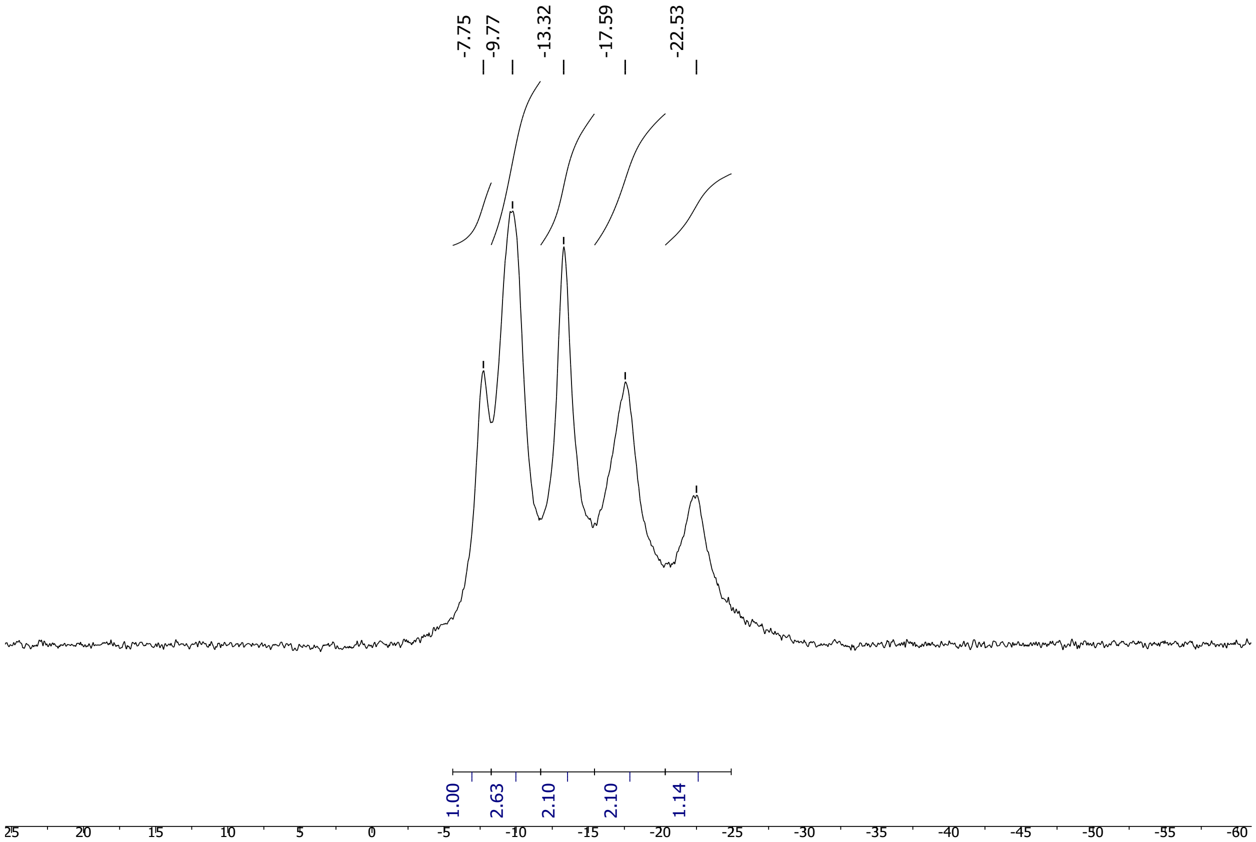

Figure S61. $^{11}B\{^{1}H\}$ NMR spectrum of **10** in $CDCl_3$

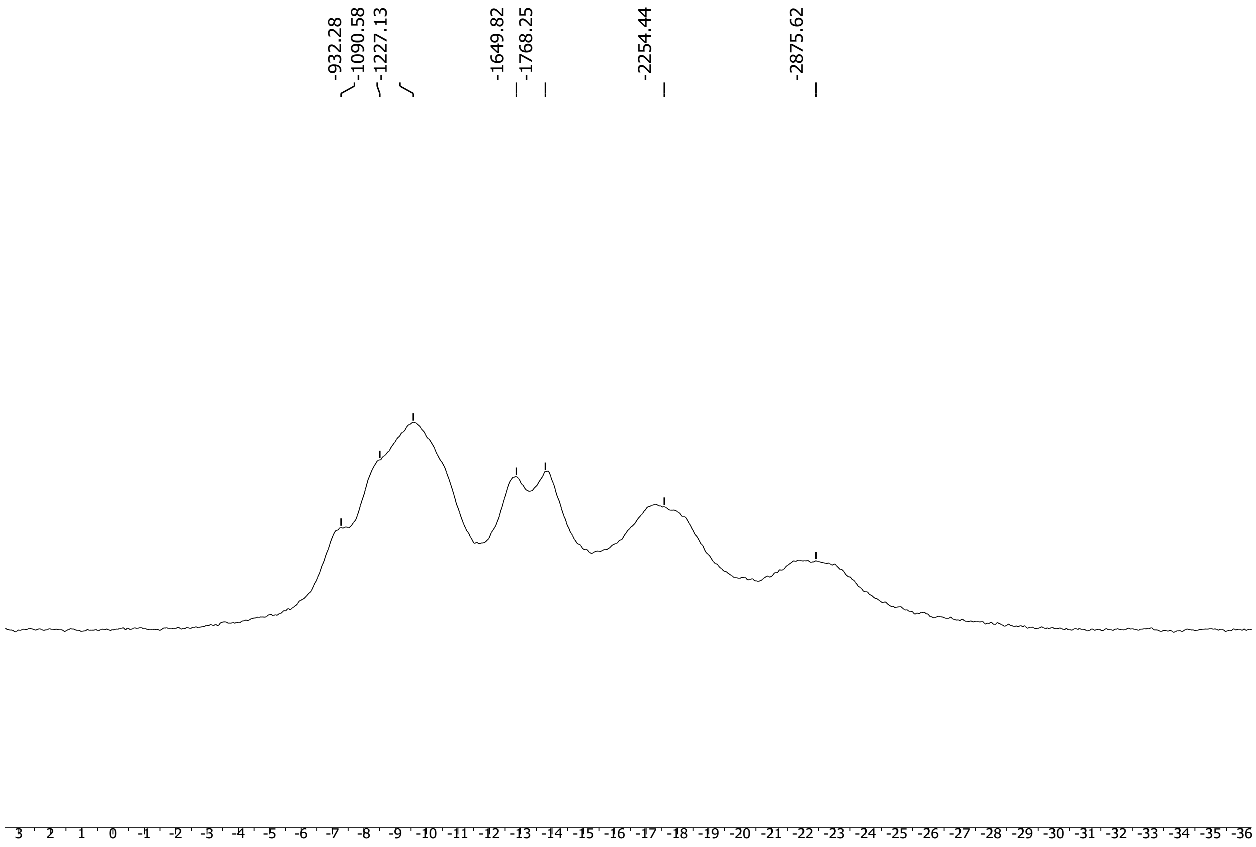

Figure S62. ^{11}B NMR spectrum of **10** in $CDCl_3$

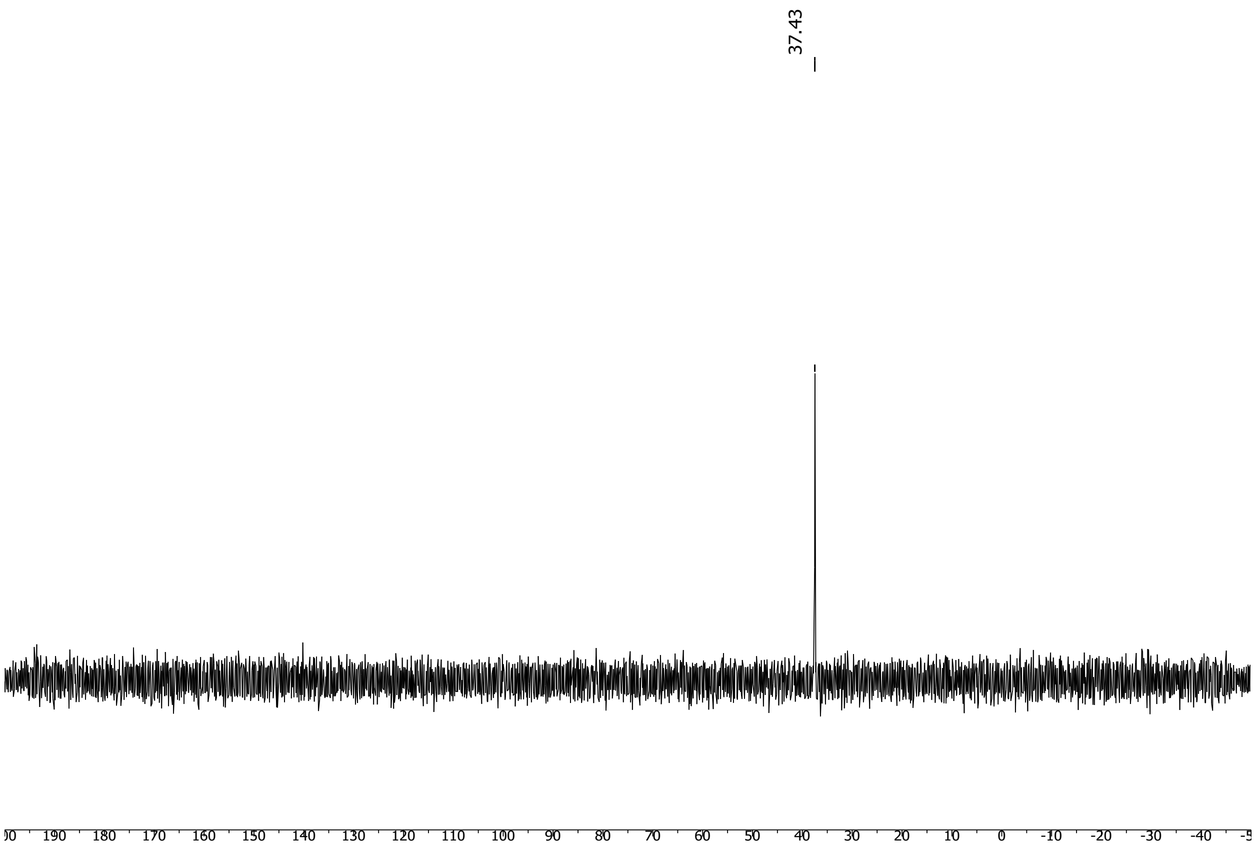

Figure S63. ^{31}P NMR spectrum of **10** in $CDCl_3$

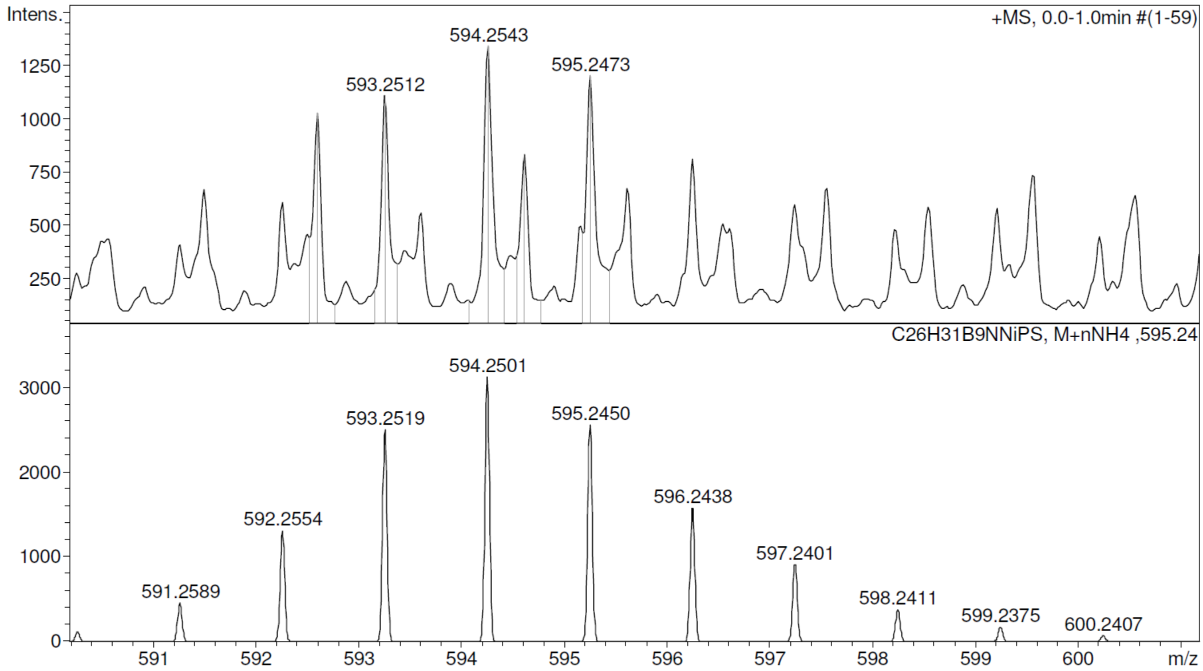

Figure S64. HRMS spectrum of **10**

Spectral data for 3-Ph_3P-3-(1(2)-NC_5H_4-2'-CH_2S)-*closo*-3,1,2-$PdC_2B_9H_{10}$ (**11**)

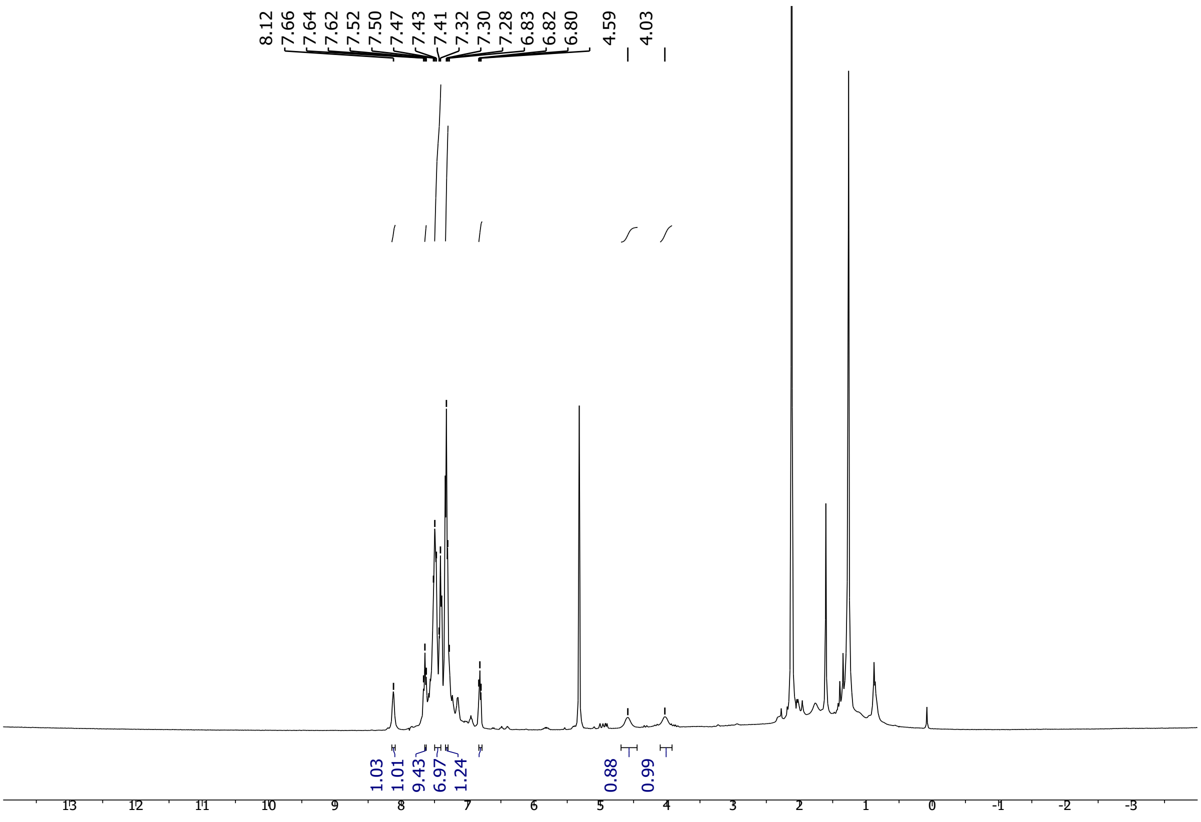

Figure S65. ^{1}H NMR spectrum of **11** in CD_2Cl_2

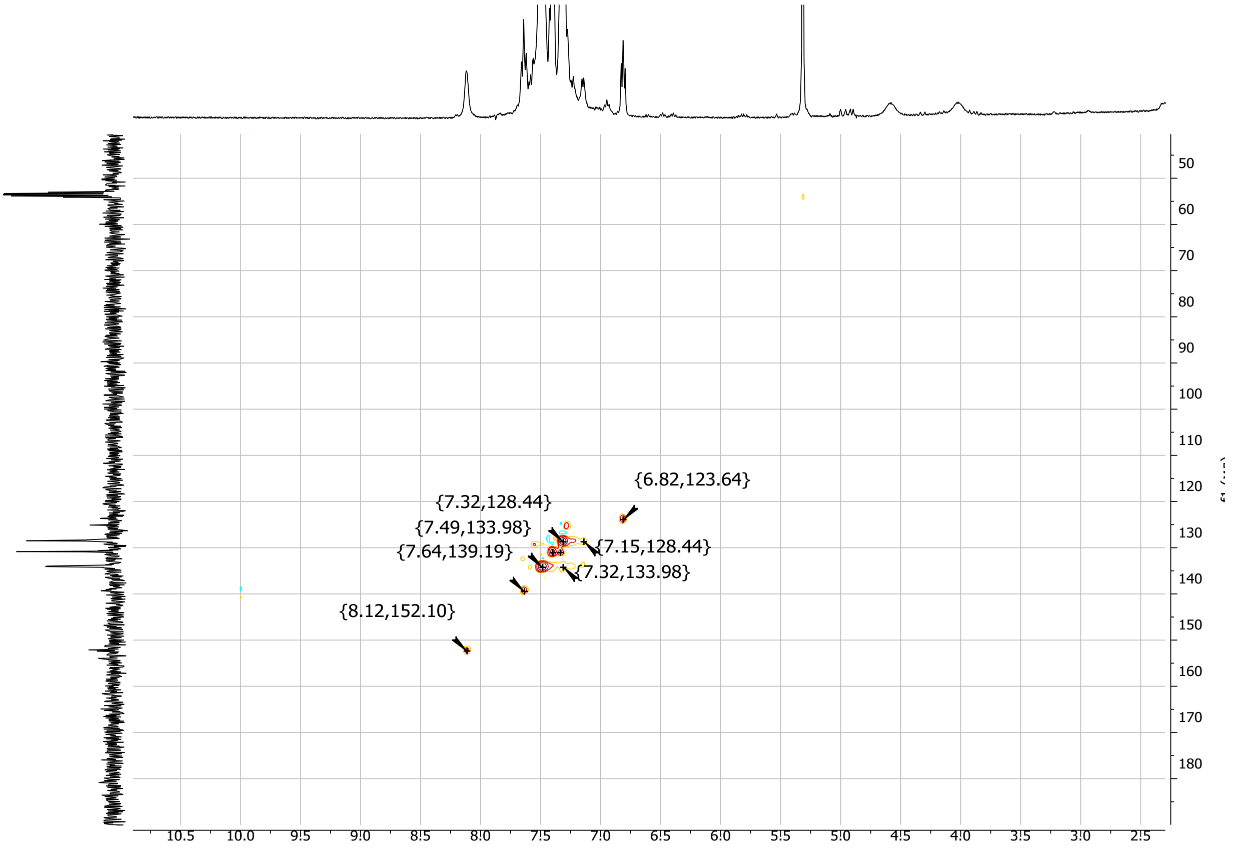

Figure S66. (HC) HSQC NMR spectrum of **11** in CD_2Cl_2

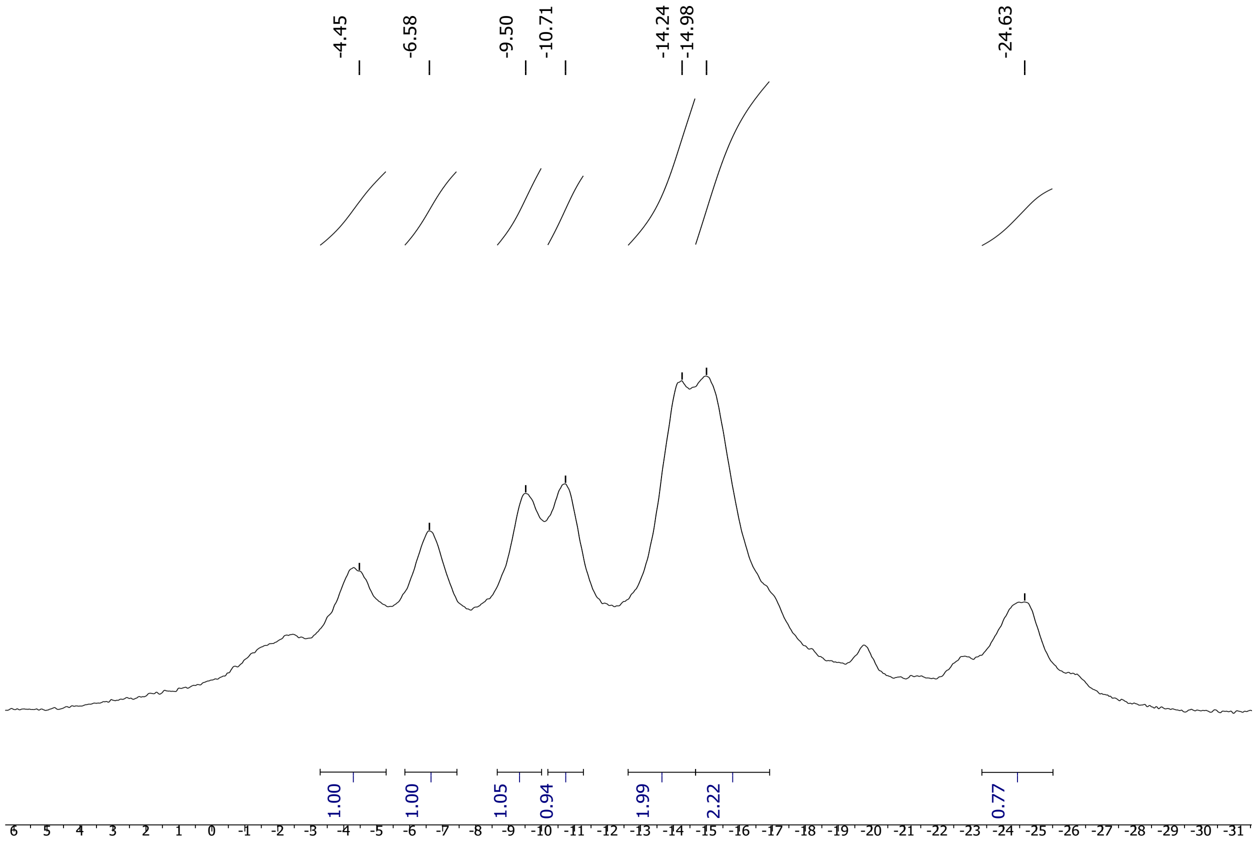

Figure S67. $^{11}B\{^{1}H\}$ NMR spectrum of **11** in CD_2Cl_2

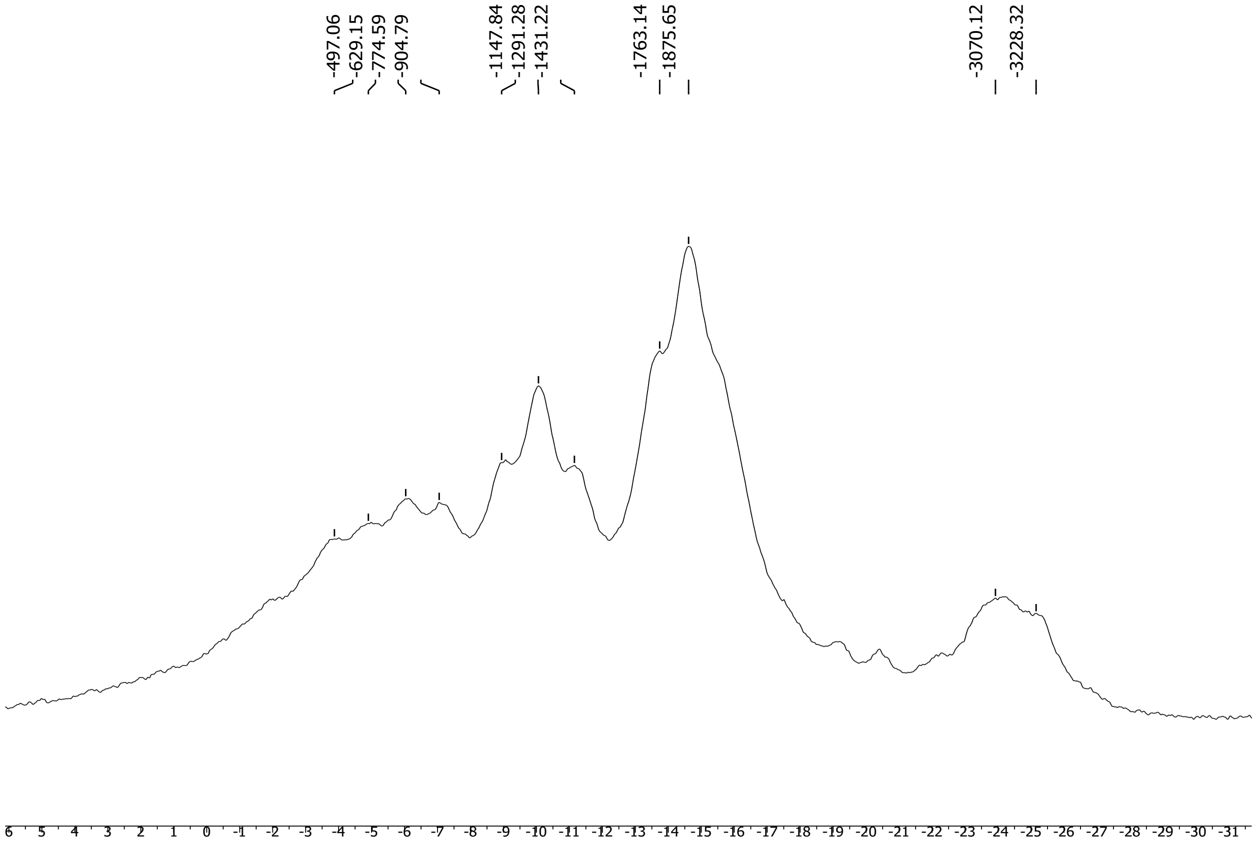

Figure S68. ^{11}B NMR spectrum of **11** in CD_2Cl_2

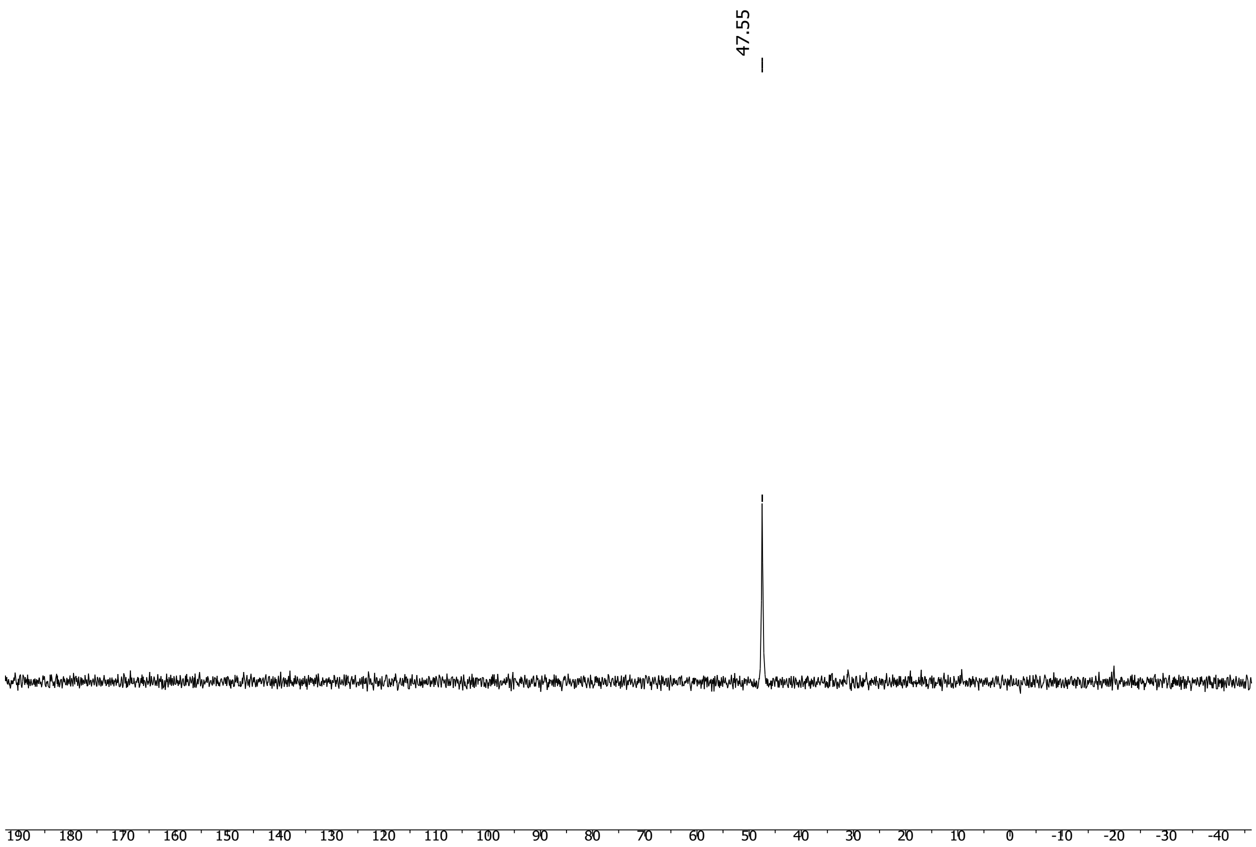

Figure S69. ^{31}P NMR spectrum of **11** in CD_2Cl_2

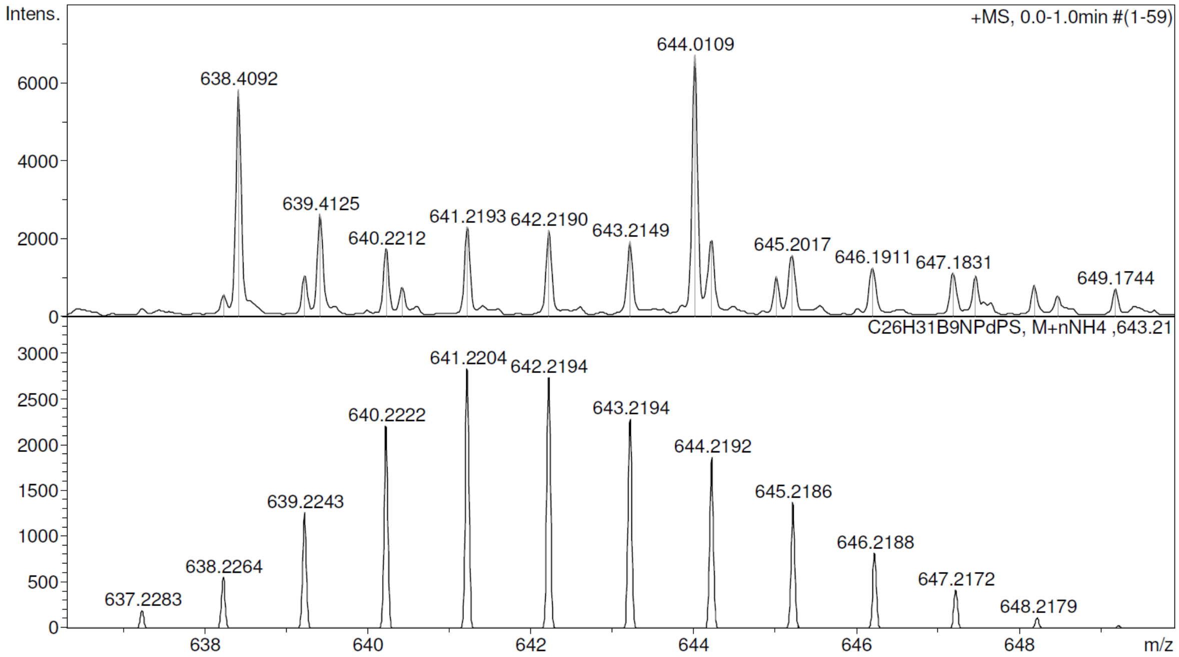

Figure S70. HRMS spectrum of **11**

Table S1. Crystallographic data for compounds **2*HBr**, **4**, **Cs[5]*0.5Me₂CO**, **8**, and **10**.

	**4**	**Cs[5]**	**2*HBr**	**8**	**10**
formula	$C_8H_{17}B_{10}NS$	$C_8H_{17}B_9NS^-$ $Cs^+\cdot½(C_3H_6O)$	$C_8H_{17}B_9NS^+Br^-$	$C_{25}H_{29}B_9NNiPS$	$C_{26}H_{31}B_9NNiPS$
fw	267.38	418.52	418.52	562.52	576.55
crystal system	Monoclinic	Triclinic	Orthorhombic	Monoclinic	Orthorhombic
space group	$P2_1/n$	P-1	$Pna2_1$	$P2_1/n$	$P2_12_12_1$
a, Å	11.8345(13)	7.9686(5)	24.234(3)	10.6347(3)	8.489(3)
b, Å	6.8603(7)	8.8768(6)	8.7044(9)	19.0306(4)	18.066(5)
c, Å	17.5569(19)	13.7553(9)	7.4298(8)	14.0144(3)	18.928(7)
α, deg.	90	99.784(2)	90	90	90
β, deg.	90.078(3)	102.455(2)	90	104.6870(10)	90
γ, deg.	90	107.360(2)	90	90	90
V, $Å^3$	1425.4(3)	877.69(10)	1567.3(3)	2743.62(11)	2902.7(18)
Z	4	2	4	4	4
d_{cryst}, g·cm^{-3}	1.246	1.584	1.417	1.362	1.319
F(000)	552	408	664	1160	1192
μ, mm^{-1}	0.202	2.217	2.733	0.860	0.814
θ range, deg.	1.16 – 27.20	2.48 – 28.15	2.49 – 28.03	2.25 – 26.97	2.15 – 26.05
independent rflns	3171	4276	3775	5968	5521
R_{int}	0.0447	0.0239	0.0474	0.0539	0.1380
Completeness to θ, %	99.8	99.7	99.8	99.9	96.9
refined parameters	226	213	187	383	351
GOF (F^2)	1.040	1.111	1.078	1.038	0.952
rflns with I>2σ(I)	2904	3980	3379	4610	2702
$R_1(F)$ (I>2σ(I))[a]	0.0275	0.0230	0.0297	0.0298	0.0822
$wR_2(F^2)$ (all data)[b]	0.0688	0.0603	0.0692	0.0683	0.1986
Largest diff. peak/hole, e·Å^{-3}	0.163/-0.218	0.814/-0.888	0.400/-0.315	0.319/-0.275	0.722/-1.139

[a] $R_1 = \sum|F_o - |F_c||/\sum(F_o)$; [b] $wR_2 = (\sum[w(F_o^2 - F_c^2)^2]/\sum[w(F_o^2)^2]^{½}$
